# Bioinspired Mechanisms and Actuation of Soft Robotic Crawlers

**DOI:** 10.1002/advs.202416764

**Published:** 2025-03-20

**Authors:** Min Pan, Miaomiao Liu, Jiayi Lei, Yunyi Wang, Changhong Linghu, Chris Bowen, K. Jimmy Hsia

**Affiliations:** ^1^ Department of Mechanical Engineering University of Bath Bath BA2 7AY UK; ^2^ School of Mechanical and Aerospace Engineering Nanyang Technological University 50 Nanyang Avenue Singapore 639798 Singapore; ^3^ School of Chemistry Chemical Engineering and Biotechnology Nanyang Technological University 50 Nanyang Avenue Singapore 639798 Singapore

**Keywords:** actuation mechanisms, bioinspired robots, locomotion, soft crawlers

## Abstract

Inspired by soft‐bodied animals, scientists and roboticists have explored and created soft materials and robots in this last decade to mimic the functionality and behavior of soft‐crawling animals. Such effort has originated from the unique ability of soft structures to crawl effectively in complex natural environments. As a result, there is growing interest in the design of highly functional soft robotic crawlers through the creation of new flexible functional materials, understanding the underlying science of crawling mechanisms, and employing advanced actuation strategies. This review investigates the current state‐of‐the‐art in this fascinating area to demonstrate the nexus between materials, mechanisms, actuation, and applications. Bioinspired crawling mechanisms of soft crawlers are initially outlined, which include two‐anchor peristaltic and serpentine crawling and undulatory motion as analogs to caterpillars, worms, and snakes, respectively. The fabrication and use of new materials in the design of soft crawlers are also discussed, along with the exploitation of actuation mechanisms to achieve specific crawling locomotion. Finally, insights into future research directions are outlined.

## Graphical abstract

Researchers have been developing soft materials and robotic crawlers inspired by soft‐bodied animals to mimic effective crawling in complex environments. This review explores the state‐of‐the‐art in soft crawlers, highlighting the interaction between materials, crawling mechanisms, actuation, and applications. The review concludes by providing insights into prospective research directions in this emerging field.

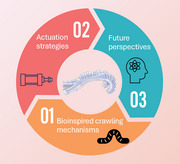

## 1 Introduction

Soft robotics is a rapidly evolving field that leverages the unique properties of flexible and adaptive materials to mimic biological motion and functionalities. Among the various types of soft robots, **soft crawlers** have garnered significant attention due to their potential in applications requiring delicate interactions with their environment, such as biomedical devices, exploratory robotics, and minimally invasive surgeries. These robots are inspired by natural crawling mechanisms observed in soft‐bodied animals, and their design and functionality are underpinned by advances in materials science, innovative crawling mechanisms, and diverse actuation strategies.

Recent advancements in functional materials specifically engineered for soft crawlers have significantly expanded the potential for developing systems that demonstrate exceptional flexibility, adaptability, and multifunctionality. These innovative materials allow soft crawlers to perform complex deformations and achieve versatile locomotion by responding dynamically to a range of external stimuli, including heat, light, electric fields, and pneumatic actuation. The range of crawling mechanisms and actuation strategies developed for soft crawlers offers significant advantages, including enhanced adaptability to unstructured environments and the ability to navigate confined or delicate spaces. However, challenges remain, including limitations in speed, energy efficiency, and durability. Understanding these trade‐offs is crucial for advancing the field. This review synthesizes the current state‐of‐the‐art soft crawler technologies, assessing their advantages and limitations while identifying gaps and challenges that require further exploration.

This review is motivated by several key factors that distinguish it from previous works in the field of soft robotics. Notably, the most cited review on soft robotics was published by *Nature* in 2015 (Google citation: 5532)^[^ ^1^ ^]^ which provided a broad overview of the design, fabrication, and control of soft robots. Since then, more focused reviews have emerged, addressing topics such as the manufacturing of soft robots,^[^ ^2^ ^]^ soft robotic sensing,^[^ ^3^ ^]^ control strategies,^[^ ^4^ ^]^ and research challenges.^[^ ^5^, ^6^ ^]^ However, a dedicated examination of the **functional material properties**, **crawling mechanisms**, and **actuation strategies** specific to soft crawlers is lacking. This review fills this gap, offering a unique perspective with limited overlap with existing literature. We provide a comprehensive analysis of these advanced materials, focusing on their unique properties, such as elasticity, responsiveness, and durability. We examine how these characteristics enable various crawling mechanisms, facilitating movement that mimics natural organisms. Additionally, we discuss the integration of these materials into soft crawler designs, highlighting their implications for applications in robotics, biomedical devices, and soft automation. By examining current research and future directions, this review aims to provide insights into the evolving landscape of soft robotics and the critical role that functional materials play in its advancement.

The diversity of locomotion strategies enabled by new materials and actuation approaches highlights the untapped potential of soft robots. While capabilities such as crawling, hopping, flying, and swimming are being explored, the interplay between material properties and the realization of specific locomotion and actuation mechanisms remains under‐discussed. We aim to bridge this gap by analyzing the underlying connections between functional materials, crawling mechanisms inspired by soft‐bodied organisms, and actuation strategies that enable desired locomotion. We will investigate *bioinspired mechanisms* focusing on robot designs, structures, and systems and drawing inspiration from biological organisms and natural phenomena. These designs aim to enhance the efficiency, adaptability, and functionality of the soft crawlers. Often, they replicate the movements, materials, or structural properties found in living organisms to address complex engineering challenges. *Bioinspired actuation* involves systems that mimic or draw inspiration from how biological organisms produce motion. These systems utilize smart materials, soft robotics, or innovative mechanical designs to achieve flexible, efficient, and adaptive movement, similar to the function of muscles or the natural locomotion found in animals. Finally, this review provides the authors' insights into future directions and challenges in developing soft crawlers. From advancements in material science to integrating innovative actuation methods, the field presents numerous opportunities for breakthroughs, particularly in areas requiring effective operation under extreme environments and in biomedical and healthcare applications.^[^ ^7^ ^]^ By addressing these aspects, this review provides insights to researchers in overcoming existing challenges and unlocking the full potential of the next generation of soft crawlers.

## 2 Bioinspired Crawling Mechanisms

Crawling animals, which are mainly composed of soft and flexible tissues, provide valuable insights for the development of mobile soft robots. This type of locomotion, known as crawling, encompasses a variety of gaits as well as seemingly chaotic body movements that enable locomotion; a familiar example of this is the way infants crawl. Although crawling techniques can vary widely, most of them rely on friction as a primary means of movement. We delve into specific crawling gaits that have been identified in various organisms, including caterpillars, earthworms, and snakes. These creatures exhibit specialized forms of crawling that can be adapted into robotic designs. For instance, caterpillars typically use a looping motion, while worms employ a more extended, undulating movement. Snakes demonstrate a unique ability to move across different surfaces through lateral undulation or rectilinear movement. Additionally, the mechanisms of these fundamental crawling movements inform the creation of soft crawling robots. By mimicking the efficient and adaptable locomotion strategies found in nature, researchers can design robotic crawlers capable of navigating complex terrains. The newly developed functional materials can enhance the performance of these soft crawlers, allowing them to mimic the natural motion of their biological counterparts effectively.

### 2.1 Two‐Anchor Crawling Mechanism

**Figure 1:**
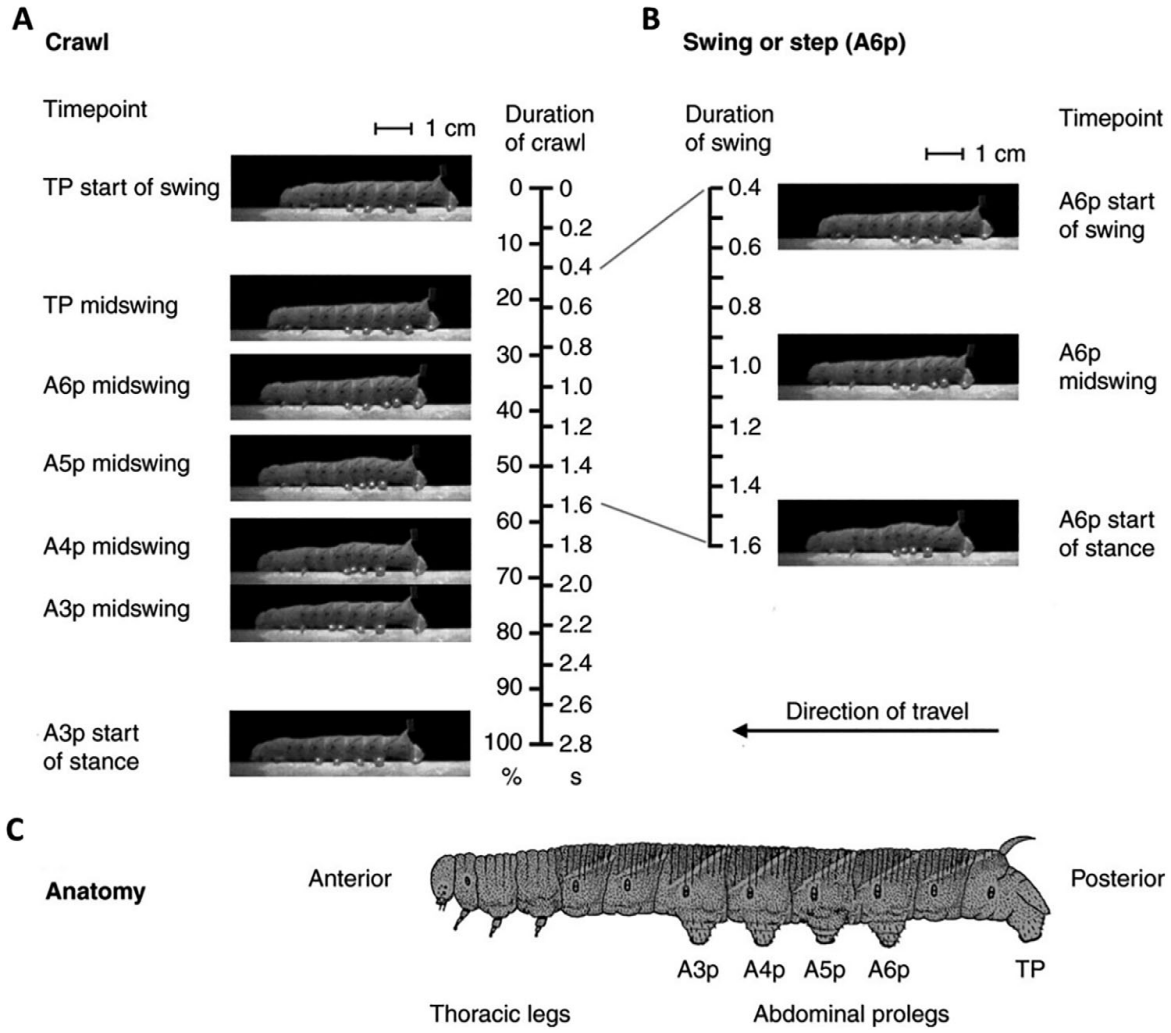
Caterpillar crawling mechanism. A) A full crawl starts with the terminal prolegs (TP) lifting away from the substrate. Each proleg is lifted and placed forward. The proleg on abdominal segment three (A3p) touching down is defined as the end of the crawl. In this individual, a crawl lasted 2.8 s. B) The swing phase for the proleg on abdominal segment A6 (A6p), starting with the start of the swing phase and ending with the start of the stance phase. C) Anatomy of a fifth instar of the Manduca sexta larva. Reproduced from van Griethuijsen and Trimmer.^[^ ^8^ ^]^ Copyright 2009, The Company of Biologists.

**Figure 2:**
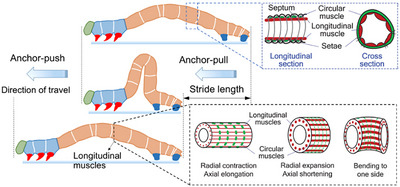
Locomotion of a caterpillar for one stride. Reproduced from Wang et al.^[^ ^9^ ^]^ Copyright 2014, IOP Publishing and Zhan et al.^[^ ^10^ ^]^ Copyright 2019, Sage Publications.

We use a *caterpillar* as an example, which has been extensively studied in the field of biology for its two‐anchor locomotion mechanism to investigate the potential of new functional materials that are suitable for soft crawlers.^[^ ^8^ ^]^ Soft‐bodied animals such as caterpillars are excellent crawlers. *Manduca sexta* (Linnaeus 1763) explains caterpillars crawl with the tip of their abdomen, the terminal prolegs (TP), and continue with an anterior grade wave of steps with its remaining four pairs of prolegs, as shown in **Figure** **1**. The locomotion of a caterpillar for one stride is defined as a *two‐anchor crawling gait* ^[^ ^9^ ^]^ (see **Figure** **2**). The caterpillar travels by extending and contracting its body on the basis of anisotropic friction. Using this mechanism, forward linear crawling locomotion is enabled when the forward friction is lower than the backward friction, on the condition that its longitudinal muscle fibers are actuated symmetrically. When the longitudinal muscle fibers are actuated asymmetrically, the caterpillar performs a nonsymmetric deformation, which results in a turning locomotion using one of its feet as an anchor.^[^ ^9^ ^]^

The two‐anchor crawling mechanism is a locomotion strategy utilized by soft crawlers inspired by natural organisms like caterpillars. This mechanism operates by alternating between two distinct phases: anchoring and releasing. During the anchoring phase, one part of the robot secures itself to the surface, providing stability and support, while the other part moves forward. Once the forward movement is accomplished, the roles reverse, allowing the previously anchored section to release and progress. This rhythmic and coordinated movement enables the robot to efficiently navigate through tight and confined spaces and traverse challenging and uneven terrains with ease, much like its biological counterparts. This method is effective for maneuvering in confined spaces and on uneven surfaces. The soft crawler typically consists of two distinct areas, front and rear, alternately anchoring to the surface. Anchoring is accomplished through friction pads, suction cups, setae‐like structures, or surface roughness. During actuation, the crawler body elongates and contracts between two anchors, allowing the robot to move forward or backward. One anchor keeps the robot in place while the other progresses forward. The application of directional friction ensures that movement occurs only in the intended direction. Elastomers, thermoplastic polyurethanes (TPU), and hydrogels are used to develop the base body of crawlers. These crawlers can be actuated pneumatically, hydraulically, electrically, or magnetically. The critical anchoring materials for the generation of friction have been attempted. Some successful anchoring mechanisms include friction pads, suction cups, electroadhesive pads, and magnetic anchors.^[^ ^11^, ^12^, ^13^, ^14^ ^]^

Using two anchors, the crawling mechanism ensures one end remains attached to the surface while the other end can move, providing continuous stability during the crawling operation. This is particularly advantageous for navigating uneven or vertical surfaces, making it suitable for challenging environments like pipelines, walls, or underwater structures. Using alternating anchors can greatly decrease the need for continuous gripping or frequent adjustments. This mechanism consumes less energy compared to traditional continuous‐motion systems. By allowing for occasional rest or repositioning, alternating anchors help crawlers maintain efficiency over longer periods, reducing fatigue and improving overall productivity. Consequently, this approach saves energy and supports a more sustainable operational model. The two‐anchor crawling mechanism enables systematic and intentional movement, allowing for high positional accuracy. This is especially beneficial for tasks that require attention to detail, such as inspecting delicate areas or making repairs in confined or sensitive spaces. The carefully controlled movement reduces the risk of errors and ensures that each action is precise, which is essential for crawling robots working in challenging environments. In extreme or failing conditions, even if one anchor fails temporarily, the other can maintain attachment, preventing the crawlers from a total loss of grip.

The nature of the mechanism may result in slow crawling movement, and the crawler's structure requires precise synchronization between its two anchors. This adds complexity to both the mechanical and control systems. When using this crawling mechanism, the surfaces must adequately support strong anchoring. This limitation restricts its use on soft, slippery, or highly irregular surfaces where neither anchor can secure a proper attachment. As the movement is highly relying on the anchors, they are prone to wear and tear when the crawlers operate in abrasive or corrosive environments. The crawling nature is quite “discrete”, and it might be difficult to achieve fluid movement when needed.

### 2.2 Peristaltic Crawling Mechanism

**Figure 3:**
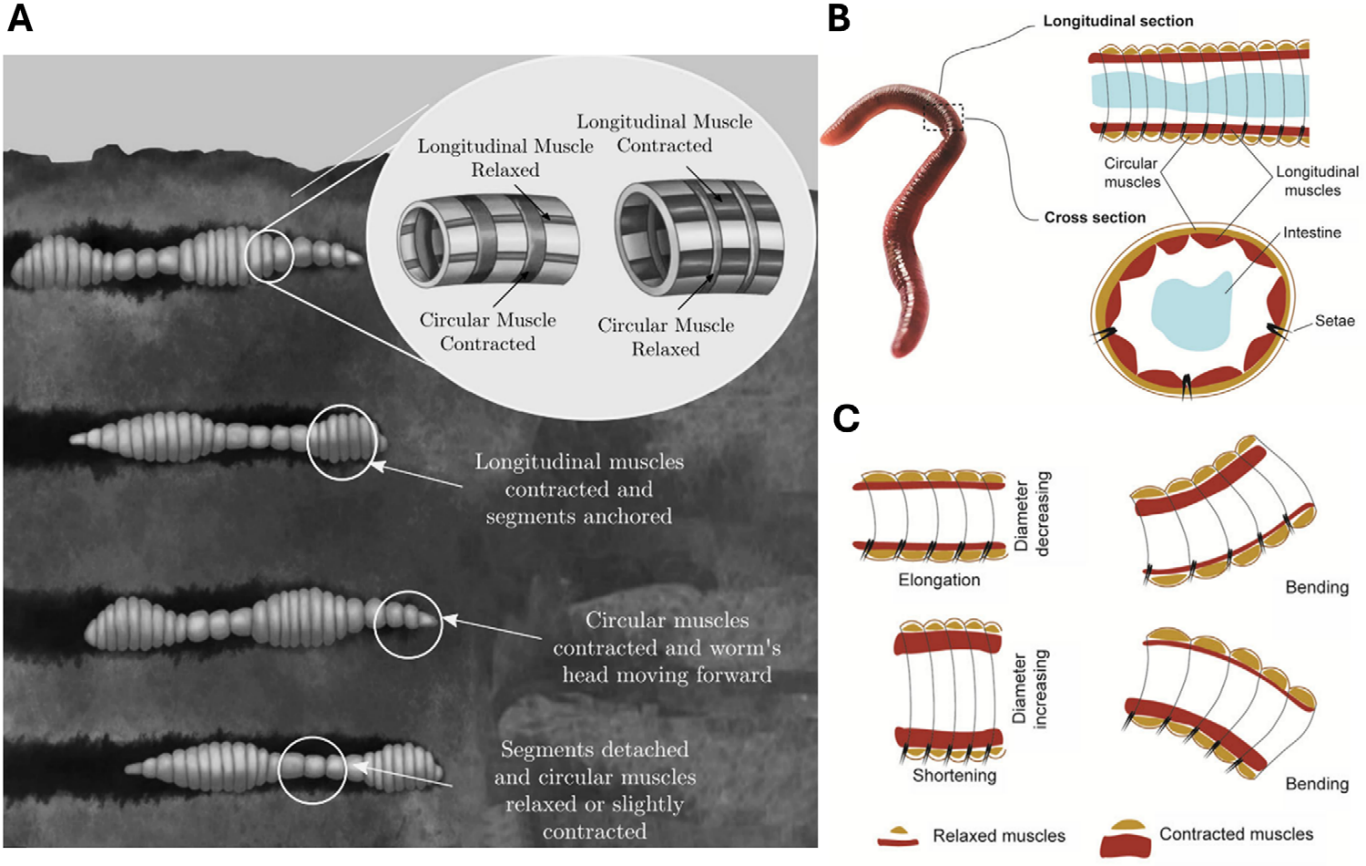
Earthworm crawling mechanism. A) Peristaltic locomotion of an earthworm during burrowing. Reproduced from Calderón et al.^[^ ^15^ ^]^ Copyright 2006, IOP Publishing. B) Schematic representation of the earthworm's longitudinal and cross‐sectional segments. Reproduced from Xiong et al.^[^ ^16^ ^]^ Copyright 2023, John Wiley & Sons. C) Schematic illustration of motion patterns and muscle states within the segments. Reproduced from Xiong et al.^[^ ^16^ ^]^ Copyright 2023, John Wiley & Sons.

We will take an *earthworm* as an example, which consists of a single or multiple muscular robots to achieve movement. Unlike the caterpillar, the body of the earthworm is not formed as a whole but is divided into separated muscular segments. The body of the earthworm is divided into ring‐like segments (annuli), each containing its own set of muscles. Inside each segment, there is a fluid‐filled coelom that serves as a hydrostatic skeleton, maintaining rigidity through the pressure of incompressible fluid. A wave of contraction and elongation of the muscles act to push the earthworm forward. This mechanism relies on the sequential contraction and relaxation of segments along the robot's body, enabling movement through confined or uneven spaces, as shown in **Figure** **3**A,^[^ ^15^ ^]^ where the earthworm uses peristaltic locomotion to burrow through soil, which involves a coordinated sequence of muscle contractions and relaxations along their segmented body. This allows them to move efficiently while pushing through and compacting soil. The circular muscles contract to elongate the segment, reducing its diameter, while the longitudinal muscles contract to shorten and widen the segment, increasing its diameter, as shown in Figure 3B.^[^ ^16^ ^]^ The earthworm moves forward by managing the frictional forces between its body and the surface, allowing directional movement. Similarly, directional friction is controlled between a robot and the surface to ensure smooth forward movement. Small, hair‐like structures on each segment anchor the earthworm to the ground, providing grip during movement. This mechanism excels in confined spaces and delicate environments, with innovations focusing on crawler design, smart materials, and advanced control systems for improved crawling efficiency and autonomy. Soft materials like silicone or elastomers and various actuators, such as pneumatic, hydraulic, or shape‐memory alloys, facilitate peristaltic crawling motions (see Figure 3C.^[^ ^16^ ^]^), including bending, elongation, and shortening. They are flexible, adaptable to constrained and uneven terrains, and biocompatible, making them suitable for soft crawlers. Challenges such as energy inefficiency, control complexity, and material durability persist.

When designing a soft crawler that employs a peristaltic crawling mechanism, the structure of the robot is typically organized into distinct segments. Each segment possesses the capability for independent actuation, allowing for greater flexibility and control during movement. The materials used for constructing these robots must exhibit essential properties such as elasticity, durability, and biocompatibility, as these factors significantly impact the crawler's performance and interaction with the surroundings. The control of earthworm‐like crawlers introduces a level of complexity and challenge, primarily due to the intricate timing and coordination required among the actuators. Successful replication of peristaltic motion necessitates a well‐orchestrated sequence of movements, where the actuators operate in harmony to create the desired wave‐like motion that facilitates effective locomotion. The peristaltic crawling mechanism is versatile and can be applied to soft crawlers across various sizes and scales. However, it poses a notable challenge for small‐scale soft crawlers, which may struggle to produce adequate wave‐like movements. Additionally, these smaller robots might find it difficult to generate the necessary force to achieve effective locomotion or interact meaningfully with their environment. Therefore, further research and innovation are crucial to enhance the capabilities of small‐scale soft crawlers, ensuring they can function efficiently in diverse applications.

### 2.3 Undulatory Crawling Mechanism

**Figure 4:**
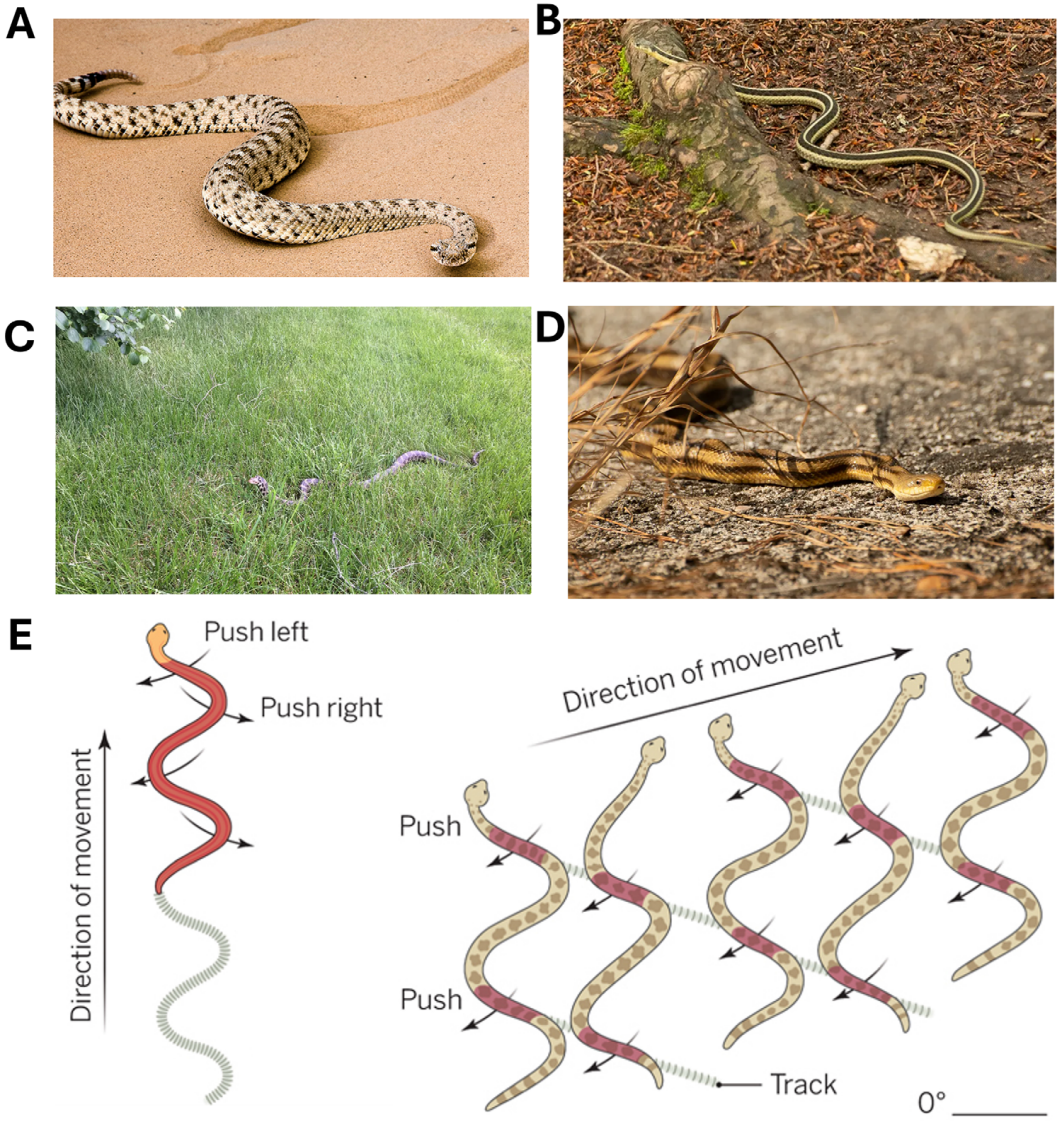
Snake crawling mechanism: A) Sidewinder rattlesnake navigating on sandy slopes. Reproduced from Socha.^[^ ^17^ ^]^ Copyright 2014, AAAS. B) Garter snake moving over leaf litter on the ground. Reproduced from Newenham.^[^ ^18^ ^]^ Copyright 2022, Exploring Nature By Sheila Newenham. C) Fox snake moving over grass. Reproduced from Newenham.^[^ ^18^ ^]^ Copyright 2022, Exploring Nature By Sheila Newenham. D) Yellow rat snake moving on a rocky surface. Reproduced from Newenham.^[^ ^18^ ^]^ Copyright 2022, Exploring Nature By Sheila Newenham. E) Snake locomotion based on lateral undulatory waves. Reproduced from Socha.^[^ ^17^ ^]^ Copyright 2014, AAAS.

Undulatory locomotion is a wave‐like movement that acts to propel an animal, such as a *snake*. Forward locomotion is generated by propagating flexural waves along the length of the snake's body. This complex mechanism relies on the interaction between the snake's muscles, its flexible body, and friction with the ground or substrate. This mechanism is more complicated than the two‐anchor and peristaltic crawling mechanisms. In most current state‐of‐the‐art soft crawlers, rigid joints are normally used to implement this mechanism. Undulation provides flexibility whenever a bend makes contact with a surface object, such as a rock or obstacle, since it exerts a force against it and deforms locally around it. For example, when a snake pushes against multiple objects simultaneously, the lateral force vectors cancel each other, leaving a resultant vector that propels the snake forward. Postural adjustment around objects gives the snake accurate control over the direction of force exertion. **Figure** **4A–D** shows the undulatory locomotion when snakes navigate on different surfaces.

Snakes produce horizontal waves that propagate from their head to their tail. The amplitude and frequency of these waves fluctuate based on environmental conditions and the speed at which the snake moves, as shown in Figure 4E. Each segment of the snake's body glides in a smooth, sinusoidal (S‐shaped) motion, creating a graceful undulation as various sections ripple from side to side. The *longitudinal muscles* along the snake's body contract sequentially, pulling and pushing different segments to maintain the wave pattern. This movement allows the snake to maintain several points of contact with the ground simultaneously, providing stability and enabling it to navigate its environment with agility and precision. *Segmental contractions* occur when the muscles in each body segment of the snake contract in a synchronized manner, creating lateral waves. The force generated by these contractions provides the necessary thrust to propel the snake forward. Snakes depend significantly on friction to move forward. They generate force by pressing specific parts of their bodies against the ground while slightly lifting other parts to reduce backward slipping. The scales on a snake's belly, called ventral scales, enhance grip by interlocking with surface irregularities. This provides traction as the snake moves against the ground with different parts of its body. To avoid slipping backward, snakes skilfully coordinate the segments of their bodies that come into contact with the ground at any moment. By precisely managing these contact points (known as “anchor” points), they prevent themselves from sliding back and enable an effective push‐off. This ability enables them to navigate the environment smoothly and efficiently, utilizing their muscle control to maintain traction and propel themselves forward. Snakes adjust the frequency and amplitude of their waves based on the terrain. For example, on smooth surfaces, they increase their wave frequency to maintain traction, while on rough surfaces, they use larger wave amplitudes, which enables them to capitalize on the friction created by the texture of the surface. Lateral undulation is the most common form of locomotion in snakes; however, they employ other methods depending on the terrain they encounter. For example, on loose or slippery surfaces like sand, where friction is low, they utilize sidewinding. In tight spaces or while climbing, snakes adopt concertina locomotion, which involves anchoring certain body segments while pushing or pulling the rest forward. Large‐bodied snakes, measuring between 2.4 and 3.96 m long, use rectilinear locomotion. This method is characterized by slower, more direct movement where their belly scales grip the ground. Soft, snake‐like crawlers have been developed to mimic undulatory movement, allowing them to navigate confined spaces and complex terrains. These crawlers are made from flexible and stretchable materials such as silicone elastomers, thermoplastic polyurethanes (TPU), shape memory alloys (SMAs), shape‐memory polymers (SMPs) and hydrogels that replicate the fluid, wave‐like movements of a snake's body. The snake‐mimic design provides the soft crawlers with a high level of adaptability, enabling movement across various terrains and the ability to enter tight spaces and traverse irregular surfaces. As a result, they are very promising for applications in medical fields, search and rescue operations, and inspection tasks.

Silicone elastomers are highly flexible, stretchable, and durable materials known for their very good elasticity, which can withstand continuous deformation without losing their shape or structural integrity. These properties make them ideal for creating wave‐like motions and enable their use in forming the outer skin or segments of soft robotic snakes, allowing the robots to flex, expand, and contract like a snake's body. TPU is known for its high resilience, exhibiting good elasticity and abrasion resistance. Its rigidity exceeds that of silicone, providing additional structural support while still allowing for some flexibility. This combination makes TPU a common choice for internal components in soft robots, as it enhances the structural stability of robots while ensuring they can be soft and flexible, moving freely. SMPs and SMAs can “remember” and return to a pre‐set shape when heated. SMAs, such as nickel‐titanium alloys, are frequently used in actuators to create bending motions that mimic muscle contractions. These SMAs are often embedded within segments of soft crawlers to function as artificial muscles, contracting and relaxing to generate undulatory waves that propel the robot forward. Hydrogels have the ability to absorb large amounts of water, enabling them to expand and contract. They are soft and flexible, with a jelly‐like consistency allowing smooth, organic movement. These properties make hydrogels particularly advantageous for underwater soft crawlers, as they can mimic the undulatory movements of aquatic snakes and other marine creatures.

### 2.4 Multi‐Legged Locomotion

**Figure 5:**
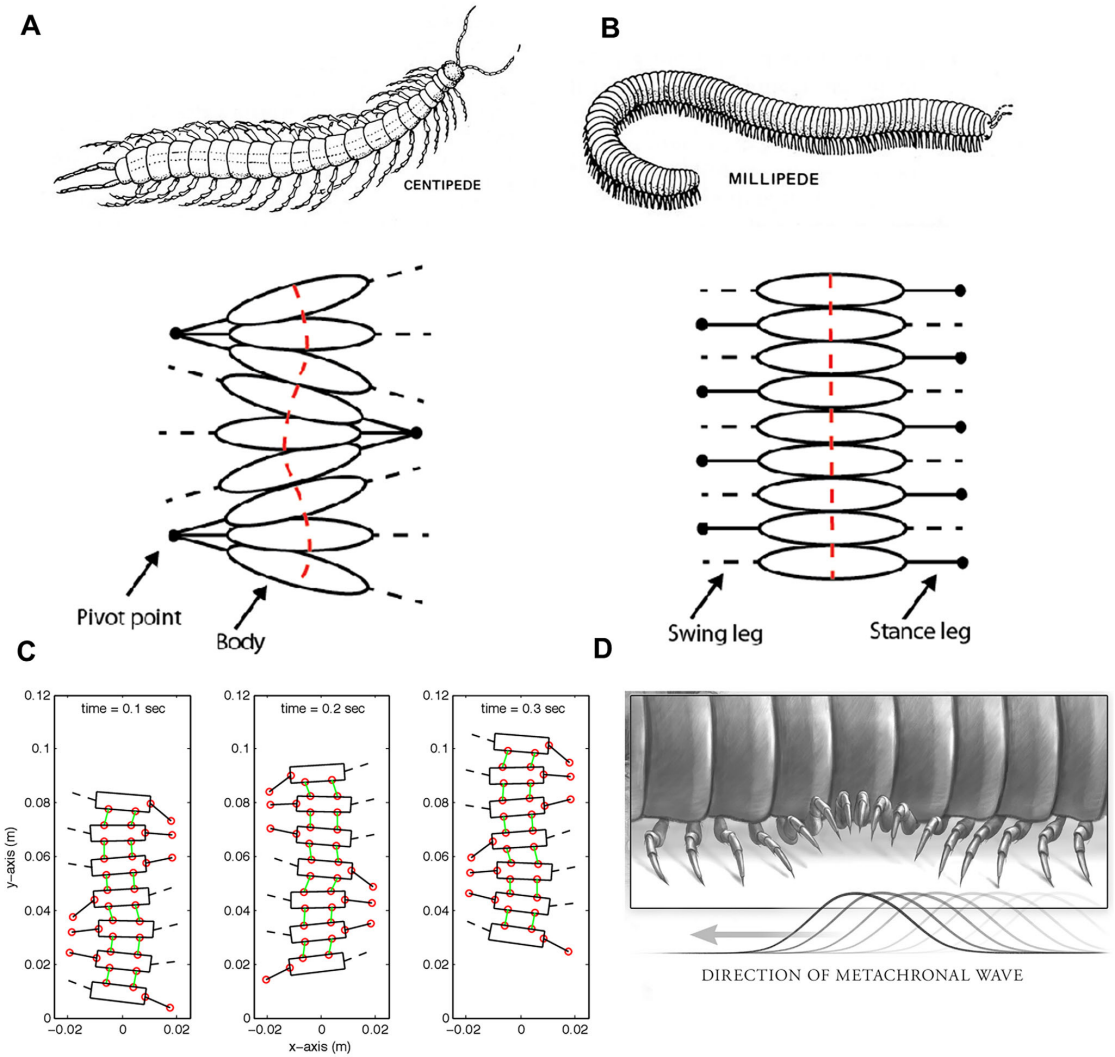
A) Centipede and B) millipede crawling mechanism. C) Schematic representation of the multi‐legged centipede locomotion with a metachronal rhythm movement pattern. D) Schematic representation of the multi‐legged millipede locomotion with a wave‐like motion. Reproduced from Kimball.^[^ ^24^ ^]^ Copyright 1978, Addison Wesley.

Multi‐legged locomotion, as observed in centipedes and millipedes, allows animals with segmented, multi‐legged bodies to move effectively across various terrains, as shown in **Figure** **5**. This legged crawling mechanism enables the crawler to maintain ground contact, stabilize movement, and distribute forces.^[^ ^19^, ^20^ ^]^ Their rhythmic, wave‐like movements with legs propel them forward efficiently. This discrete crawling strategy also enhances stability on uneven surfaces.^[^ ^21^ ^]^ This adaptability makes legged crawling particularly attractive for soft robotic designs that navigate complex environments. Multi‐legged locomotion also enhances the robotic crawler's design with flexibility and resilience. Researchers and engineers have developed segmented models with compliant materials and adaptable control systems to emulate their natural counterparts.^[^ ^22^, ^23^ ^]^ These designs enable robots to traverse diverse terrains and access environments inaccessible to traditional rigid robots, such as confined spaces and unstable or unknown surfaces.

Centipede locomotion is characterized by a coordinated motion of their legs, arranged in pairs on each body segment. This movement pattern, known as a *metachronal rhythm*, allows centipedes to efficiently propel themselves forward or backward, navigate obstacles, and adapt to various terrains, as shown in Figure 5A. Millipede legs move in a coordinated pattern of metachronal waves, creating *a wave‐like motion* and coordinating their legs sequentially to propel themselves forward or assist in burrowing. Millipedes have two pairs of legs on each body segment, and their legs move in a metachronal rhythm. This means that successive pairs of legs lift and push off the ground in waves, moving from the back to the front of the body. The movement of each pair of legs involves two stages: a posterior propulsive stroke and an anterior recovery stroke. Depending on the speed of the millipede's movement, some legs will be in the propulsive phase, while others will be in the recovery phase. This method of locomotion is *slower* than that of centipedes, which reflects millipedes' more detritivorous tendencies and less predatory lifestyle. A burrowing millipede has more legs during the propulsive phase, resulting in a slow but powerful gait with maximum forward thrust.

Inspired by centipede and millipede‐like natural organisms, robotic crawlers often use segmented bodies where each segment operates semi‐independently. This segmentation features *decentralized control*, with each leg or body element adapting to terrain changes independently and autonomously, enhancing resilience and stability.^[^ ^20^, ^25^ ^]^ For example, a control system based on Central Pattern Generators (CPGs) can synchronize each segment's movement, replicating the coordinated legged motion observed in nature.^[^ ^26^ ^]^ Many soft robotic crawlers mimic natural legged crawlers’ gait‐switching strategy to navigate diverse terrains, replicating the legged crawling gaits in centipedes. This gait‐switching strategy allows the robots to move in coordinated wave‐like patterns with legs, enhancing traction and stability. Some designs incorporate phase‐shifting controllers to adjust limb movement, adapting seamlessly to surface changes with additional discrete locomotion advantages. By mimicking this pattern, robotic crawlers can adapt their crawling style to diverse environments, transitioning between different gaits to optimize energy use and movement efficiency.^[^ ^21^, ^27^ ^]^

Discrete crawling provides robots with controlled and incremental movements. In discrete crawling, each leg or segment moves independently, allowing the robot to pause, reorient, or adjust based on environmental feedback. This stepwise movement provides several advantages, including enhanced stability, adaptive gait control, energy efficiency, and versatility in confined spaces. Discrete crawling allows for selective ground contact, enabling robots to maintain stability by anchoring specific segments or legs while others move. This makes these crawling gaits particularly beneficial for navigating uneven or unstable terrains, as demonstrated in studies on quadruped obstacle avoidance with free‐gait control.^[^ ^28^ ^]^ With each segment moving in phases, these discrete crawling gaits observed in nature inspire robots to implement phase‐shifting controllers, where individual segments adjust timing based on local terrain conditions. This dynamic adjustment includes stride length, frequency, and amplitude. Crawlers designed with these controllers show increased adaptability on varied surfaces using a steady crawl gait, thus improving performance in unpredictable environments.^[^ ^29^ ^]^ By advancing discrete steps, crawlers can further conserve energy as only a subset of actuators is engaged. This design strategy reduces continuous energy expenditure and allows energy‐efficient movement over longer durations, even across challenging landscapes.^[^ ^30^ ^]^ In addition, discrete crawling movements allow for high maneuverability in confined or narrow spaces. Unlike continuous gaits discussed in earlier sections, discrete crawling provides the benefit that each segment pivots or reorients in place, allowing for directional changes with minimal space required.^[^ ^31^ ^]^

To replicate the adaptability of natural‐legged crawlers, robotic crawlers mimicking them often employ soft, flexible actuators like shape memory alloys, dielectric elastomers, and conducting polymers.^[^ ^32^, ^33^, ^34^, ^35^, ^36^ ^]^ These materials allow robots to bend, contract, and extend in response to their environment, enabling smoothness and efficiency in not only crawling but also gripping and climbing.^[^ ^22^, ^33^ ^]^ The flexibility provided by these materials significantly contributes to performances in navigating confined spaces and transitioning between different movement modes.^[^ ^37^ ^]^ As researchers integrate advanced controllers and adaptive mechanisms, discrete crawling continues to enhance bio‐inspired robots capable of reliable, terrain‐responsive navigation.^[^ ^38^ ^]^ In many industrial settings, bio‐inspired robotic crawlers with legged locomotion have demonstrated remarkable potential. Applications include performing inspections and maintenance within tight spaces.^[^ ^22^, ^39^ ^]^ In the biomedical field, small‐scale soft crawlers with shape‐morphing capability offer promising applications in minimally invasive procedures.^[^ ^37^ ^]^ By drawing from the biomechanical principles of natural‐legged crawlers, robots have achieved great adaptability, energy efficiency, and versatility.

## 3 Actuation Strategies

A variety of actuation technologies has been effectively employed in the design and functionality of soft crawlers, including pneumatic, hydraulic, electrical, magnetic, ultraviolet (UV), and visible light, as well as chemical methods. Each of these actuation methods represents a unique role in enabling the soft crawlers to navigate through diverse environments with agility and precision. The integration of various crawling mechanisms with these actuation technologies and smart materials is critical for achieving flexible and efficient movement. For example, pneumatic actuation allows lightweight structures to adapt to different surroundings, while hydraulic systems provide robust strength for lifting and carrying objects. Electrical actuation can facilitate precise control, making it ideal for sensitive applications, whereas magnetic and light‐based methods provide non‐contact actuation options. Chemical actuation opens the door to self‐powered movement, making crawlers capable of extended operational periods. We thoroughly examine these actuation strategies and discuss their advantages and limitations in different contexts. We also identify research challenges we are facing to advance the field. We recommend suitable materials and innovative crawling mechanisms that could significantly enhance the performance and efficiency of soft crawlers, paving the way for their broader adoption in real‐world applications.

### 3.1 Pneumatic and Hydraulic Fluid Power Actuation

**Figure 6:**
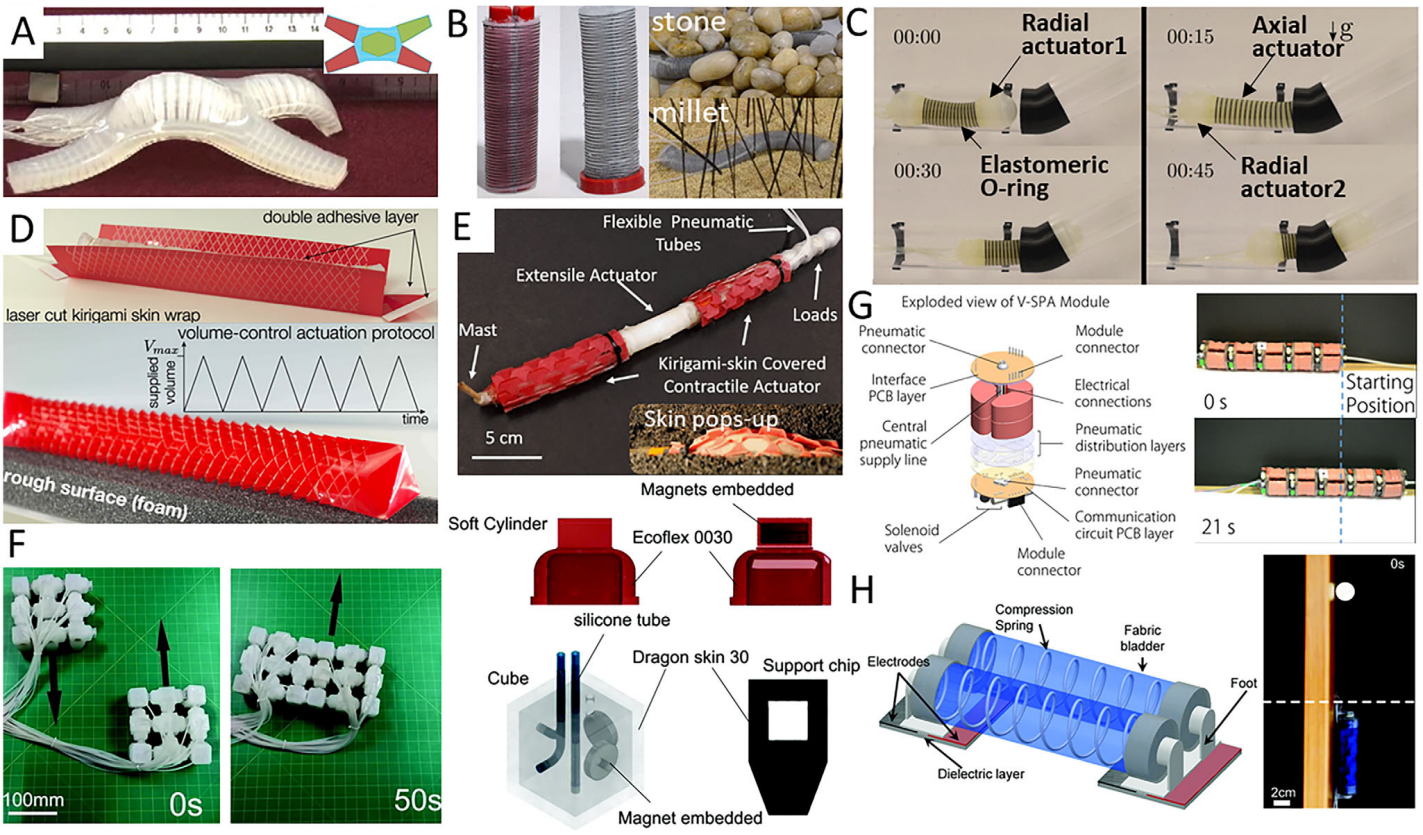
Examples of soft robots using pneumatic and hydraulic fluid power actuation. A) A multigait soft robot using a legged crawling mechanism Reproduced from Shepherd et al.^[^ ^40^ ^]^ Copyright 2011, PNAS. B) A soft snake robot using an undulatory crawling mechanism. Reproduced from Branyan and Menğüç.^[^ ^42^ ^]^ Copyright 2018, IEEE. C) A pneumatically driven soft crawler using a peristaltic crawling mechanism. Reproduced from Calderón et al.^[^ ^44^ ^]^ Copyright 2016, IEEE. D) A kirigami‐skin‐based pneumatic crawling actuator using an undulatory crawling mechanism. Reproduced from Rafsanjani et al.^[^ ^45^ ^]^ Copyright 2018, AAAS. E) A pneumatic soft earthworm robot using the trapezoidal patterned kirigami skin and the peristaltic crawling mechanism Reproduced from Liu et al.^[^ ^48^ ^]^ Copyright 2019, IEEE. F) A pneumatically driven, reconfigurable omnidirectional robot Inspired by the caterpillar locomotion mechanism. Reproduced from Zou et al.^[^ ^50^ ^]^ Copyright 2018, Mary Ann Liebert. G) A vacuum‐powered soft pneumatic actuator designed for creating complex soft robotic systems with multiple degrees of freedom. Reproduced from Robertson et al.^[^ ^52^ ^]^ Copyright 2017, AAAS. H) A soft crawler with two vacuum‐powered spring actuators and two electrostatic actuators. Reproduced from Qin et al.^[^ ^53^ ^]^ Copyright 2019, Mary Ann Liebert.

Pneumatic actuation is one of the widely used mechanisms to drive soft robots. Soft pneumatic‐driven actuators normally consist of designed channels that enable the compressed air to inflate the channel and generate the desired deformation for the locomotion of soft robots. The multigait soft robot developed by Shepherd et al. in 2011 is one of the classic examples using the legged crawling mechanism,^[^ ^40^ ^]^ as shown in **Figure** **6**A. The Pneu‐Net architecture^[^ ^40^ ^]^ is used to design the crawler, and the operating pressure of the PN is about 0.7 bar. The length of the robot is about 14.6 cm. This quadrupedal robot is made of soft elastomeric polymers and manufactured using soft lithography. Ecoflex 00–50 Smooth‐On Inc. was selected as the actuating layer due to its high extensibility under low‐stress conditions. Meanwhile, PDMS was chosen as the strain‐limiting layer owing to the relatively inextensible nature under the stresses induced during the pressurization of the pneumatic networks (PNs). A pneumatic valve‐controlled system is used to drive five combined soft actuators to realize legged crawling locomotion, allowing the robot to crawl at about 0.027 m s^−1^. Using a very similar design but with a modified Pneu‐Net architecture, an enhanced untethered soft crawler is developed by Tolley et al.^[^ ^41^ ^]^ The robot can carry a load of up to 8 kg. Given the considerable size of the robot, measuring 0.65 m in length, molds constructed from laser‐cut 6 mm thick acrylic sheets were chosen as a cost‐effective and flexible fabrication approach. The addition of integrated alignment features allowed for the precise assembly of the cut acrylic pieces into a three‐dimensional mold. The modular design of these molds greatly facilitated the demolding process of the cast robots. To improve durability, a water‐jet system was employed to cut aluminum for the thinnest mold components, which were particularly susceptible to fracture during demolding. The crawler was demonstrated under a variety of adverse environmental conditions, including snow, puddles of water, (albeit limited) exposure to flames, and the crushing force of being run over by an automobile. The results show its crawling effectiveness, robustness, and resilience. Using the legged crawling mechanism, the crawlers can perform complex motion using a sequence pneumatic actuation mechanism, but usually with a slow crawling speed. Using the undulatory crawling mechanism, Branyan et al. developed a soft snake robot,^[^ ^42^ ^]^ which can navigate through a variety of granular terrain, including sand, millet, and stone, using different traveling waves. The robot comprises two fiber‐reinforced actuators in series, as shown in Figure 6B. An inextensible backbone was integrated to prevent extension and encourage curvature. The addition of this inextensible material, placed between the chambers, altered the fabrication process. Proper placement and strong adhesion to the silicone elastomer EcoFlex 00–30 were essential for embedding the backbone material. Each actuator has two chambers for bidirectional bending and an elliptical cross‐section to prevent rolling. The robot is 20 cm × 3 cm × 2 cm and weighs 70g. The maximum crawling velocity of the robot is 2.2 mm s^−1^ (0.011 body‐length s^−1^) on the paper surface. Zhang et al. applied three fiber‐reinforced actuators to further enhance the locomotion ability of the robots,^[^ ^43^ ^]^ which has been demonstrated under a variety of complicated environments, including dry, oil, and liquid‐filled tubes in circular, square, triangle, and D shapes, and underwater environment. In the fabrication process, the retractable combined casting (RCC) method was developed to enable the casting of the complex structure of the robot through structured segmentation and a controlled casting sequence. The robot can carry a load of 157.3 g, which is more than 10 times its weight (14.5 g). The crawler also has a visualization unit and electromagnetic sensor for real‐time visual tracking.

Applying the peristaltic crawling mechanism, a pneumatically driven soft crawler was developed by Calderón et al., which can mimic the motions and replicate the functionality of a single burrowing earthworm segment,^[^ ^44^ ^]^ as shown in Figure 6C. The fabrication method utilizes 3D‐printed acrylonitrile butadiene styrene (ABS) molds, silicone elastomer Ecoflex 00–50, butadiene rubber elastomeric O‐rings, fiberglass sheets, and pneumatic components as physical inputs. The crawler is 130 mm in length and 35 mm in diameter and consists of three actuators: a rear radial actuator, a central axial actuator, and a frontal radial actuator. The two radial actuators are analogous to the worm's circular muscles, and the axial actuator is analogous to the longitudinal muscles. The robot is capable of locomoting horizontally, vertically, and inside a varying‐slope transparent pipe, with an average speed of about 12 mm s^−1^. A feedback control system is designed based on stretchable liquid circuits, which are capable of measuring strain and pressure.^[^ ^15^ ^]^ Using the undulatory crawling mechanism, Rafsanjani et al. designed a kirigami‐skin‐based pneumatic crawling actuator.^[^ ^45^ ^]^ Unlike actuating several actuators independently to enable the robot to crawl, the kirigami‐skin robot crawls for the first time by using a highly stretchable 3D‐textured kirigami skin, which is inspired by the skin of a snake, as shown in Figure 6D. The buckling‐induced directional frictional properties of the kirigami surfaces of the robot enabled the system to crawl efficiently.^[^ ^45^, ^46^ ^]^ The crawling speed varies with four different cutting textures, including linear cuts, triangular cuts, circular cuts, and trapezoidal cuts. It has been found that the trapezoidal skin is more stretchable than those with circular and triangular cuts, enabling the crawler to have a longer stride and move further. The proposed kirigami approach provides a simpler, faster, and more cost‐effective technique for fabrication. The efficiency of kirigami‐skinned crawlers can be enhanced by optimizing the frictional properties and stretchability of the skins through careful selection of cut geometry and actuation protocols. Additionally, while plastic deformation at the hinges can be leveraged to further refine system performance, the reversible and repeatable deformation process within the elastic regime enables controlled and adjustable friction, which is valuable for applications such as robotic manipulation and transfer printing.

The soft snake‐like robot, utilizing a kirigami skin and an undulatory crawling mechanism, comprises two pneumatic actuators capable of crawling along a curvature path.^[^ ^47^ ^]^ Using the trapezoidal patterned kirigami skin and the peristaltic crawling mechanism, a pneumatic soft earthworm robot was developed to crawl under cohesive soil.^[^ ^48^ ^]^ To maximize the performance of kirigami skin deformation features for anchoring while preventing structural failure, a new fabrication method was developed to enhance the robustness of the kirigami skin by integrating a single polyester plastic sheet with a silicone membrane. The plastic side of the membrane is positioned inward, while the silicone side faces outward to increase the interaction force with the environment. The robustness of the kirigami skin is significantly enhanced by integrating a plastic sheet layer with a silicone layer. The stretching ability of the silicone‐plastic kirigami skin was evaluated, and the results show that under an applied strain of up to 96%, some plastic segments of the silicone‐plastic kirigami may break. However, the silicone layer maintains the structural connection. At this strain level, the stretching force per unit width is ≈0.045 N cm^−1^, allowing the remaining structure to deform effectively while maintaining its functional shape. This robot consists of three pneumatic actuating segments: head and tail segments that expand radially as anchors and a middle segment that elongates the body (see Figure 6E). The silicone‐plastic kirigami skin pops up when the actuator is radially expanded, forming bristle‐like spikes that are perpendicular to the surface and fold back down when the actuator is deflated, forming a smoother skin structure. This structure enables the robot to effectively crawl under cohesive soil. The robot exhibits a maximum drag force of about 6 N when crawling in a soil channel with a soil density of 0.446 g cm^−3^ and a diameter of 25 mm. Xie et al. created a pneumatic soft robot, PISRob, using the two‐anchor crawling mechanism for crawling locomotion.^[^ ^49^ ^]^ The materials used in PISRob are common and cost‐effective, including Ecoflex 00–30, glass fiber, Kevlar fiber net, O‐rings, silicone air tubes, and transparent adhesive tape. The robot has a soft body and two soft legs combined in an H‐shaped. When actuated, the main body and legs can bend to an Ω‐shape. A pneumatic feedforward valve‐controlled system is designed for the crawler to provide the control sequence. The crawler can move about 30 mm (14% of its body length) with one crawling cycle at a speed of 6.7 mm s^−1^.

Soft reconfigurable omnidirectional robots have been developed for their multitasking capability, versatility, and dexterity. They usually consist of a series of segment pneumatic actuators that can perform a variety of motions, including walking, turning, twisting, and bending. Inspired by the caterpillar locomotion mechanism, a pneumatically driven, reconfigurable omnidirectional robot was developed by Zou et al.,^[^ ^50^ ^]^ as shown in Figure 6F. To realize omnidirectionality, nine actuator modules were arranged as a 3 × 3 matrix to form a soft robot body. The length of the body is 154 mm. The robot can travel at a speed of about 18.5 m h^−1^ (two body lengths per minute), and it can rotate at a speed of 1.63 degree s^−1^. The actuator modules were embedded with neodymium‐iron‐boron (NdFeB) magnets, which can be easily removed and combined to create different robot configurations. This provides the opportunity to use soft omnidirectional robots to explore and inspect unstructured environments. The robots are tethered, and the modules are controlled separately using feedforward control systems. Intelligent inter‐communicated feedback control systems and possible untethered solutions could enhance the capability of the robots.

Wang et al. designed a pneumatic module‐based soft crawler which consists of three pneumatic soft modules in series.^[^ ^51^ ^]^ Each module has two individual soft actuators arranged in parallel. The two‐anchor crawling mechanism was applied to control the locomotion. With sequential controlling signals, the crawler can realize bending, crawling, and steering motions. Using a single module as a crawler in experiments, it can achieve a crawling speed of 9.75 mm s^−1^ and a steering speed of 1.58 degree s^−1^, while the module‐based crawler can achieve a crawling speed of 4.81 mm s^−1^ and steering speed of 0.71 degree s^−1^.

Pneumatic vacuum‐powered actuators have also been employed to actuate soft robots. Different from conventional pneumatic actuation systems, the vacuum‐powered actuation technology *deflates* the air channel to generate the desired locomotion. A vacuum‐powered soft pneumatic actuator that can be used to form complex soft robotic systems with multiple degrees of freedom (DoFs) was developed by Robertson et al.,^[^ ^52^ ^]^ as shown in Figure 6G. This design features the V‐SPA, which comprises a laser‐cut, robust foam core with thin layers of brushed‐on silicone rubber and can be manufactured from scratch in less than two hours. The plug‐and‐play module/robot, which is built by using five vacuum‐powered soft pneumatic actuators, was demonstrated. The average crawling speed is 5 mm s^−1^ on a flat and smooth surface. To enhance the capability of the robots, the team also used granular jamming for varying robot stiffness and the direct suction adhesion system for vertical window climbing. The climbing speed of the robot is about 2 mm s^−1^ (0.01 body length s^−1^) with a feedforward open‐loop control. The vacuum‐powered robot is able to carry a load of up to 70 g. This work enhances the efficiency of soft robotic systems by reducing actuator mass, allowing the integration of onboard components for fully self‐contained operation. The proposed design simplifies fabrication by eliminating the need for internal or external molds, enabling rapid production in under two hours. Additionally, the lightweight and low‐cost nature of the actuator facilitates the development of larger, more complex soft robotic systems with increased degrees of freedom.

Qin et al. designed a soft crawler that consists of two vacuum‐powered spring actuators and two electrostatic actuators.^[^ ^53^ ^]^ The vacuum‐powered spring actuators enable a large deformation of the robot, and the electrostatic actuators, which are used as robot feet, enhance the adhesion between the robot and the surface, as shown in Figure 6H. Each actuator incorporates a compression spring enclosed within an airtight bladder made of thermoplastic polyurethane‐coated polyester fabric, with a thin silicone tube connecting the bladder to a vacuum pump for pneumatic actuation. The electrostatic pad comprises two parallel compliant electrodes made of silver nanowire and a dielectric layer of polyurethane to generate an electric field, allowing to generate electro adhesion force between the actuator and the dielectric surface. The combination of these two technologies enables a rapid crawling motion and turning speed of the robots. The length of the crawler is 135 mm. Driven by the pneumatic power, the crawler can effectively navigate confined spaces and climb a vertical wooden wall. The maximum speed the soft crawler achieved with optimized actuation is around 16.29 mm s^−1^ (0.12 body length s^−1^), which is comparable to other vacuum‐powered crawling robots (0.08 body length s^−1^)^[^ ^52^ ^]^ and pneumatic actuators (0.2 body length s^−1^).^[^ ^40^ ^]^ The climbing speed of the robot is 6.67 mm s^−1^ (0.049 body length per second), and the crawling speed is 9.71 mm s^−1^ (0.0713 body length s^−1^) with a payload of 23 times of its self‐weight. The robot can carry a maximum load of 69 times its weight on a horizontal surface. The crawler can achieve a very high turning speed of 15.09 degree s^−1^.

Hydraulic actuation uses liquid to drive soft robots, which has a similar working principle to pneumatic‐driven systems, so both pneumatic and hydraulic actuators are categorized as fluid‐powered actuating systems. Hydraulic actuation provides the advantages of fast and robust response, high‐power density, and ease of control due to relatively incompressible liquids, which has potential in the field of soft robots. The hydraulic power can be transferred almost immediately from the power source to the actuators. However, higher pressures are needed in the system to achieve certain volumetric flow rates, and the hydraulic systems can be much stiffer than pneumatic‐driven systems. One of the classic examples is the hydraulic fish developed by Katzschmann et al.^[^ ^54^ ^]^ Water was used as a transmission fluid to actuate the fish body, and a closed‐loop feedback control system was designed to perform accurate motion control. The fish is 0.45 m × 0.19 m × 0.13 m in size. In fish tank experiments, the fish swam in a tank of a length of 1.22 m in a straight, horizontal line from wall to wall and repeated 25 times. The results show that the horizontal swimming speed was 0.10 m s^−1^, which is equivalent to 0.15 body lengths per second. The autonomous soft‐bodied robotic fish is capable of sustained swimming in three dimensions. This work proved that fish‐like locomotion could be effective for the navigation of underwater robots. A comprehensive review of fish swimming locomotion can be found in the reference,^[^ ^55^, ^56^ ^]^ and the applications of underwater robots can be found in the reference.^[^ ^57^ ^]^ MacCurdy et al. fabricated a hydraulically driven soft robot using multimaterial 3D printing technology that allows them to print both rigid and liquid components of the robots.^[^ ^58^ ^]^

Printable hydraulics enables the simultaneous deposition of force‐transmitting fluid and solid structures, removing the need for additional assembly and allowing the creation of complex actuated designs. The approach supports intricate geometries that are infeasible with other 3D printing methods while simplifying control through the use of an incompressible working fluid. Non‐curing liquids act as removable support structures, and the integration of solid and fluid regions removes the need for air bubble purging. Compared to kinematic linkages or gears in active 3D‐printed assemblies, printable hydraulics offers low friction, low backlash, and high force‐transmission elements. A hexapod robot that mimics a tripod gait with six rotational DOFs was fabricated. A fluidic channel is printed into the body, which enables the backward and forward motions of the robot, which can achieve a speed of 1.75 cm s^−1^ (equivalent to 0.125 body length s^−1^). The legged walking mechanism is used to enable the hexapod robot to walk. Zhao et al.^[^ ^59^ ^]^ developed a miniaturized soft crawling caterpillar, which was actuated by an Electrohydrodynamic (EHD) pump to provide the driving force. The top, channel, electrode, and bottom layers of the EHD pump were composed of polymer film (130 µm thick, DongMei Material Technology), silicon film (800 µm thick, Dudao United Chemical Company), Cu sheet (20 µm thick, Kunshan Shengshi Jingxin New Material Company), and precast PDMS film (1000 µm thick, Dow Corning), respectively. All layers were patterned using ultraviolet (UV) laser‐cutting technology. Assembled with the stretchable artificial muscle responsible for executing the crawling motion and a square reservoir filled with dielectric fluorinated liquid, the crawling motion is executed, and the process is enhanced. The crawling velocities were 0.73, 0.63, 0.52, and 0.40 mm s^−1^, respectively, on diverse terrains, including smooth surfaces, a 10° inclined plane, natural crystal river sand (2–3 mm in diameter), black rainforest sand (1 mm in diameter), and Nile sand (0.2–0.5 mm in diameter). As a result, the robustness and versatility of the soft crawling caterpillar was demonstrated. The spider‐inspired underwater crawling robot designed by Tan et al.^[^ ^60^ ^]^ proposed a rigid–soft hybrid multi‐joint leg design with quasi‐linear motion range and force exertion to maintain passive impact compliance. In this design, a novel valve‐free hydraulic actuation system was developed using peristaltic pumps, creating a compact, lightweight, and untethered underwater crawling robot prototype. This design achieved a remarkable 5:1 payload‐to‐weight ratio and demonstrated multi‐gait capabilities, highlighting its potential for advanced underwater applications. In general, hydraulic‐driven soft crawlers usually present more complex designs and structures than pneumatic‐driven crawlers. The locomotion mechanisms used for hydraulic soft crawlers are also more complicated than the completely soft robots due to the nature of the hydraulic actuation that provides faster and stiffer responses than the pneumatic‐driven counterpart.

As shown in **Tables** **1** and **2**, pneumatic and hydraulic fluid power actuations have played a crucial role in enhancing the capabilities of soft robotic crawlers. These actuation methods utilize the fundamental principles of fluid dynamics to generate controlled movement, making them particularly suitable for applications that require flexibility, force output, and adaptability to diverse environments. While pneumatic actuators primarily use compressed air to achieve motion, hydraulic actuators employ less compressible fluids to provide greater force and precision. The choice between these two approaches depends on the specific requirements of the robotic system, balancing factors such as power density, response speed, compliance, and control complexity.

**Table 1:** Summary of the pneumatic and hydraulic soft crawling robots in the reviewed studies.

Reference	Fluid Power	Mechanism	Design			Locomotion and Speed	Key Features
			Size	Weight	Load Capacity		
Shepherd et al.^[^ ^40^ ^]^	pneumatic	legged crawling mechanism	125 mm body length	/	8 kg	undulating:13 ± 0.6 m h^−1^ crawling: 24 ± 3 m h^−1^ crawling with slightly stiffer elastomer (Ecoflex 00–50; Smooth‐On Inc.): 92 ± 4.3 m h^−1^	Quadrupedal Each leg can be controlled independently; lightweight Essentially inviscid and thus allows rapid motion No sensor used
Tolley et al.^[^ ^41^ ^]^	pneumatic	legged crawling mechanism	650 mm body length	2.1 kg	500 kg	walking: 18.0 m h^−1^ (minimum) undulating: 2.0 m h^−1^ (minimum)	Modified Pneu‐Net architecture Capable of operating at elevated pressures (up to 138 kPa) Demonstrated under a variety of adverse environmental conditions Turning gait developed
Branyan et al.^[^ ^42^ ^]^	pneumatic	undulatory crawling mechanism	20 cm (L)× 3 cm (W)× 2 cm (H)	70 g	/	crawling: 2.2 mm s^−1^ (maximum)	Snake‐like robots Can navigate through various granular terrain with different travelling waves.
Zhang et al.^[^ ^43^ ^]^	pneumatic		/	14.5 g	157.3 g		Demonstrated under a variety of complicated environments Equipped with a visualization unit and electromagnetic sensor for real‐time visual tracking
Calderón et al.^[^ ^44^ ^]^	pneumatic	peristaltic crawling mechanism	130 mm (L) ×35 mm (D)	/	/	locomoting horizontally, vertically and inside a varying‐slope transparent pipe with an average speed of 12 mm s^−1^.	Earthworm‐inspired Novel multi‐casting‐based fabrication method developed A multi‐material multi‐actuator created
Rafsanjani et al.^[^ ^45^ ^]^	pneumatic	undulatory crawling mechanism	164 mm body length and 25 mm triangular cross‐section with edges	/	/	/	Kirigami principles inspired. 3D‐textured snake‐like surface with directional frictional properties Allow locomotion even with a single extending actuator A simpler, faster, and cheaper technique to create them in manufacture
Branyan et al.^[^ ^47^ ^]^	pneumatic	undulatory crawling mechanism	/	/	/	crawling: 6 mm s^−1^ (maximum) with triangular scale profile with micro‐ornamentation	Snake‐inspired skin based on kirigami techniques attached to a snake‐like robot An increase of 335% over the robot with no skin when activated to the maximum achievable curvature
Liu et al.^[^ ^48^ ^]^	pneumatic	undulatory crawling mechanism	65 mm (L) × 20 mm (D) with a thickness of 1.5 mm	/	40 g	crawling speed varies with four different cutting textures, maximum drag force: 6 N	Wormlike robot combining Kirigami skin with radially expanding pneumatic actuators Bristle‐like spikes modelled by Kirigami structure and formed by the actuator is radially expanded The Kirigami skin‐covered robot exhibits a greater maximum drag force, greater forward displacement, and higher traction
Xie et al.^[^ ^49^ ^]^	pneumatic	two‐anchor crawling mechanism	213 mm body length	/	/	Crawling: 6.7 mm s^−1^ Climbing: 10 mm s^−1^	A soft body and two soft legs combined in an H‐shaped The main body and legs can bend to a Ω‐shape during actuation Two end parts as legs can conduct simple bending motion for grasping or anchoring
Zou et al.^[^ ^50^ ^]^	pneumatic	undulatory crawling mechanism	length of the body is 154 mm	/	/	Crawling: 18.5 m h^−1^ Rotating: 1.63 degree s^−1^.	Neodymium‐iron‐boron (NdFeB) magnets embedded in the modules Allow easily replaced or combined into other configurations
Wang et al.^[^ ^51^ ^]^	pneumatic	two‐anchor crawling mechanism	single module: 113 mm body length module‐based crawler: 339 mm (L) × 70 mm (W)	/	/	Single module: crawling: 9.75 mm s^−1^, turning: 1.58 degree s^−1^, Module‐based crawler: crawling: 4.81 mm s^−1^ turning: 0.71 degree s^−1^	Modular differential drive soft robot (MSSDR) consists of different drive soft modules (DDSMs) Achieve multiple degrees of freedom by connecting modules
Robertson et al.^[^ ^52^ ^]^	pneumatic	vacuum‐powered pneumatic mechanism	each module has a height of 45 mm with a diameter of 45mm 200 mm length of body	45 g	70 g	crawling: 5 mm s^−1^ with 5 modules, 11 mm s^−1^ with 3 modules on a flat and smooth surface Rolling: 60mm s^−1^ with 4 modules Climbing: 2 mm s^−1^ with 2 modules	Plug‐and‐play V‐SPA module Achieve 3‐DoF A single, shared vacuum power supply reducing subsystems compared to a single source of pneumatic power Fabricated from simple materials and methods, allowing rapid batch production with minimal effort
Qin et al.^[^ ^53^ ^]^	pneumatic	vacuum‐powered and electrostatic mechanism	135 mm body length	43 g	3000 g	Crawling: 16.29mm s^−1^ (maximum) climbing: 6.67 mm s^−1^ turning: 15.09 degree s^−1^	Consists of two vacuum‐actuated spring actuators and two electrostatic actuators Rapid crawling motion and turning speed. Can move in inclined even vertical surfaces. Can work well in confined spaces with undesirable obstacles. Can function in more complex environments with gaps
Katzschmann et al.^[^ ^54^ ^]^	hydraulic	undulatory swimming mechanism	0.45 m (L)× 0.19 m (W)× 0.13 m (H)	1.65 kg	/	swimming: 0.10 m s^−1^ diving: 0.015 m s^−1^	Fish‐like robot Swimming in three dimensions New closed‐circuit drive system using water as transmission fluid Underwater operation
MacCurdy et al.^[^ ^58^ ^]^	hydraulic	legged crawling mechanism	14 cm (L)× 9 cm (W)× 7 cm (H)	690 g	/	crawling: 1.75 cm s^−1^	Tripod‐gait hexapod robot Six rotational DOFs Fabricated by 3D printers and no assembly required
Zhao et al.^[^ ^59^ ^]^	hydraulic	undulatory crawling mechanism	109.5 mm (L) ×21.5 (W)× 20 (H)	/	/	crawling: 0.73 mm s^−1^ (maximum)	Equipped EHD pumps Obtain a maximum bending angle of 52° Simple fabrication
Tan et al.^[^ ^60^ ^]^	hydraulic	legged crawling mechanism	625 mm(L) × 400 mm(W)	5.75 kg	300 N	/	Spider‐inspired robot A rigid–soft hybrid multi‐joint leg design Valve‐free hydraulic actuation system

**Table 2:** Summary of the advantages and disadvantages of the pneumatic and hydraulic actuation power in the reviewed studies.

Fluid power	Advantage	Disadvantage
Pneumatic	Lightweight structure Simple Resist overheating and withstand wet and moisture‐ridden environments Quicker response	Less efficient due to pressure loss and air compressibility Highly customized actuator for merely one application Large power supply system required
Hydraulic	Robust strength More complex designs and structures Can hold a constant force without the pump supplying more fluid	May lead to cleanliness issues Accompanying components required High maintenance systems

Pneumatic actuators are widely used in soft robotics for their compliance and ability to achieve significant deformations with simple control systems. They consist of elastomeric chambers or soft artificial muscles that expand or contract with air pressure. Their compliant nature allows safe human interaction, leading to the creation of soft crawlers. Pneumatic actuation also enables variable stiffness control; adjusting air pressure alters rigidity, allowing robots to shift between flexible and stiff states. However, challenges like control precision, energy efficiency, and bulky compressors limit their use in mobile soft robotic systems. In contrast, hydraulic actuators use less compressible fluids for motion, providing superior force output and precision, which is ideal for heavy‐duty applications such as underwater robotics and bioinspired locomotion. Hydraulic actuation maintains consistent force output regardless of size, making it reliable for demanding tasks. Hydraulic systems have demonstrated exceptional utility in submersible soft robots, where the use of fluids mitigates buoyancy‐related fluctuations in actuation performance. Researchers have harnessed hydraulics to develop bioinspired underwater robots that emulate the movements of marine organisms such as octopuses, jellyfish, and fish, facilitating efficient locomotion in aquatic environments. However, hydraulic systems tend to be heavier, more complex, and susceptible to leakage, necessitating precise sealing and regular maintenance. Energy efficiency can pose a concern, particularly in portable or autonomous crawling systems, where limitations in power supply restrict the long‐term operation of hydraulic‐driven robots.

Pneumatic and hydraulic fluid power actuation in soft crawlers use highly flexible and compliant materials, including silicone rubbers, polyurethanes, hydrogels, and shape‐memory polymers (SMPs). These materials are essential due to their elasticity, durability, and adaptability, which facilitate controlled deformation and effective force transmission. Reinforcement strategies, such as the incorporation of embedded fibers like Kevlar and the utilization of layered composites, are instrumental in optimizing directional stiffness while mitigating undesired expansions. The selection and integration of these materials significantly enhance the efficiency, durability, and adaptability of soft crawlers and robots across a variety of domains, including biomedical applications, industrial automation, and exploratory systems. Fabrication techniques are crucial in ensuring precision and optimal performance in soft crawlers. Traditional mold casting continues to be a prevalent method for producing elastomer‐based actuators. Advanced methodologies such as 3D printing facilitate the creation of intricate geometries and embedded channel structures. Additionally, layered assembly techniques with embedded reinforcements can enhance mechanical control, improving the actuation precision of the crawlers. These fabrication advancements contribute to superior performance by enabling fine‐tuned motion control, durability, and adaptive morphing capabilities. As a result, fluid‐powered soft robots are becoming more efficient, robust, and versatile, finding increasing applications in human‐robot interaction, medical devices, and dynamic adaptive systems.

### 3.2 Electric Field Actuation

Electric fields or currents are widely used to achieve actuation and drive active functional materials, such as dielectric elastomers, shape memory alloys (SMAs), electroactive polymers, and piezoelectric materials. These materials can provide large actuation strain, high compliance, and energy density for developing soft crawlers. Dielectric elastomer‐based crawling robots have been developed to improve the speed of motion and increase the degree of freedom for locomotion.

#### 3.2.1 Dielectric Elastomers

**Figure 7:**
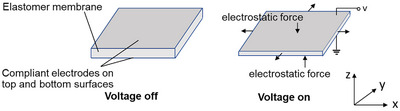
Working mechanism and deformation of dielectric elastomers actuator. Reproduced from Pelrine et al.^[^ ^65^ ^]^ Copyright 2000, AAAS.

Dielectric elastomer actuators (DEAs) are a class of electroactive polymers that operate based on deducing deformation with an electric field. They belong to the group of electroactive polymers (EAP). EAPs have become highly promising materials for soft crawlers due to their ability to undergo significant deformations when exposed to an electric field, mimicking the flexible and adaptive movements found in nature.^[^ ^61^ ^]^ EAPs are lightweight, flexible, and capable of producing large strains, which are critical for enabling smooth, continuous crawling motions in soft robotic systems.^[^ ^62^ ^]^ Based on their actuation mechanisms, EAPs are mainly classified into two types: ionic EAPs and electronic EAPs. Ionic EAPs work through the movement of ions within the polymer when an electric field is applied. These materials typically operate at low voltages (under 10 V) and generate large deformations but tend to have slower response times due to ion migration.^[^ ^63^ ^]^ Electronic EAPs, on the other hand, use electrostatic forces to induce mechanical deformation and require high voltages (often in the kilovolt range). While they offer faster response times compared to ionic EAPs, they also require high voltages and must be pre‐strained.^[^ ^64^ ^]^ Although these materials present significant opportunities for the development of soft robotics, challenges remain in terms of energy efficiency, response time, material fatigue, durability, scalability, and cost‐effectiveness. Current efforts to enhance material properties often improve one or two aspects, but it remains challenging to optimize all these factors simultaneously. DEAs are able to respond to electrical stimuli to generate large deformations and/or shape changes. A classic DEA comprises an insulating elastomer membrane sandwiched between two compliant electrodes. When a voltage is applied between the electrodes, the rising electric field causes a decrease in thickness and an increase in the area of the membrane,^[^ ^65^ ^]^ as shown in **Figure** **7**. This process effectively transforms electric energy into mechanical work and provides large strain. The DAE actuation principle was first demonstrated by Wilhelm Conrad Röntgen in 1880 by spraying charges on a piece of natural rubber.^[^ ^66^ ^]^

Dielectric elastomers can generate large strains when exposed to electric fields, which simulate the contraction and expansion required for the anchor‐release motion seen in two‐anchor crawling. In 2000, Pelrine et al. published their findings in Science,^[^ ^65^ ^]^ demonstrating that dielectric elastomers can convert electrical energy into mechanical motion. When voltage is applied, electrostatic forces compress the dielectric elastomer films in thickness, expand them in area, and produce strain. The materials used for dielectric elastomers generally include acrylic, polyurethane, and silicone.^[^ ^67^ ^]^ Some research tried to improve the response speed of acrylic polymers, such as optimizing the cross‐linking network^[^ ^68^ ^]^ and applying plasticizers.^[^ ^69^ ^]^ Compared to acrylic, silicones typically offer a faster response time but may generate lower strains and have reduced energy density. Commercially available polyurethane dielectric elastomers may be less appealing due to their high moduli, which may generate smaller actuation strains.^[^ ^70^ ^]^ DEAs are compliant capacitors that are lightweight and have a high elastic energy density, making them one of the emerging actuating technologies in soft robotics. DEAs have been developed in various configurations, including planar, stack, bimorph and unimorph, diaphragm, bulged membrane, and spring roll, to suit different applications in soft robotics.^[^ ^71^ ^]^ The first DEA‐based walking robot (FLEX) was proposed and validated in,^[^ ^72^ ^]^ though its walking speed was limited to only a few centimeters per hour. Since the successful demonstration of DEA technology in walking robots, extensive research has been conducted to improve movement speed and increase degrees of freedom (DOFs) for more advanced locomotion.

**Figure 8:**
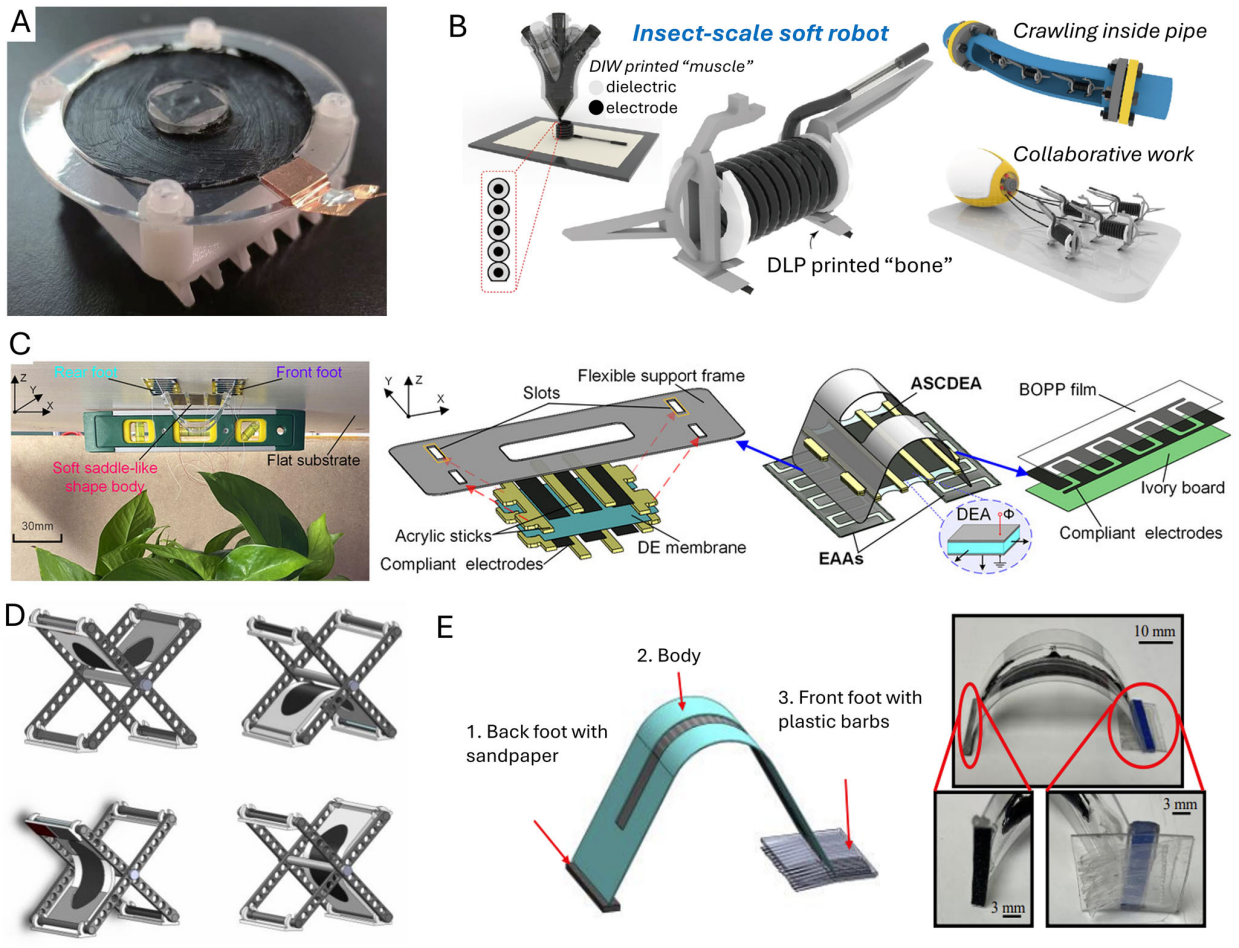
Crawling robots actuated by dielectric elastomers: A) A crawling robot driven by a single‐layer cone‐shaped DEA. Reproduced from Xue et al.^[^ ^74^ ^]^ Copyright 2022, IEEE. B) An insect‐scale crawling robot driven by coiled DEAs. Reproduced from Zhu et al.^[^ ^75^ ^]^ Copyright 2023, ACS Publications. C) A DEA‐actuated multimodal locomotion‐based soft robot. Reproduced from Hu et al.^[^ ^76^ ^]^ Copyright 2023, John Wiley & Sons. D) A DEA‐driven crawling robot using a 3D scissor mechanism and electrostatic adhesion. Reproduced from Zhong et al.^[^ ^77^ ^]^ Copyright 2024, IEEE. E) A DEA bending module‐based soft‐moving robot. Reproduced from Song et al.^[^ ^78^ ^]^ Copyright 2024, ASME.

Du et al. (2022) developed a crawling robot actuated by a planar DEA, capable of generating low‐profile vibrations to induce forward crawling motion. The proposed robot weighed 2.6 g and had a load capacity of 4.9 g. Its crawling speed reached 169.2 mm s^−1^ at a peak driving voltage of 6 kV. However, the robot is unable to effectively control its movement direction due to its reliance on a single degree‐of‐freedom actuator.^[^ ^73^ ^]^ Xue et al.^[^ ^74^ ^]^ presented a crawling robot driven by a single‐layer cone‐shaped DEA (SCDCR), as shown in **Figure** **8**A. The robot uses a single‐layer conical DEA with a deadweight, which is simpler and more compact compared to the more common double‐cone configurations. This design also reduces the height space required for the actuator. When the voltage signal is activated, the robot can achieve horizontal crawling through the action of the central vibrator and lower bristle. The results showed that the robot reached a maximum crawling speed of 110 mm s^−1^ (1.89 times its body length) at 5.5 kV. The load capacity was 17.1 g, with a body mass of 9.6 g. By arranging two DEAs in parallel, the robot was able to turn at an angular velocity of 25.75° s^−1^ at 5 kV and a signal frequency of 79 Hz. However, with dimensions of 58 × 58 × 25 mm^3^, the robot may have limited mobility in narrow spaces.^[^ ^74^ ^]^ Zhu et al. (2023) developed an insect‐scale crawling robot driven by coiled DEAs (see Figure 8B). It was reported that the proposed robot was the smallest and fastest among DEA‐based crawling robots, with a body length of 15 mm, a weight of 0.45 g, and a crawling speed of 60 mm s^−1^ (4 times its body length). The coiled DEAs achieve high‐frequency operation (760 Hz) and a long lifetime (over one million cycles), enabled by well‐designed elastic materials and a 5‐inlet nozzle structure. A key innovation is the 5‐inlet multimaterial coaxial 3D printing technique, which enables automated fabrication of the coiled DEAs with integrated dielectrics and electrodes. The robot also shows high robustness, good environmental adaptability, and impact‐perception capabilities. It can run on various surfaces, climb inside small pipes, work in swarms, and endure repeated impacts. The rational design of the inks used in 3D printing, including the addition of functional fillers and silicone oil, ensures appropriate rheological, electrical, and mechanical properties for the fast and strong actuating responses of the DEAs. The optimized nozzle structure also plays a key role in enhancing the electro‐actuation performance of the DEA fibers by increasing the concentricity of the printed coaxial fibers.^[^ ^75^ ^]^

Hu et al. (2023) designed an inchworm‐inspired soft robot powered by an acrylic stick‐constrained dielectric elastomer actuator (ASCDEA).^[^ ^76^ ^]^ The key contribution is that, unlike many existing inchworm‐like robots, the DEA‐actuated multimodal locomotion‐based soft robot can achieve inverted climbing, vertical climbing, horizontal crawling, and turning locomotion, as well as movement with a heavy load. This is enabled by a novel design using an acrylic stick‐constrained dielectric elastomer actuator (ASCDEA) and electroadhesion actuators (EAAs), coupled with a voltage signal coordination control strategy, as illustrated in Figure 8C. The robot demonstrated various modes of locomotion, including inverted climbing (with a maximum speed of 39.55 mm s^−1^, 0.53 body lengths per second), vertical climbing (maximum speed of 52.62 mm s^−1^, 0.71 body lengths per second), horizontal crawling (maximum speed of 79.55 mm s^−1^, 1.08 body lengths per second), and turning (maximum rate of 16.19° s^−1^). Additionally, the robot was able to crawl at a speed of 13.95 mm s^−1^ while carrying a payload of 130 g, which is 26 times its weight, on a horizontal surface.^[^ ^76^ ^]^ Zhong et al. (2024) developed a crawling robot (Figure 8D) using a 3D scissor mechanism and electrostatic adhesion driven by Dielectric Elastomer Minimum Energy Structures (DEMES). The robot is able to move on multiple surfaces, functioning effectively regardless of which of its four sides contacts the motion plane. By incorporating a symmetrical scissor mechanism and electrostatic adhesion, the robot enables multi‐directional actuation, enhancing its versatility and reducing dependence on specific deployment conditions. The proposed design addresses a key limitation in crawling robot technology, making it highly adaptable to complex and unpredictable environments. The proposed design enhances the robot's multi‐directional functionality, allowing it to continue moving even after landing in a random position. With a square wave voltage of 8.8 kV and an actuation frequency of 1 Hz, the robot achieved a moving speed of 9.32 mm s^−1^.^[^ ^77^ ^]^ Song et al. (2024) reported a DEA bending module consisting of a DE membrane attached to a flexible acrylic frame with a central hollow, as shown in Figure 8E. The design of the bending module allows for easy fabrication and integration into various soft robot applications. The module's performance is predictable using both analytical and finite element method (FEM) modeling, providing a reliable design tool. Furthermore, the bending module was demonstrated in two soft robot applications: a rotatable gripper and a soft‐moving robot, showcasing its versatility and potential for diverse movements, such as bending and linear motion. With an 8 kV power supply, the rotatable gripper and crawling robot equipped with the bending module were able to rotate up to 10.92° and move forward by approximately 5 mm per cycle, respectively.^[^ ^78^ ^]^ Nguyen et al.^[^ ^79^ ^]^ developed a hexapod robot mimicking the alternating tripod gait observed in natural‐legged crawlers such as insects. DEAs drive each leg, providing adaptability and efficiency across flat surfaces. This design emphasizes adaptability and consistency in crawling, which is suitable for applications requiring reliable locomotion over smooth terrains. The 3D‐printing‐based fabrication method simplifies the manufacturing process by embedding the actuators into the robot's frame, and a control system that enables locomotion. The robot's design mimics an insect, using an alternating tripod gait for movement on flat terrain. The multi‐DOF DEA, based on an antagonistic configuration, allows for versatile leg movements and is a key element of the robot's design.

**Table** **3** summarizes the features of crawling robots driven by DEAs in the reviewed literature. Despite the demonstrated flexibility and bio‐inspired adaptability of DEAs, current crawling robots powered by DEAs encounter challenges in enhancing movement speeds and achieving multi‐directional locomotion. The reported movement speed of DEA‐based crawling robots typically varies from a few millimeters to a few centimeters per second. This limitation stems from the necessity for actuation cycles to accommodate the material's elastic recovery and high voltage demands. DEAs, typically made from soft materials like acrylic and silicone with low elastic moduli, allow for large strains and flexible adaptability. However, they may also exhibit low force output, limiting movement speed and payload capacity. Continued efforts are being made to enhance the mechanical performance of these materials; however, these advancements have yet to be implemented in crawling robots to validate their efficacy. Furthermore, the complexity of managing multiple DEAs may limit the ability to achieve specific locomotion modes, such as turning and climbing. The attainment of consistent crawling direction necessitated the presence of effective directional friction. A variety of methods for establishing directional or anisotropic friction were under consideration, including gecko‐inspired dry adhesives, electro‐adhesives, and bio‐inspired claws. An in‐depth analysis of the fundamental principles of locomotion and the interactions between electro‐mechanical coupling and nonlinear material characteristics is necessary. Moreover, additional studies in DEA designs and control strategies are warranted to understand the trade‐offs among movement speed, locomotion, size, and weight.

**Table 3:** Summary of the DEA‐based soft crawling robots in the reviewed studies.

Reference	Actuation Mechanism		Crawler Design			Locomotion and Speed	Key Features
	DEA types	DEA material	Size	Weight	Load Capacity		
Du et al.^[^ ^73^ ^]^	Planar	Silicone rubber Very High Bond (VHB) acrylic film	70×32×2 mm^3^	2.6 g	4.9 g	Crawling: 169.2 mm s^−1^ (maximum) at a peak driven voltage of 6 kV	Rapid locomotion speed Height can be reduced to less than 3 mm Single DOF actuator
Xue et al.^[^ ^74^ ^]^	Single‐layer conical	Silicone rubber Very High Bond (VHB) acrylic film	58×58×25 mm^3^	9.6 g	17.1 g	Turning: average angular velocity of 25.75 ° s^−1^ at a peak voltage of 5 kV and a signal frequency of 79 HZ Crawling: 110 mm s^−1^ at a peak voltage of 5.5 kV	A simple structure that can easily be paralleled Able to conduct “S” shape crawling Limited movement ability in narrow spaces (≤ 25 mm)
Zhu et al.^[^ ^75^ ^]^	Coiled	Silicone rubber with silicone oil	15 mm body length	0.45 g	Three robots can smoothly move forward carrying a 1.3 g load.	Climbing: 30 mm s^−1^ at a slope angle of 10° Caterpillar crawling: 50 mm s^−1^ at a voltage of 9 kV and a frequency of 750HZ	Insect scale, lightweight, and ultra‐fast‐moving speed Able to run in complex maze paths, climb inside small pipes, and work in robot swarms The robot remained motionless at voltages below 6kV Poor load‐carry performance for an individual robot Tethered and non‐autonomous
Hu et al.^[^ ^76^ ^]^	Planar	Acrylic	75 mm body length	5.0 g	130 g	Inverted climbing: 39.55 mm s^−1^ (maximum) Vertical climbing: 52.62 mm s^−1^ (maximum) Horizontal crawling (inchworm‐like): 79.55 mm s^−1^ (maximum) Turning: 16.19° s^−1^ (maximum)	Fast speed Carry high payloads Mult locomotion Tethered and non‐autonomous
Zhong et al.^[^ ^77^ ^]^	Bending	VHB 4910 dielectric elastomer	With paper‐type electrostatic adhesion feet: 52 × 44 × 45 mm^3^ With adaptive electrostatic adhesion feet: 52 × 44 × 49 mm^3^	With paper‐type electrostatic adhesion feet: 4.1 g With adaptive electrostatic adhesion feet: 4.6 g	/	At a frequency of 3 Hz, paper‐based adhesive feet: a crawling speed of 13.6 mm s^−1^; With adaptive electrostatic adhesion feet: crawling speed of 5.2 mm s^−1^.	Can be released from a certain height without requiring direct placement on the plane. Capable of multi‐surface motion
Conn et al.^[^ ^80^ ^]^	Pneumatically‐coupled DE membranes	Acrylic elastomer VHB 4905	80 mm body length	/	/	Inchworm crawling: maximum speed of 4.1 mm s^−1^	Pneumatic coupling Modular design Anisotropic friction for inchworm motion
Henke et al.^[^ ^81^ ^]^	Dielectric elastomer oscillator (DEO)	VHB with carbon electrodes	/	/	/	Maximum crawling speed: 0.83 mm s^−1^	Dielectric elastomer oscillator for self‐controlled actuation No rigid electronics
Henke et al.^[^ ^82^ ^]^	Dielectric elastomer actuators and switches (DEAs and DESs)	Silicone membrane with carbon electrodes	/	/	/	/	Fully soft structure Dielectric elastomer switches for control Electronics‐free
Li et al.^[^ ^83^ ^]^	Dielectric elastomer minimum energy structures (DEMES)	Silicone	49.66 mm diameter	0.88 g	/	Rolling: maximum speed of 47.18 mm s^−1^	Dielectric elastomer actuators Circular design Low mass Mechanical flexibility
Duduta et al.^[^ ^84^ ^]^	Multilayer DEA	Urethane‐acrylic copolymers, polybutadiene	Active area of each actuator component: 20 mm × 20 mm	<200 mg	/	The inchworm‐like robot can travel faster than 1 BL s^−1^	Primarily soft and deformable, with few rigid attachments

**Figure 9:**
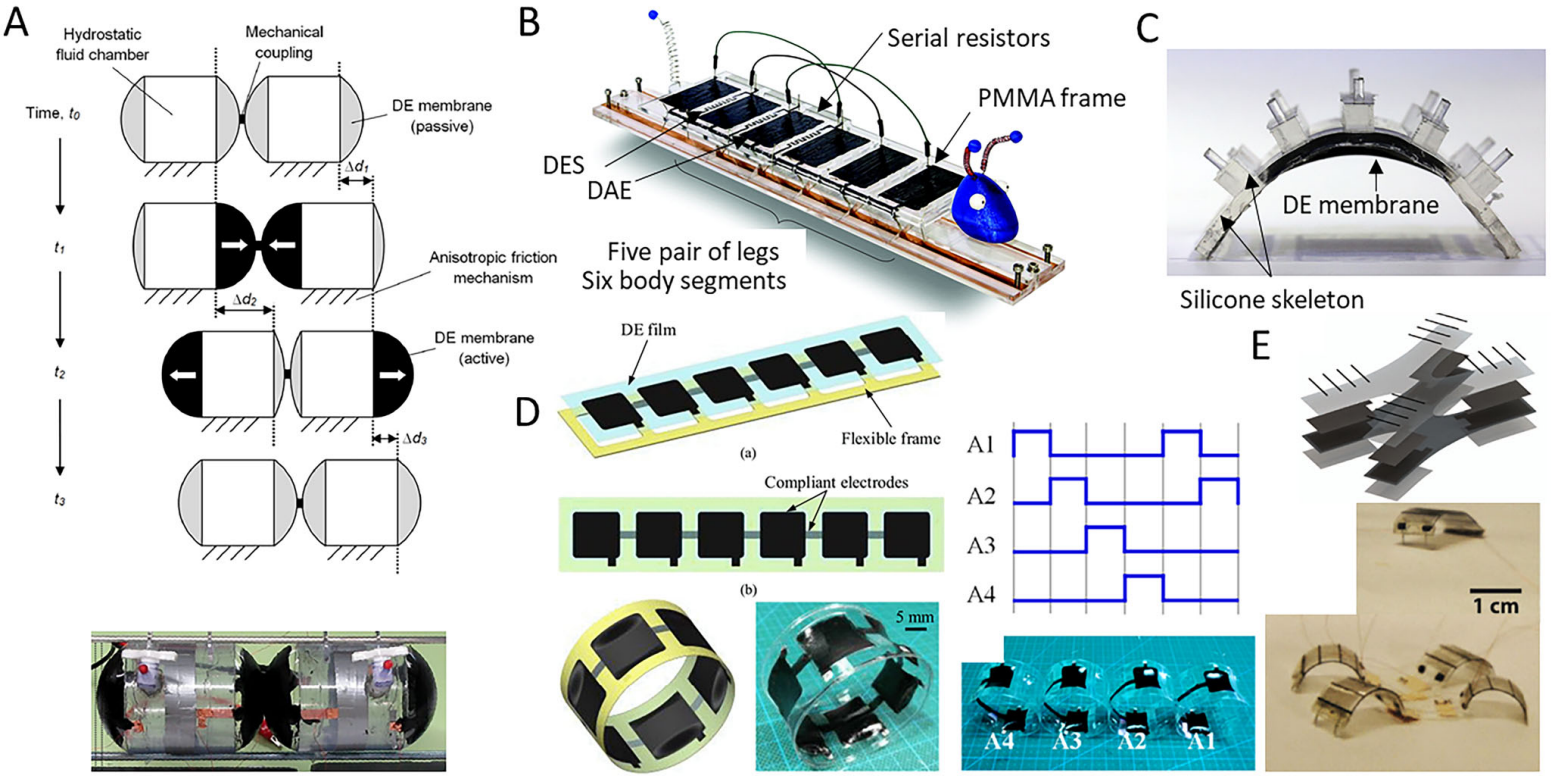
Soft crawlers driven by different configurations of DEA: A) A soft segmented inchworm‐like robot using pneumatically‐coupled DE membranes. Reproduced from Conn et al.^[^ ^80^ ^]^ Copyright 2014, SPIE. B) A multisegmented DAE‐based robot with an integrated controller and DEAs. Reproduced from Henke et al.^[^ ^81^ ^]^ Copyright 2017, Mary Ann Liebert. C) A soft DE crawling structure with a silicone skeleton and stiff frames. Reproduced from Henke et al.^[^ ^82^ ^]^ Copyright 2017, Mary Ann Liebert. D) A fast‐rolling robot based on a multisegmented dielectric elastomer. Reproduced from Li et al.^[^ ^83^ ^]^ Copyright 2018, IEEE. E) A soft inchworm‐like robot and a four‐legged, multi‐gait crawler by using DEAs and carbon fiber stiffeners. Reproduced from Duduta et al.^[^ ^84^ ^]^ Copyright 2017, IEEE.

Conn et al.^[^ ^80^ ^]^ developed a soft‐segmented inchworm‐like robot by using pneumatically coupled DE membranes, as shown in **Figure** **9**A. The robot of a length of 80 mm is fabricated and demonstrated by using two repeated body segments to mimic the peristaltic crawling mechanism. The body length of the robot can be extended by adding more segments. The segment comprises a soft inflatable shell and a pair of DE membranes. The maximum crawling speed is 4.1 mm s^−1^, driven by a voltage of 3 kV with a frequency of 1.5 Hz.

Henke et al.^[^ ^81^ ^]^ developed a multisegmented DAE‐based robot with an integrated controller and DEAs to mimic the crawling motion of the caterpillar, as shown in Figure 9B. The robot crawls driven by high‐voltage dielectric elastomer oscillators (DEO). The self‐regulating DEO can function without conventional electronics by using a closed loop of DE inverters to generate periodic charge control, emulating a biological central pattern generator. This research also introduces a novel production technology for DEOs that improves previous designs by introducing series resistors to control oscillation frequency and increase lifetime. This new method replaces the upper half of NAND gates used in prior designs with simple DE inverters, halving the number of required DEAs and DESs and thus improving reliability. The series resistors define the time constant for charging the capacitors of the DEAs, enabling control over the propagation delay of each stage. This allows for the tuning of the oscillation frequency, which is inversely proportional to the sum of the individual propagation delays and can be adjusted by changing the series charging resistors. This approach is more reliable and tuneable compared to previous designs which lacked electrical elements with controllable values. The power supply is a single DC voltage of 3000–4000 V. The DEAs operate together with dielectric elastomer switches (DES) side‐by‐side to generate a strain‐dependent electrical signal inverter. All functional and supporting parts of the robots are soft polymer and carbon materials, which made the crawler the first completely soft robot without any stiff electronics. Using a very similar DEA design, the team also designed a robot that can crawl and jump by driving it at the resonant frequencies, as shown in Figure 9C.^[^ ^82^ ^]^

The multisegmented DAE concept has innovated the design of soft robots. A fast‐rolling robot, which is configured as a flexible circle based on a multisegmented dielectric elastomer, was developed by Li et al.^[^ ^83^ ^]^ (see Figure 9D). The rolling robot is a fully flexible circular structure constructed by connecting a multisegment dielectric elastomer minimum energy structure (DEMES) end‐to‐end. This design gives the robot a high degree of mechanical flexibility and resilience. The robot weighs 0.88 g with a diameter of 49.66 mm, providing the advantages of low mass, flexibility, and fast response (<50 ms). The speed mass ratio is about 41.22 mm (s·g)^−1^, and the maximum speed‐diameter ratio is ≈0.95 s^−1^. The rolling robot demonstrates the high moving speed and a large speed‐mass ratio. The average speed of the robot is 36.27 mm s^−1^ on the flat ground. Although the rolling motion is different to the crawling motion, the robot is capable of crawling with sequential open‐loop control signals. Alternatively, using an individual rolling robot as an actuating module, a soft crawler can be formed by applying the peristaltic crawling mechanism, as shown in Figure 9D. A detailed fabrication process is also described, utilizing common materials such as acrylic elastomer (3M VHB4905) and carbon grease electrodes. The process includes a non‐equal biaxial pre‐stretch of the elastomer film to give both voltage‐actuated strain and a rollable configuration. The flexible frame is made from laser‐cut PET and is attached to the pre‐stretched DE film.

Duduta et al.^[^ ^84^ ^]^ developed a soft inchworm‐like robot and a four‐legged, multi‐gait crawler by using DEAs and carbon fiber stiffeners. The robots are soft and deformable, with few rigid accessories. To satisfy robot locomotion, the hooks, and straight and bent needles were installed on the posterior end as the feet of the robot to generate differential friction by applying a two‐anchor crawling mechanism. The four‐legged crawler was made by combining multiple inchworm‐like actuators. A novel method was proposed for creating fast‐moving, soft robots using dielectric elastomer actuators (DEAs) without the need for pre‐stretch. The approach allows for the creation of both inchworm and four‐legged crawling robots through a multilayer fabrication process. This fabrication method is relatively quick, enabling the construction of multi‐gait robots in a matter of hours. Figure 9E shows a CAD model of the four‐legged crawler.^[^ ^84^ ^]^ From bottom to top, it contains five layers, including the mylar constraining layers with elastomer on top, electrodes, elastomer covers, and carbon fiber stiffeners. The active area of each DEA component is 20 mm × 10 mm. The robots were driven using a Trek 610E power supply and a Wavetek 75 Arbitrary Waveform Generator. With a square wave voltage of a frequency of 10 Hz, the inchworm‐like robot can travel faster than one body length s^−1^. The four‐legged crawling robot can crawl at a maximum speed of 0.115 body length s^−1^, considerably slower than the inchworm‐like robot. However, the legged crawler is more complex and capable of multiple actuation modes, including a variety of crawling gaits.

Electrically driven DEAs‐based actuators and robots have shown a variety of advantages. Recent work in soft actuation and electronics has simplified the instrumentations and reduced the size of electronic power supplies, resulting in more interest in developing untethered DEAs‐based crawlers. Cao et al.^[^ ^85^ ^]^ developed an untethered soft crawler which consists of a DEA‐based deformable body and two electro‐adhesion actuators acting as robotic feet. The two‐anchor crawling mechanism was used to enable crawling, as shown in Figure 5A. During one crawling cycle, the front foot is initially applied to a voltage of 3 kV, while the rear foot and DEA robotic body remain still without applied voltage. This enables the front foot to adhere to the floor. Then, the rear foot and the DEA body were applied to a voltage of 5 kV, and the applied voltage on the front foot was off. This results in the adhesion of the rear foot and the expansion of the DEA body, which makes the front foot move forward. Repeating these steps cyclically, the untethered robot can crawl effectively with a velocity of 4.16 mm s^−1^ (0.02 body length s^−1^), which is comparable to that of the untethered soft crawling robot based on pneumatic actuators^[^ ^86^ ^]^ or IPMCs.^[^ ^87^ ^]^

#### 3.2.2 Shape Memory Alloys and Shape Memory Polymers

Shape memory alloys (SMAs) and shape memory polymers (SMPs) are unique materials that can “remember” their original shapes and return to them when exposed to specific temperature changes. This property is due to the material's molecular structure, which undergoes a transformation when heated or cooled. For SMAs, this phenomenon typically occurs through a phase change, where the material shifts between a high‐temperature phase and a low‐temperature phase. When the temperature rises, the alloy reverts to its pre‐deformed shape, allowing it to exert force and create movement. Similarly, SMPs utilize their polymeric nature to achieve a memory effect, enabling them to transition between various shapes as they are heated above a certain threshold. This behavior makes them particularly useful in applications such as adaptive actuators, where the ability to alternate anchor points for contraction and release with temperature variations can be effectively harnessed for a variety of engineering and biomedical applications. The unique characteristics of SMAs and SMPs make them invaluable in the fields of soft robotics. As stated by Wood et al.,^[^ ^88^ ^]^ SMAs provide high strength and high‐speed actuation at the micron scale, making them well‐suited for compact and confined spaces. SMAs are generally classified into three types: nitinols, Cu‐based alloys, and Ni‐Ti binary alloys, with nitinols being the most used and widely available.^[^ ^89^ ^]^ However, many of these materials require complex wired connections for vacuum suction, pneumatic control, or Joule heating, which is undesirable for soft robots.^[^ ^90^ ^]^ To address this challenge, more advanced SMAs, such as magnetic SMAs (MSMAs), have been developed. MSMAs offer wireless control, fast responsiveness to magnetic fields, and easy adjustment of field direction and strength, along with a relatively simpler structure.^[^ ^91^, ^92^ ^]^ Magnetic control also has the potential to function independently of other stimuli, allowing actuators to respond to multiple inputs.^[^ ^93^ ^]^

Liang et al.^[^ ^94^ ^]^ developed a soft crawling robot inspired by inchworm locomotion with an adaptive friction mechanism, effectively anchoring forward. Such a mechanism dynamically modulates frictional forces at certain points along the robot's body and establishes stable contact with the surface during contraction phases while reducing resistance during extension phases. The SMA actuator, integrated within a monolithic curved body, leverages differential friction to alternately engage and release the robot's feet to crawl effectively. This design combines one SMA coil with a vertically orientated auxiliary spring and supports the robot's locomotion by synchronizing the body's contraction and recovery. The robot was fabricated using 3D printing with a tailored thermoplastic polyurethane elastomer (TPU). TPU provides good elasticity, abrasive resistance, and thermal insulation. The researchers also created a low‐friction surface using polytetrafluoroethylene (PTFE) and a high‐friction surface using silicone rubber. Such a robot is able to achieve a normalized speed of 0.024 (BL s^−1^) while supporting loads over five times its weight. A crawling robot actuated by SMA springs embedded within a segmented silicone body was developed by Jovanova et al.^[^ ^33^ ^]^ The SMA springs bend each segment of the body. Employing a two‐anchor crawling mechanism, this robot design advanced from the morphological computation to simplify control. The SMA actuation allows the robotic crawlers to transition smoothly between crawling and rolling, adapting to various environmental conditions. Such integration of SMA actuators within segmented structures to achieve controlled smooth movements demonstrates SMA's potential for versatile crawling locomotion in various terrains and adaptive to environmental demands.

**Figure 10:**
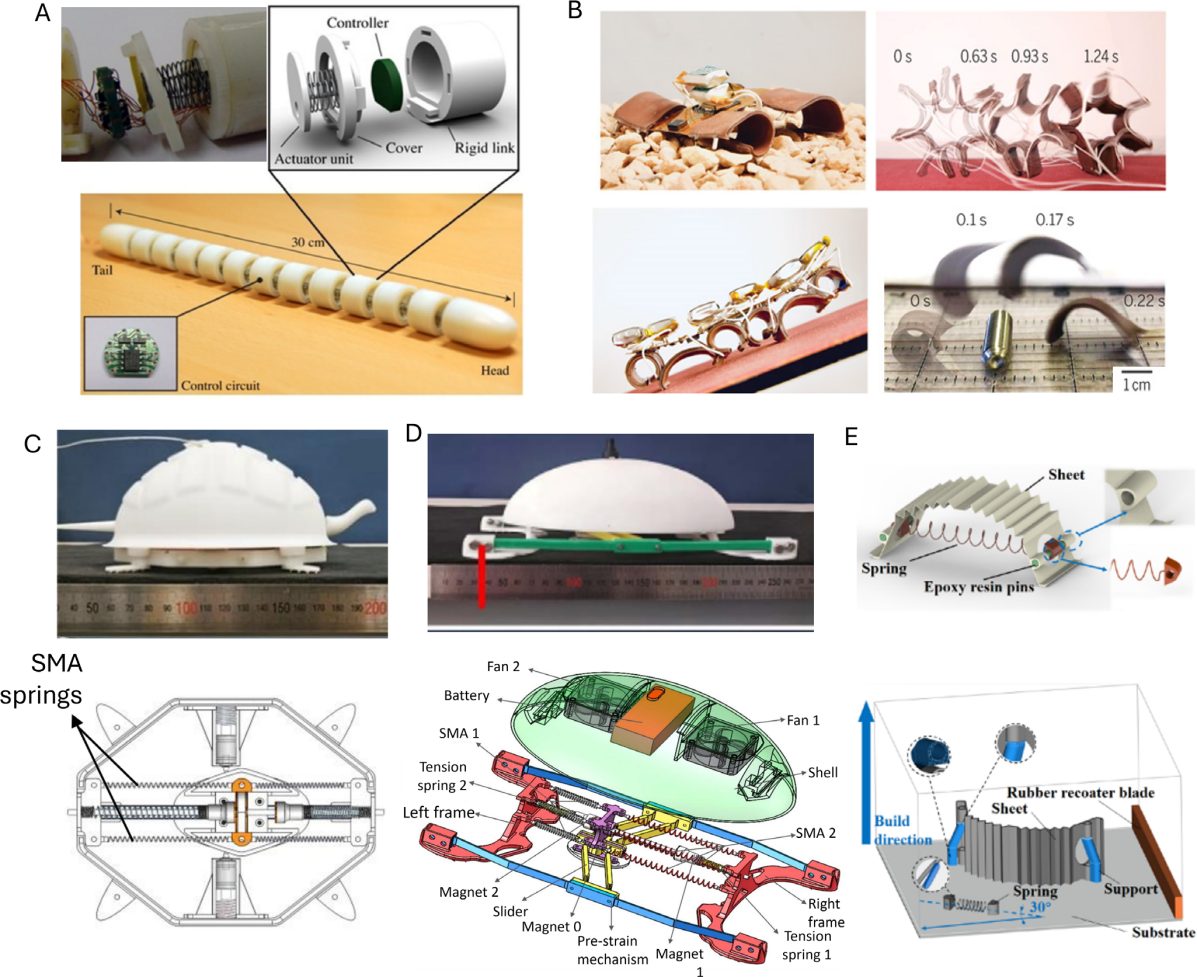
SMA‐actuated soft robots: A) A SMA‐based small crawling robot inspired by C. elegans. Reproduced from Yuk et al.^[^ ^95^ ^]^ Copyright 2011, IOP Publishing. B) Untethered soft robots with SMA actuators walking on a rocky surface, on an incline, leaping over an obstacle, and rolling. Reproduced from Huang et al.^[^ ^98^ ^]^ Copyright 2018, AAAS. C) A SMA‐driven crawling robot utilizing a bistable mechanism. Reproduced from Meng et al.^[^ ^99^ ^]^ Copyright 2019, ASME. D) A mechanically intelligent crawling robot driven by SMA and compliant bistable mechanism. Reproduced from Meng et al.^[^ ^100^ ^]^ Copyright 2020, AMSE. E) A metallic robot prototype with highly flexible SMA structures. Reproduced from Yao et al.^[^ ^101^ ^]^ Copyright 2020, IOP Publishing.

Inspired by the musculature and movement of the nematode Caenorhabditis elegans, Yuk et al.^[^ ^95^ ^]^ developed a crawling robot driven by shape memory alloys (SMAs) that mimics this undulatory motion, as shown in **Figure** **10**E. Thermal SMAs were chosen as actuators due to their functional similarity to the worm's muscles. They can contract by up to 50% of their original length when heated, similar to the worm's muscle contraction. Each SMA actuator is paired with a passive spring to enable contraction and relaxation, mimicking the action of the worm's muscles. This approach overcomes limitations associated with traditional electric motors, which are often too bulky for small robots. The robotic crawler is constructed with 12 SMA units that create sinusoidal undulations, demonstrating the effectiveness of SMAs in achieving bio‐inspired rhythmic crawling with simple binary control. The robot can realize C. elegans‐like motion with a 0.17 HZ undulation frequency, which is comparable to a real worm, unlike the previous biomimetic robot that used peristaltic motion (e.g., earthworm‐inspired designs.^[^ ^96^ ^]^ This robot achieves movement through sinusoidal undulation, highlighting different roles of friction in locomotion. This study shows that SMAs can effectively replicate natural crawling patterns with minimal control complexity, making them ideal for low‐frequency, energy‐efficient movements in confined spaces. Daily‐Diamond et al.^[^ ^97^ ^]^ designed caterpillar‐inspired robots that use SMA actuators. They developed a mathematical model based on rod theory to simulate the robots' forward crawling motion and calculated the ground contact forces to ensure efficient locomotion. By analyzing energy dynamics, they aimed to optimize the efficiency of the actuators, creating a framework that balances power requirements with continuous movement. Their work closely imitates the distributed movement seen in caterpillars, highlighting the potential of SMAs to maintain both efficiency and stability in bio‐inspired designs. The SMA limbs are thinner, more malleable, and have reduced curvature in both actuated and unactuated states. At all pre‐strain levels, the new limbs exhibit actuation positions with nearly zero curvature, making the actuation more controlled. The overall crawling speed is about 0.378 cm s^−1^. Huang et al.^[^ ^98^ ^]^ explored compliant, lightweight SMA actuators designed for rapid, biologically inspired locomotion speeds in untethered soft robots, as shown in Figure 10C. This research addresses a significant challenge in bio‐inspired robotics: achieving high‐speed, dynamic movement comparable to natural organisms by integrating SMAs with soft elastomer structures. It uses a U‐shaped SMA wire embedded between pre‐stretched and non‐stretched layers of thermally conductive elastomer. This design allows for rapid motion and forces comparable to a natural muscle. The pre‐stretch introduces an antagonistic force, which speeds up the transition between actuated and unactuated states, while the thermally conductive tape accelerates heat dissipation, further increasing actuation frequency. The robot demonstrates dynamic, high‐speed crawling with minimal rigid components, enabling efficient operation in confined spaces. The multi‐gait caterpillar‐inspired robot, utilizing flexible circuit boards and SMA actuators, can crawl at a maximum speed of 8 mm s^−1^ for over 25 minutes while carrying six onboard 3.7‐V LiPo batteries. It has a maximum load capacity of 30 g and achieves a peak crawling speed of 74 mm s^−1^ on level ground. Moreover, the robot can crawl up a 23° incline over rocky terrains. The results highlight the potential of SMA technology in mimicking the rapid locomotion of small animals, making it suitable for agile soft robotic crawlers in dynamic environments.

Meng et al.^[^ ^99^ ^]^ introduced a crawling robot with SMA actuators configured with a bistable mechanism, enabling efficient energy storage and release, as shown in Figure 10B. The robot's feet of anisotropic friction design convert reciprocating motion into unidirectional crawling. Integrating a bistable mechanism with SMA actuation minimizes energy expenditure and maximizes movement efficiency, appealing to applications requiring stable, repetitive crawling, especially in industrial or inspection settings. The SMA‐driven crawling robot uses antagonistic SMA spring groups and a cam‐based bistable mechanism for locomotion, with a magnet and tension spring logic system to alternately activate the SMA springs, eliminating the need for electronic control. Its feet feature an anisotropic friction surface with micro‐hooks for unidirectional movement, and integrated cooling fans enhance heat dissipation, increasing actuation frequency and crawling speed. The robot achieves speeds of 0.75 mm s^−1^ to 1.01 mm s^−1^ with applied currents from 1.1A to 1.7A. The team^[^ ^100^ ^]^ also developed a unique approach for SMA‐driven crawling by leveraging a compliant bistable mechanism, as shown in Figure 10D. This approach allows the robot to operate without complex electronic controllers. The SMA actuators, paired with this bistable mechanism, provide reliable reciprocating motion by storing and releasing elastic energy to achieve crawling. This robot's feet are designed to feature anisotropic friction surfaces, converting reciprocating motion into unidirectional locomotion, enabling smooth and energy‐efficient crawling. The robot uses anisotropic friction on its feet to convert the reciprocating motion into unidirectional locomotion. Cooling fans, integrated with the mechanical switch, enhance the SMA cooling rate by 43.75%, thereby increasing the robot's motion frequency. However, the maximum crawling speed only reaches 0.95 mm s^−1^. The relatively low speed is partly due to the integrated batteries, which increase the robot's weight. It also should be noted that low crawling speeds are a common issue for SMA‐actuated robots, as reported speeds range between 0.25 mm s^−1^ and 9 mm s^−1^.

Using the 4D printing technique, Yao et al.^[^ ^101^ ^]^ developed SMA structures for a biomimetic crawling robot inspired by inchworm locomotion, as shown in Figure 10A. The prototype comprises two SMA components that deform antagonistically for controlled segmental crawling. The average forward speed of the proposed robot is 0.0142 BL s^−1^, comparable to most conventional SMA‐driven soft crawling robots. The martensitic sheet, with an axial stiffness of approximately 50 N/m, demonstrates high flexibility. This robot features a fully metallic body actuated by SMAs, offering a novel approach to developing smart, flexible devices and systems with improved deformation control and stiffness adaptability. However, once the load is removed, it cannot fully return to its original shape due to microcrystalline changes in the material, resulting in about 50% plastic deformation. These design advances of soft robots highlight the potential of SMA‐based 4D printing for creating flexible, customized actuators. The selective laser melting used for 3D printing complex SMA structures through precise actuation configurations enables soft crawlers to work for various surfaces. Jovanova et al.^[^ ^33^ ^]^ developed a multi‐locomotion soft robot primarily actuated by SMA. The robot uses SMA springs as actuators, which are embedded between the soft silicone body segments. These springs, made of Nickel Titanium (NiTi), provide the necessary force and strain for movement. The SMA springs are used to generate strain to bend the body and enable both crawling and lifting movements. The robot incorporates multiple smart materials, including one‐way and two‐way SMA springs and magnets, which could be further developed using magneto‐active materials for improved modularity. Numerical modeling indicates that a single segment can achieve a crawling speed of ≈0.05 m s^−1^. However, the overall speed of the robot depends on factors such as the number of segments, the terrain, and the actuation sequence. The prototype demonstrated the ability to perform a 90° rotation during a rolling maneuver in 4.64 s. Gul et al.^[^ ^102^ ^]^ developed a tri‐legged soft robot inspired by spider locomotion powered by Bio Metal Fiber (BMF), an SMA acting as artificial muscles. It uses a spider‐like multi‐step gait driven by controlled BMF contraction and relaxation and can achieve a crawling speed of 2.7 mm s^−1^ at 5 Hz with 3 V and 250 mA on smooth surfaces. The soft robot is constructed using two flexible materials, epoxy and polyurethane, which are printed in three layered steps. The researchers developed a unique in‐situ UV curing 3D printing process to fabricate the multi‐material soft robot. This method uses a customized rotational multi‐head 3D printing system with multiple wavelengths curing lasers. However, the robot's structure can handle a maximum signal frequency of 5 Hz. Beyond this frequency, it risks exploding due to rapid actuation and the resulting internal temperature increase. Guo et al.^[^ ^103^ ^]^ demonstrated a crawler fabricated using hydrogen‐bonded liquid crystal elastomers (LCEs), which can undergo reversible actuation in response to several stimuli. In this design, the required friction anisotropy for inducing net movement is achieved by programming the shape of the actuator strip. Modifying the actuator geometry, which alters the contact angle between the strip and the surface, results in an increase in the crawling speed of the robot. The shape of the crawler can be adjusted to accommodate movement within confined spaces, such as glass tubes. Transitioning into a flat temporary state facilitates traversal through narrow pathways, and upon exiting, the original curved actuator profile is restored. This highlights the effectiveness of integrating shape‐memory programming with reversible actuation to enable mobility in restricted environments. A soft robot inspired by the caterpillar Manduca sexta and powered by shape memory alloy (SMA) actuators was designed and fabricated by Daily‐Diamond et al.^[^ ^97^ ^]^ The design models a series of actuators as a system of rigid bodies connected by torsional springs, which are actuated based on motion patterns reflective of individual caterpillar segments. During experimental studies, the flexural rigidity and curvature of the SMA actuator were measured as a function of actuation voltage, and the successful forward locomotion allowed an approximate speed of 0.3780 cm s^−1^. These findings provide a strong foundation for extending the elastic rod framework to model and analyze individual SMA actuators and their collective undulatory behavior. The study also compares the energy costs and other performance metrics of the analytical elastic model with the numerical model. It is noted that the power required to provoke undulatory motion is similar in magnitude and trend for both the SMA robot and the elastic model, suggesting that the continua approximation can lead to a ballpark of energetics required for sequentially organized crawlers.

Wang et al.^[^ ^104^ ^]^ introduce a bio‐inspired shape‐memory structural color (SMSC) hydrogel film inspired by hummingbird feather color‐adjusting mechanisms. This film combines structural color, achieved through an inverse opal scaffold structure, with the shape‐memory capabilities of N‐isopropylacrylamide (NIPAM) and stearyl acrylate (SA) copolymers. The structural color is angle‐dependent due to Bragg diffraction. The shape‐memory effect is achieved through the thermal responsiveness of the SMPs. The film is fabricated using a sacrificial template method with self‐assembled silica nanoparticles and can be programmed to form temporary shapes in hot water and return to its original shape in cool water. The incorporation of graphene quantum dots (QDs) enables light‐controlled reversible deformation via near‐infrared (NIR) light. The SMSC hydrogel film has the potential for use as soft actuators, sensors, and dynamic anti‐counterfeiting labels. Sun et al.^[^ ^105^ ^]^ highlight the use of biological actuators, including cardiomyocytes, skeletal muscles, and microorganisms, for movement. These actuators are integrated with synthetic materials, often soft materials such as PDMS or hydrogels, that are designed to mimic physiological environments. Various control mechanisms, including **optical, electrical, chemical, and magnetic stimuli**, are discussed. Different locomotion modalities, such as swimming, pumping, walking, and gripping, are also explored, showcasing the diverse capabilities of these systems. However, improving the lifespan of biological components and creating more complex, multifunctional systems of current biohybrid robots should be further addressed. **Table** **4** summarises the reviewed soft robots based on shape memory alloys and polymers.

**Table 4:** The reviewed soft robots based on shape‐memory alloys and polymers.

Reference	Actuation Mechanism	Robot Design			Locomotion and Speed	Key Features
		Size	Weight	Load Capacity		
Jovanova et al.^[^ ^33^ ^]^	SMA springs	/	/	/	Crawling: approximately 0.05 m s^−1^ Rolling: 90° rotation during 4.64s	Multi‐Segmented Design Magnetic enhancement
Liang et al.^[^ ^94^ ^]^	An SMA actuator with an elastic body and auxiliary spring	54 mm length	9.77 g	can withstand a load of 50 g	0.024 BL s^−1^	Utilizes a single SMA coil, elastic body, and vertical spring for efficient actuation. Supports loads over five times its weight Compact, adaptable, and easy to control
Yuk et al.^[^ ^95^ ^]^	A thermal SMA actuator	30 cm length 2 cm width 1.5 cm height	50 g	/	sinusoidal undulating motion: 0.17 BL s^−1^	SMA‐based actuation mimicking the body wall muscle anatomy of C. elegans A simple binary motion control mechanism inspired by the muscular activations of C. elegans during locomotion
Huang et al.^[^ ^98^ ^]^	SMA	/	/	30 g	Maximum crawling speed on level ground: 74 mm s^−1^ Can crawl up a 23° incline and over rocky terrains	Compliant and lightweight actuators that can generate rapid motions and large forces Reduced hardware complexity, size, and weight
Meng et al.^[^ ^99^ ^]^	Two antagonistic SMA spring groups, each with two parallel SMA springs	/	/	/	Crawling speeds ranged from 0.75 mm s^−1^ to 1.01 mm s^−1^	Reduced the constraints of the electronic controller through the logic of the robot mechanism itself. More effective and reliable motion through a cam‐based bistable mechanism. Bandwidth increased significantly due to the introduction of cooling fans. Lightweight and untethered for detection and rescue in narrow spaces.
Meng et al.^[^ ^100^ ^]^	SMA actuators with a mechanical logic switch and a compliant bistable mechanism	280 mm × 20 mm × 18 mm	354 g	Maximum experimental output force: 8.83 N	Up to 0.95 mm s^−1^	Reduced cooling time and enhanced motion frequency A compliant bistable mechanism for stable positions and explosive output force during movement. Relatively low crawling speed
Yao et al.^[^ ^101^ ^]^	SMA: a curved sheet and a spring coil	/	/	Maximum load of 50 N	0.0142 BL s^−1^ The average speed of the rear foot was 0.427 mm s^−1^	4D printing that can pre‐program responsive shapes during 3D fabrication Flexibility and deformation control with the use of rigid materials Enhanced environmental adaptability
Gul et al.^[^ ^102^ ^]^	Actuated by Bio Metal Fiber (BMF), a commercially available SMA that acts as an artificial muscle	Diameter: 80 mm Width (each leg): 5 mm Depth (each leg): 5 mm	/	/	On a smooth surface, a maximum forward speed of 2.7 mm s^−1^ at a signal frequency of 5 Hz, with an input voltage of 3 V and 250 mA.	Tri‐legged soft robot mimicking the structure and movement of spiders Multi‐material 3D printing
Guo et al.^[^ ^103^ ^]^	Liquid crystal elastomers (LCEs) with dynamic hydrogen bonds	24 mm (L) × 2 mm (W) × 0.1 mm (H)	/	/	/	Reversible actuation (photo) Programmable shape Friction required controllable
Daily‐Diamond et al.^[^ ^97^ ^]^	SMA	5.5 cm length 2.4 cm width 0.1 cm height	1 g	/	Overall crawling speed is approximately 0.3780 cm s^−1^	Thinner, more malleable, and less curved SMA limbs Undulatory locomotion Binary actuation Modular structure

DEAs outperform general EAPs in response speed due to their actuation mechanisms. DEAs operate through electrostatic forces created by an electric field between compliant electrodes, causing expansion in width and contraction in thickness. Their response time, constrained by charge transfer and mechanical viscoelasticity, ranges from milliseconds to microseconds for thin films. DEAs are flexible and have low mechanical damping, allowing quick deformation and high actuation forces. In contrast, ionic EAPs, like Ionic Polymer‐Metal Composites (IPMCs), rely on ion migration and have slower response times in seconds due to ion diffusion. Electronic EAPs, such as ferroelectric polymers, experience internal molecular rearrangements, making them faster than ionic types but slower than DEAs. Ionic EAPs face challenges with higher internal resistance and viscosity, leading to longer motion delays.

As shown in Tables 3 and 4, electric field actuation has significantly contributed to developing DEAs and SMAs‐based soft crawlers. DEAs utilize electrostatic forces to achieve lightweight and high‐energy‐density actuation, resulting in smooth and nearly silent motion. This unique characteristic renders them particularly suitable for robotic crawlers operating in environments where noise minimization is essential. In addition to their actuation capabilities, DEAs can also function as advanced sensors, enabling self‐sensing abilities in soft crawlers and robots, thereby enhancing their responsiveness and adaptability to a variety of tasks. However, the operation of DEAs often necessitates high‐voltage inputs, which can complicate both design and safety considerations. Furthermore, issues related to material degradation over time can significantly impact the reliability and lifespan of these actuators. SMAs utilize the Joule heating effect to trigger phase transformations in the material. This mechanism enables them to generate substantial force, although acutely slower in actuation speed compared to DEAs. While SMAs are effectively used in soft robotic structures to create powerful movements, they encounter their own limitations, including prolonged cooling periods, susceptibility to fatigue, and energy losses associated with heat dissipation. These factors can impact the overall efficiency and functionality of systems relying on SMAs. The combination of these technologies presents exciting possibilities, including hybrid actuators that merge the fast response of DEAs with the high force output of SMAs. Advances in material science, such as more efficient elastomers and fatigue‐resistant SMAs, are crucial for enhancing their longevity and performance.

Electrically actuated soft crawlers leverage smart materials that deform under an applied electric field, enabling precise, rapid, and lightweight actuation. These systems use dielectric elastomers (DEs) and ionic electroactive polymers (EAPs) due to their high flexibility, fast response times, and tuneable mechanical properties. DEs exhibit large deformations under high voltage, making them suitable for high‐strain applications, while ionic EAPs operate at lower voltages with efficient ion transport, ideal for bio‐inspired soft actuation. Nanocomposites, such as graphene, carbon nanotubes (CNTs), and metallic nanoparticles, have significantly improved the strength, conductivity, and self‐healing properties of soft crawler actuators, enhancing their durability and sensing capabilities. Material optimization, including the incorporation of nanocomposites and conductive fillers (e.g., carbon nanotubes, graphene), enhances electrical conductivity, mechanical robustness, and actuation efficiency, making these soft actuators more durable and responsive. Programmable materials, including shape‐memory polymers and liquid crystal elastomers (LCEs), provide soft crawlers with adaptive shape control and environmental responsiveness.

Fabrication techniques for electrically actuated soft robots include thin‐film deposition, 3D printing, and microfabrication, each offering different precision and scalability levels. Thin‐film processes like spin‐coating produce uniform, stretchable electrodes crucial for dielectric elastomers. Advanced 3D printing facilitates customized geometries with embedded conductive pathways, while microfabrication techniques, such as photolithography, allow precise microscale structuring for miniaturized actuators. These methods enhance actuation speed, efficiency, and control, making soft robots ideal for high‐frequency motion, fine deformation, and adaptability. Their low weight and biocompatibility broaden their applications in medical robotics, wearable technology, and soft artificial muscles. Addressing these challenges will boost the adaptability and efficiency of soft crawlers in biomedical, environmental, and exploration robotics.

#### 3.2.3 Piezoelectric Materials

Piezoelectric materials produce a strain in response to an applied electric field due to a change in polarization via the converse piezoelectric effect. As a result, they are used for actuation^[^ ^106^ ^]^ and have been employed to produce soft robotic crawlers. These materials produce an electrical charge in response to applied stress via the direct piezoelectric effect and are used for sensing forces or strains in soft robots.^[^ ^107^ ^]^ One advantage of these materials is, therefore, their multi‐functionality, where the same material can be used for both actuation and sensing within a smart system by providing feedback for control of any soft robot.

The range of piezoelectric materials employed to achieve crawling is diverse. This includes piezoelectric ceramics, such as lead zirconate titanate (PZT),^[^ ^108^ ^]^ which exhibit relatively high piezoelectric strain coefficients (*d*_ij_), which is a measure of the strain per unit applied electric field. These ceramic‐based piezoelectric materials are typically high stiffness, allowing the delivery of relatively large actuation forces. However, the inherently high Young's modulus (>40 GPa), low mechanical flexibility, low toughness, and brittleness of ceramics make their application in soft robotic systems more challenging.

An alternative to piezoelectric ceramics is piezoelectric polymers, such as poly(vinylidene fluoride) (PVDF) or its co‐polymer, poly‐ [(vinylidene fluoride)‐co‐trifluoroethylene] (PVDF‐TrFE). The piezoelectric polymers typically exhibit piezoelectric strain coefficients that are an order of magnitude lower than the piezoelectric ceramics. However, the materials exhibit a lower stiffness (<5 GPa), are mechanically flexible and have a high toughness, thereby making them better suited to integration within soft robotic structures. To produce a material that is capable of combining the high piezoelectric strain coefficients of ceramic materials and the attractive mechanical properties of the piezoelectric polymers, significant effort has been made to create composite systems that combine high strain ceramics; in this case, the ceramic is in the form of particles^[^ ^107^ ^]^ or fibers,^[^ ^109^ ^]^ within a polymer matrix.

**Figure 11:**
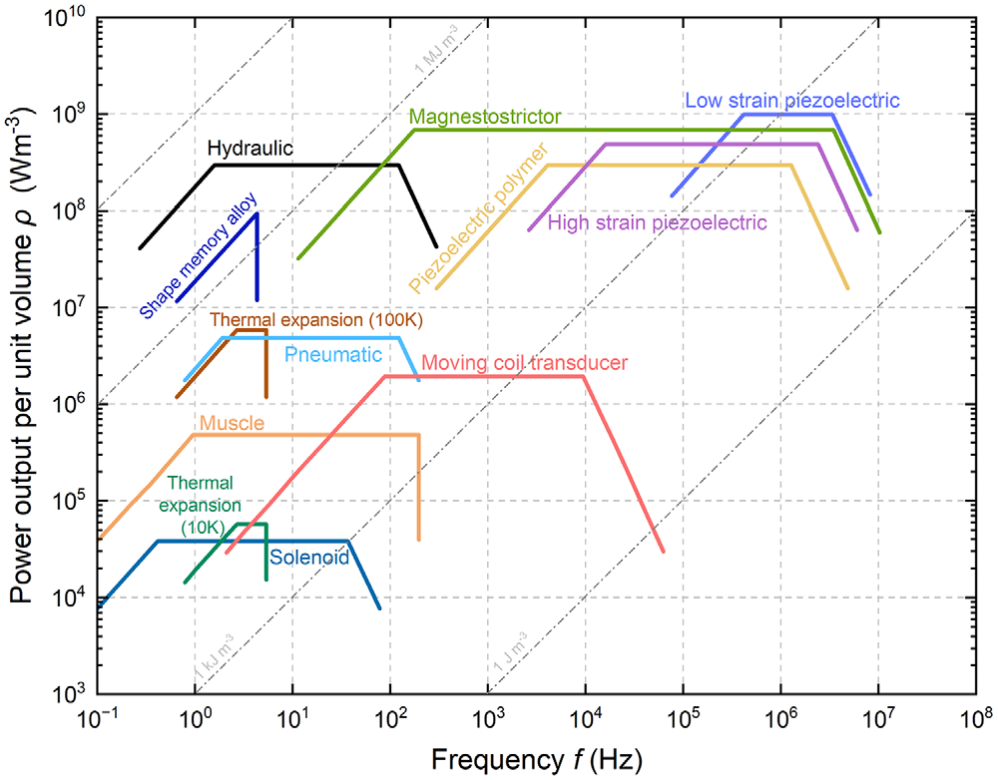
Volumetric power, p (W m^−3^), versus frequency, f (Hz), for various actuators. Lines of gradient of unity correspond to energy density per cycle (J m^−3^). Reproduced from Huber et al.^[^ ^112^ ^]^ Copyright 1997, The Royal Society.

The maximum strain levels of piezoelectric materials are ≈0.1–0.3% and are therefore low compared to shape memory alloys, dielectric elastomers, and pneumatic/hydraulic actuators. To produce the large deflections that are needed for crawling, there is a need to i) amplify the strain, for example, by exploiting bending, ii) accumulate a series of small‐scale deflections by actuating at a high frequency, in particular at the resonant frequency of the soft robot, or iii) use both bending and resonance.^[^ ^110^, ^111^ ^]^ In this regard, since the origin of the piezoelectric effect is due to a change in the polarization of an atomic/molecular dipole, they are particularly suited to actuation at the high frequencies associated with resonance (up to MHz), as compared to other actuation mechanisms. Since the piezoelectric actuation mechanism employed to produce robot locomotion tends to operate cyclically and at resonance, the maximum operational frequency, power, and efficiency are of relevance. **Figure** **11** provides a comparison of the operating frequency, volumetric power (a measure of the mechanical power output per unit initial volume while cycling, W m^−3^), and bandwidth of a range of actuators, whereby it can be seen that an advantage of piezoelectric materials is their ability to produce a high volumetric power by operating at high frequencies (up to MHz). The lines in Figure 11 with a gradient of unity correspond to energy density per cycle (J m^−3^). We will see that the ability of piezoelectric materials to provide a high frequency of operation and high‐power output per unit volume enables small displacements to be accumulated to produce robot motion.

Soft crawling robots have been reviewed by ref. [113], which included a small overview of piezoelectric materials, particularly PVDF, while Tang et al.^[^ ^114^ ^]^ have recently summarized recent patents related to soft crawling robots, including piezoelectric fiber‐based composites. Due to their inherently low stiffness, the majority of piezoelectric‐based soft robots that are produced employ polymeric piezoelectric materials, which are now overviewed.

In terms of piezoelectric polymer‐based soft crawling robots,^[^ ^115^ ^]^ reported on a rapid and robust insect‐scale soft robot that was developed by taking inspiration from the movement of a cockroach. A PVDF piezoelectric polymer was used, that was based on a curved unimorph piezoelectric structure. Using this bioinspired approach a crawling robot was developed that was able to achieve a relative speed of 20 body lengths per second (BL s^−1^); this was the fastest among the published insect crawling robots. The high compliance and toughness of the piezoelectric polymer were exploited to enable the robot to withstand the weight of an adult footstep, which was approximately 1 million times greater than the weight of the robot. Hoffman et al.^[^ ^116^ ^]^ investigated a miniature centipede‐inspired robot, a scalable small‐sized design that retains adaptability to rugged surfaces. This robot mimics centipede locomotion by utilizing multiple segments for effective ground contact, improving the robot's stability and movement over various terrain conditions.

**Figure 12:**
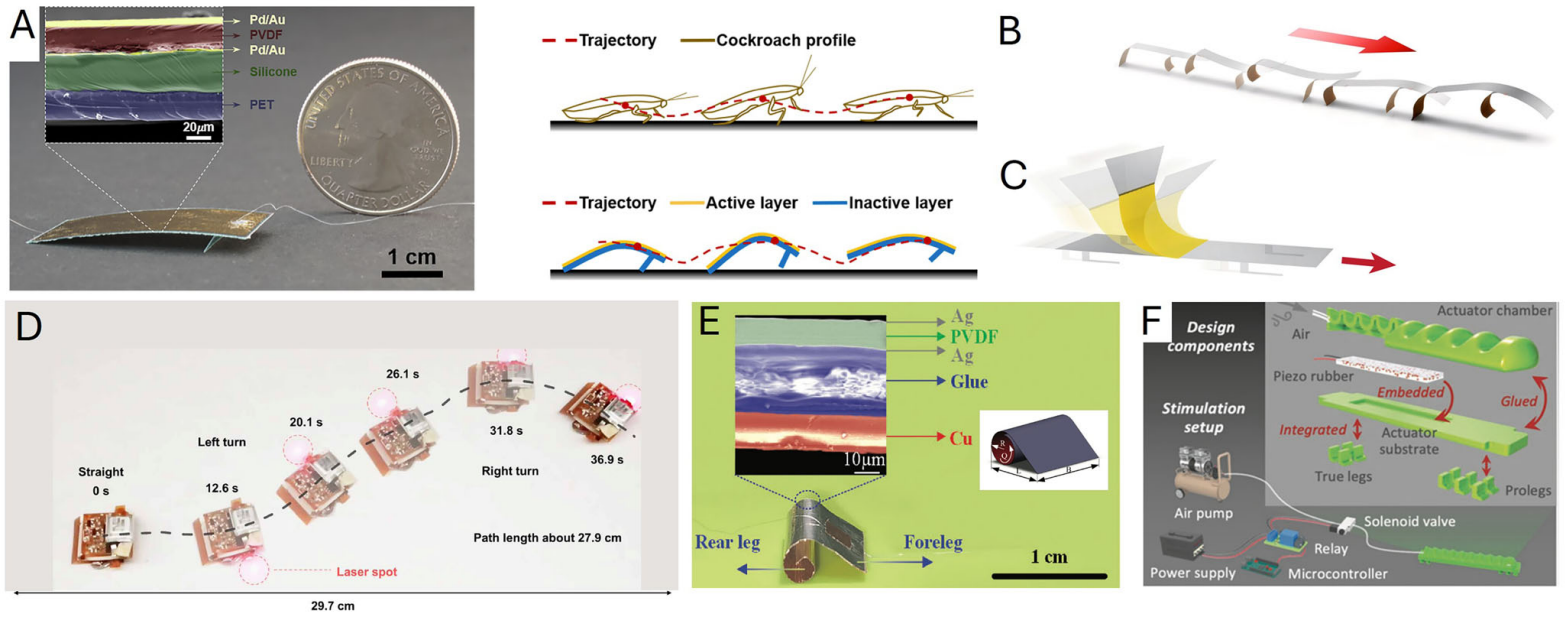
A) Optical image of robot connected with two electrical wires to the top and bottom electrodes, and comparison of the wavelike running paths showing the movements of the COM of a cockroach.^[^ ^115^ ^]^ Reprinted with permission from AAAS. B) Overlapped images of mobile robot, which consists of a main body and two legs, and the materials employed are flexible and electrically activated.^[^ ^117^ ^]^ Licensed under a Creative Commons Attribution 4.0 International License. C) Trajectory manipulation demonstration of an untethered robot with the combination of straight, left, and right turn motion adjusted by a laser on a photoresistor.^[^ ^118^ ^]^ Licensed under a Creative Commons Attribution 4.0 License. D) Schematic of the design and assembly of the untethered robot.^[^ ^119^ ^]^ Reprinted with permission from AAAS. E) Prototype and working principle showing a soft‐bodied robot rolled into a spiral shape.^[^ ^121^ ^]^ Reprinted with approval from John Wiley and Sons. F) Design of the piezo‐wormbots that consists of a soft actuator embedded with piezoelectric rubber and true and prolegs. Stimulation setup is designed with the air pump, power supply, microcontroller, relay, and solenoid valve.^[^ ^107^ ^]^ With permission, the publisher for this copyrighted material is Mary Ann Liebert, Inc. publishers.

**Figure** **12**A shows the 3 cm × 1.5 cm crawling robot, which consists of a curved robot body with a short leg‐like structure located at its front end.^[^ ^115^ ^]^ The inset shows a cross‐sectional view of the robot, indicating an unimorph structure that was composed of an 18‐µm‐thick piezoelectric PVDF layer, two 50‐nm‐thick palladium (Pd)/gold (Au) layers that acted as upper and lower electrodes to apply an electric field. An inactive layer was used to produce a bending moment and consisted of a lower 25‐µm‐thick silicone adhesive and a 25 µm‐thick polyethylene terephthalate (PET) substrate. By applying an alternating electric field at about 800 Hz via the electrodes, the PVDF layer produces a periodic extension and contraction due to the converse piezoelectric effect that changes the shape of the curved robot body; the existence of a maximum speed at a specific frequency indicates activation of a resonant mode. The combination of the shape change and the friction of the short front leg with the floor leads to an oscillatory center of mass (COM) trajectory pattern that is similar to a cockroach crawling. Potential applications under consideration were environmental exploration, structural inspection, reconnaissance, and disaster relief.

A PVDF‐based soft‐legged robot proposed in ref. [117] was inspired by quadruped vertebrates. The soft robot was based on a flat main body with two pre‐curved legs, where the use of a low‐stiffness piezoelectric PVDF material provided resilience when the robot was subject to mechanical loads or impact. Figure 12B shows the overall structure, where the main body length was 50 mm with a width of 10 mm, and legs were 20 mm long. The main body of the soft robot was actuated by a bimorph PVDF actuator, which was driven via an input square wave with DC 65 V ± AC 65 V. Their work experimentally demonstrated that the mobile robot was able to move 70% of its body length per second and the robot could survive impulse shock via a hammer.

Lim et al.^[^ ^118^ ^]^ reported on a thin piezoelectric polymer‐based mobile robot that had a curved piezoelectric tail. In contrast to the work of Park and Cha,^[^ ^117^ ^]^ the robot was turned upside down, whereby the legs of Park and Cha^[^ ^117^ ^]^ were redesigned to form a tail that provided propulsion. The main body was fabricated from a non‐piezoelectric polyethylene terephthalate (PET) polymer so that the soft robot was able to operate on wet surfaces due to the electrical insulation afforded by the PET material. To generate a thrust for locomotion, the piezoelectric curved PVDF tail was driven at the resonant frequency of the piezoelectric structure; see Figure 12C.^[^ ^118^ ^]^ Again, the soft nature of the materials employed was thought to provide enhanced resilience to the robot. The proposed soft robot was able to move at an average velocity of 5.6 mm s^−1^, which was approximately 11% of its body length per second.

The work of Wu et al.^[^ ^115^ ^]^ was later extended byLiang et al.^[^ ^119^ ^]^ to include two electrostatic footpads to elicit rotational motion to enable steering, whereby the crawling soft robot was able to carry a 180‐milligram on‐board sensor to record gas concentrations during and identify the location of gas leakage, in particular for use in confined spaces.^[^ ^120^ ^]^ As an important advancement in the field of piezoelectric soft crawling robots, an untethered version of the soft robot was produced (2.4 cm by 2.2 cm, 240 mg) that carried a payload of 1.66 g, which included a battery, two photoresistive sensors, and a flexible circuit board; Figure 12D.^[^ ^119^ ^]^ This was used to demonstrate trajectory manipulation and power autonomy by operating at a resonant frequency of approximately 410 Hz. Using a laser beam, the resistance of the photoresistor decreases, which was used to regulate the applied voltage to crawler footpads to enable the robot to turn.

Inspired by Archimedean spiral geometries, also known as an isokinetic spiral, that is widely encountered in nature,^[^ ^121^ ^]^ produced a fast spiral‐shaped soft robot that was manufactured by creating a composite structure that included an amorphous PVDF film and a passive layer of copper. The robot was able to produce a speed of 78 body lengths per second (BL s^−1^) when actuated at its first mode of resonance (247 Hz). Interestingly, a reverse motion could also be achieved of 11 body lengths per second at its third mode of resonance (700 Hz). Figure 12E shows images of the millimeter‐scale of the robot dimension with a length of 7.5 mm and width of 10 mm, which was based on a five‐layer asymmetric structure that was based on a 12‐µm‐thick piezoelectric PVDF film, two 100‐nm‐thick silver electrodes coated on both sides of the PVDF to provide an electric field, a 30‐µm‐thickness acrylic adhesive layer and a 20‐µm‐thick copper layer.^[^ ^121^ ^]^ When an electric field is applied to the piezoelectric PVDF layer, it shrinks or expands in the length direction, and the passive copper layer helps to produce a bending moment to balance the internal stress; the structure, therefore, acts as an unimorph piezoelectric actuator. The advantages of producing a soft robot with a spiral structure were thought to be that i) the structural asymmetry facilitates directional locomotion that is driven by bending without the need for additional legs, ii) the horizontal displacement is enlarged compared to that of sheet structure due to the bending moment that is generated, iii) contact between the robot and the ground acts to stabilize motion compared to other soft robotic structures. The robot was able to multi‐task and climb slopes, jump, carry loads, and steer; the motion was reported to be similar to that of a hopping kangaroo. An untethered robot powered by an onboard battery with a flexible circuit could move at 20 mm s^−1^.

The spiral design was expanded by,^[^ ^122^ ^]^ who employed cheetah‐like motion, which produced rapid motion by expansion and contraction of cheetah limbs; this was achieved by driving at the resonant frequency of a double spiral robot structure. A 0.058 g and 10 mm long tethered robot was able to produce a speed of 42.8 body lengths per second and a turning speed of 482 ° s^−1^ by connecting two robots in parallel, thereby surpassing the turning speed of most soft and rigid robots with good agility. The structure was shown to be able to operate after flipping over and could move over complex terrains, climb slopes, and carry loads, including thermal sensors.

A soft robot based on a polyvinylidene difluoride/polyimide (PVDF/PI) unimorph actuator, reported in ref. [123], was actuated at its resonance frequency (f = 300 Hz) and achieved a maximum speed of 10 cm s^−1^ and a turning rate of 89.7° s^−1^. Electrostatic pads were used to provide improved friction. The robot was powered by a triboelectric nanogenerator to demonstrate the potential for harvesting. A micro‐camera was equipped with the soft robot to provide real‐time video streaming of the soft robot, enhancing its detection capability.

Piezoelectric materials also produce charge in response to an applied stress as a result of the direct piezoelectric effect. In this regard,^[^ ^107^ ^]^ designed a flexible “piezo‐wormbot” that enabled the soft robot to continuously crawl without external manipulation, as shown in Figure 12F. The design was based on an actuator that was used to generate a bending motion during inflation, and a flexible piezoelectric rubber was employed to generate a sensing voltage, which triggered deflation. Legs were applied to the bending actuator to convert the bending and recovery sequence into a continuous crawling mode by mimicking the “true legs” and “prolegs” of inchworms. Initially, an air pump was kept open (i.e., ON for inflating) that inflated the pneumatic actuator to deform the piezo‐wormbot and induce a bending motion. This led to a piezoelectric rubber located in the piezo‐wormbot body producing a voltage signal, which initiated deflation when the voltage was sufficiently high, and the voltage then reduced. As a result of the repeated inflation and deflation cycles, a maximum crawling speed of 16.6 mm s^−1^ was achieved, resulting in a maximum body length (BL) per second of 0.13 BL s^−1^. The piezoelectric rubber was fabricated by distributing 80 wt.% of lead zirconate titanate (PZT) ceramic particles in a flexible silicone rubber. A high fraction is PZT is necessary to maximize the piezoelectric activity. The piezoelectric composite was then poled by application of an electric field of 60 kV cm^−1^ at a temperature of 150 °C for 30 min to align the ferroelectric domains/dipoles of the composite film in a common orientation. Finally, electrical cables were connected to the piezoelectric rubber composite to measure the voltage signal from the piezoelectric sensors. The piezo‐worm‐bots are considered as a potential solution for the continuous autonomous actuation of biomimetic soft robots. Piezoelectric fiber composites, which provide lower stiffness and greater degrees of curvature compared to dense piezoelectric ceramics, have been used in crawling soft robots in constrained environments^[^ ^124^ ^]^ and jumping robots.^[^ ^125^ ^]^

**Table** **5** summarizes the application of piezoelectric materials in soft crawling robots in the reviewed studies. Piezoelectric materials, particularly PVDF, have proven highly advantageous for soft crawling robots due to their flexibility, low power consumption, and fast response times. However, despite these advantages, piezoelectric materials also have their limitations in soft robotics. As reviewed, the soft robots actuated by PVDF have limited load capacities, up to 15 times the robot's own weight, which may limit their scalability and broader application. The reliance on high‐voltage inputs, as seen in most PVDF‐driven designs, has also been a disadvantage. One potential way to address power supply limitations could be integrating energy harvesting technologies, but the practical deployment of self‐powered soft robots is still in its early stages. The mechanical durability of piezoelectric materials under continuous deformation remains a concern, especially for long‐term high‐load or high‐electric field operations. The fabrication methods employed to date to integrate piezoelectric materials and composites with soft robots are relatively manual and involve bonding the active material to the robot body. However, the relative simplicity of the device structures produced provides significant scope for manufacture at scale and even automation.

**Table 5:** Summary of piezoelectric soft robots across different studies.

Reference	Material and configuration	Robot design			Locomotion and speed	Key features
		Size	Weight	Load capacity		
Jiao et al.^[^ ^107^ ^]^	Piezoelectric rubber embedded in mechanical metamaterial	12 mm (L)	16 g	Pushed box with 50% of the robot's mass (8 g)	Maximum crawling speed of 16.6 mm s^−1^ (0.13 BL s^−1^)	Continuous crawling without external manipulation
Wu et al.^[^ ^115^ ^]^	Curved polyvinylidene fluoride (PVDF) unimorph with palladium (Pd)/gold (Au) electrodes	10 mm (L)	0.024 g	0.406 g	Maximum running speed: 20 BL s^−1^ 7 BL s^−1^ when climbing a slope with an angle of 15.6°	Fast insect‐scale soft robot Ultra robust Locomotion generated by a large vibration amplitude and a bouncing gait mechanism Can function with a low voltage supply of only 8 V
Park and Cha^[^ ^117^ ^]^	Bimorph PVDF actuator with pre‐curved legs	Body: 50 mm (L) x 10 mm (W), Legs: 20 mm (W)	0.32 g	/	Animal‐like running motion, 70% of body length per second (∼35.3 mm s^−1^)	Flexible piezoelectric legs, which allow it to maintain movement under impulsive shock The active leg's ground‐striking motion improves the robot's mobility
Lim et al.^[^ ^118^ ^]^	Curved PVDF with polyethylene terephthalate (PET) fin	Body: 15 mm (L) x 50 mm (W)	/	/	Average moving speed of 5.6 mm s^−1^	The attached fin can generate oscillation. Able to work in harsh environments such as narrow and wet areas Adaptability, easy to customize the materials
Liang et al.^[^ ^119^ ^]^	Curved unimorph PVDF with titanium (Ti)/gold (Au) electrodes	3 cm (L) x 1.5 cm (W) (tethered) 2.4 cm (L) x 2.2 cm (W) (untethered)	65 mg (tethered) 240 mg (untethered)	1.66 g (untethered)	The centripetal acceleration of 28 BL s^−2^ (tethered) A moving speed of 7.81 BL s^−1^ and a turning rate of 482 ° s^−1^ (tethered) Linear moving speed: 3.4 BL s^−1^ (untethered)	Comparable centripetal acceleration compared to the cockroach Can detect ethanol leakage Simplified turning regulation Ultrahigh agility and trajectory manipulations
Liang et al.^[^ ^120^ ^]^	Unimorph PVDF with PET legs	3 cm (L) x 1.5 cm (W)	106 mg	/	Forward speed: maximum 7 cm s^−1^ Turning speed: 1.8 rad s^−1^	Asymmetric and simple structure, Trajectory control via frequency adjustment
Mu et al.^[^ ^121^ ^]^	Polymeric piezoelectric bilayer in a spiral shape	20 mm x 20 mm	0.15 g	1.665 g	orward: 76 BL s^−1^ Backward: 11.26 BL s^−1^	Can escape from a relatively confined space without external control and human intervention Multitasking, such as climbing slopes, jumping steps, and high maneuverability High speed, more than three times faster than other reported insect‐scale soft robots
Chen et al.^[^ ^122^ ^]^	PVDF with Ag electrode, spiral structure	10 mm (L) x 10 mm (W)	0.058 g	0.464 g	aximum motion speed: 42.8 BL s^−1^, Maximum average turning speed: 482 ° s^−1^ Able to climb a slope with a 12° inclination at a rate of 5.4 BL s^−1^	Double spiral structure, flipping ability, and adaptability to complex environments High‐speed movement, more than double that of reported piezoelectric soft robots Fast turning, surpassing most soft and rigid robots
Lu et al.^[^ ^123^ ^]^	Unimorph PVDF layer with electrostatic footpads	24 mm (L) x 22 mm (W)	350 mg	/	Maximum moving speed: 10 cm s^−1^ Turning: 89.7° s^−1^	Triboelectric nanogenerator‐driven Self‐powered Electromechanical conversion efficiency of the PVDF actuator is 15.3%
Zheng et al.^[^ ^124^ ^]^	Piezoelectric macrofiber composite bonded onto flexible steel foil	500 mm (L) x 20 mm (W), 20 mm legs	/	/	Crawl speed decreased from 0.17 cm/cycle to 0.07 cycles when crawling under a step‐shaped roof	Able to perform inchworm‐like crawling under roofs with height constraints

In summary, the key contributions of piezoelectric materials to the field of soft crawling robots involve the use of soft piezoelectric polymers and polymer‐matrix composites that exploit their high‐frequency operation to achieve resonance and maximize the deflection to achieve high‐speed crawling. The displacement of the piezoelectric component is also often further amplified by inducing a bending moment in the robot structure. Inspiration has been taken from nature to mimic the movement of insects and mammals, whereby the high volumetric power of piezoelectric materials, shown in Figure 11, has allowed relatively small robots to be produced. This has led to piezoelectric soft robots being considered for operation in constrained or narrow environments. Piezoelectric materials have also been used as a sensing element to be employed in combination with other forms of actuation, such as the “piezo‐wormbot”.^[^ ^107^ ^]^

### 3.3 Magnetic Field Actuation

Magnetic fields play a crucial role in actuating soft robots, particularly in microrobot applications, including drug delivery, biomedical devices, and treatment methods. Soft crawlers are typically designed by dispersing magnetic fillers—such as iron particles or other ferromagnetic materials—into a soft elastomer matrix, providing flexibility and movement adaptability. The soft crawlers can elongate and contract for navigation, bend for maneuverability, and even leap to overcome obstacles. The effectiveness of these motions is determined by several key parameters, including the magnetization profile of the fillers, which refers to how the magnetic properties are distributed within the material. Additionally, the actuation signal plays a vital role in controlling the magnets; variations in the strength and direction of the magnetic field can dictate the type of motion produced. Finally, the overall shape and configuration of the filler materials significantly influence the deformation patterns of the soft crawlers, allowing them to adapt to different environments and tasks. Together, these factors are essential for optimizing the performance and functionality of soft robotic systems in practical applications.

**Figure 13:**
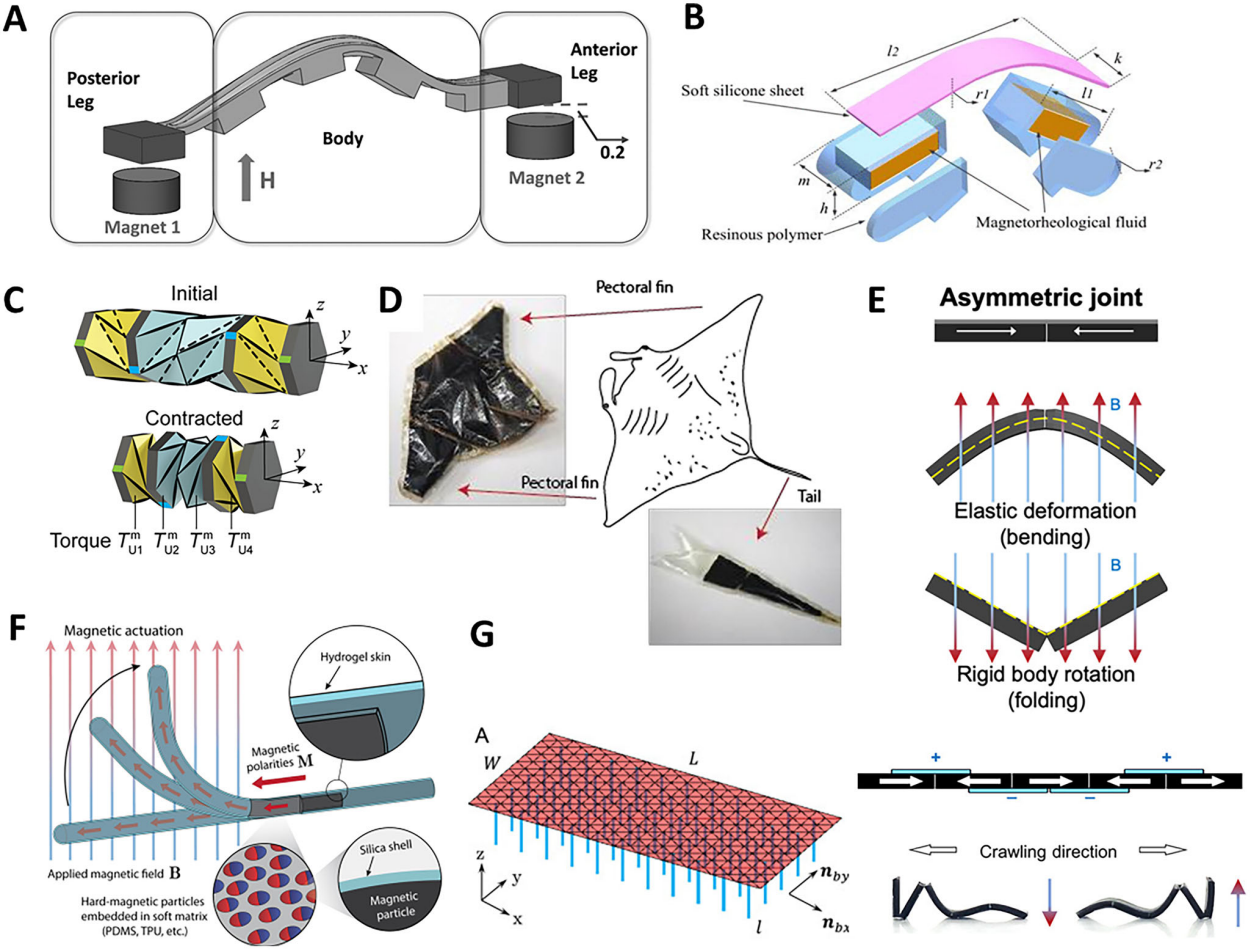
A) CAD model showing posterior and anterior magnets placed at two legs of a fully 3D printed inchworm‐inspired soft robot with magnetic actuation. Reproduced from Joyee and Pan.^[^ ^126^ ^]^ Copyright 2019, Mary Ann Liebert. B) Schematic of the separate structure of the magnetorheological fluid (MRF)‐filled soft crawling robot. Reproduced from Hua et al.^[^ ^127^ ^]^ Copyright 2020, IEEE. C) Schematic of the configurations and magnetization directions at the initial state and the contracted state of a soft robotic origami crawler. Reproduced from Ze et al.^[^ ^37^ ^]^ Copyright 2022, AAAS. D) Prototype of the pectoral fins and tail of the ferromagnetic biomimetic robot crafted from thin layers of polyurethane and polyethene films, sealed with a heating device to create natural contour shapes. Reproduced from Leon‐Rodriguez et al.^[^ ^135^ ^]^ Copyright 2015, World Scientific. E) A soft crawler with dual‐direction crawling motion Sunder a switching magnetic field. Reproduced from Wu et al.^[^ ^136^ ^]^ Copyright 2019, ACS Publications. F) Schematic of the magnetically responsive tip of the ferromagnetic soft continuum robot with programmed magnetic polarities resulting from the hard magnetic particles embedded in the robot's body made of soft polymer matrix. Reproduced from Kim et al.^[^ ^138^ ^]^ Copyright 2019, Mary Ann Liebert. G) Schematic of a magnetic cilia carpet robot using discrete differential geometry formulation. Reproduced from Huang et al.^[^ ^139^ ^]^ Copyright 2023, Elsevier.

Joyee and Pan^[^ ^126^ ^]^ developed a 3D‐printed, inchworm‐inspired soft robot that leverages magnetic actuation for wireless, untethered movement, as shown in **Figure** **13**A. Integrating multiple material composites with programmed magnetic properties enables the robots to generate controlled linear crawling motion in confined spaces. The robot is lightweight, noise‐free, and highly deformable, utilizing an anchor push‐pull locomotion inspired by the inchworm. It features two‐way linear actuation with a stride length of 5 mm (about one‐eighth of its body length), a linear speed of 1.67 mm s^−1^, and an impressive locomotion efficiency of 93.28%. The robot can achieve a maximum bending deformation of 4.5 mm in the *z*‐direction. It can carry up to 5.95 g (≈30 times its own mass) on a smooth surface and can support 1.5 times its body weight while stably climbing a 45° inclined surface. The magnetic field provides an external adjustable stimulus, which varies the crawling gait with simple internal control systems. This design highlights the potential of magnetic field actuation to simplify control and improve adaptability in compact soft robots. Hua et al.^[^ ^127^ ^]^ introduced a soft crawling robot actuated by magnetorheological fluid (MRF) (see Figure 13B), a unique material that changes viscosity in response to magnetic fields. The MRF within the robot's body produces magnetic torque, allowing efficient and forceful crawling without a physical connection to an external power source. Such a magnetic field actuation approach enables the robots to alternate between single‐step and continuous crawling. The developed MRF robot is lightweight, weighing only 0 .77g. The robot can crawl at an average speed of 1.9 mm s^−1^ at a magnetic operating frequency of 0.62 Hz and a ground roughness of 0.6 µm, which reduces to 1.6 mm s^−1^ with a ground roughness of 1.2 µm. The robot can also crawl at 1.7 mm s^−1^ at a gradient angle of 18°, with a ground roughness of 0.8 µm and a magnetic operating frequency of 0.62 Hz. Soft and adaptable robotic crawlers with MRF actuation are appealing in achieving precise control and navigation, especially in compact environments.

Inspired by centipede locomotion, Xu et al.^[^ ^128^ ^]^ developed an untethered, legged soft robot with magnetic field actuation. By embedding silicone with magnetic particles, the robot achieves adaptable‐legged crawling in response to external magnetic fields. Magnetic field actuation enables the robot to efficiently transverse diverse environments, unraveling potential in industrial and biomedical applications where robust and untethered movement is desired. The legged structure enhances both speed and stability, underscoring magnetic field actuation's effectiveness in multi‐legged bioinspired robots. The experimental results demonstrate the robot's ability to achieve high‐speed locomotion, ranging from 0.536 to 1.604 mm s^−1^ across various surfaces, including paper, wood, and PMMA. A centimeter‐scale worm memetic soft crawling robot, MagWorm, embedded with magnetic patches along its body, was developed by Niu et al.^[^ ^129^ ^]^ Emulating natural worms, this design configuration allows MagWorm to interact with the external magnetic field to achieve wave‐like crawling motion. The robot produces repetitive and directional crawling movements. The three‐ and five‐segment robots can crawl at maximum speeds of 0.36 BL s^−1^ and 0.4 BL s^−1^, respectively. It is demonstrated that MagWorm crawls faster than those robots actuated by pneumatic technology or SMA, whose speeds are generally less than 0.1 BL s^−1^. The robot can carry a load of ≈75% of its body weight while still crawling. This demonstrates magnetic field actuation's capability to produce smooth, controllable, and untethered crawling in optimizing small and lightweight robots to move through tight and confined spaces, making it particularly suited for enclosed spaces requiring directional control and adaptability. Inspired by inchworm and millipede crawling movement, Venkiteswaran et al.^[^ ^130^ ^]^ utilized a magnetic‐polymer composite material to explore multi‐legged motion in magnetic field‐actuated biomimetic soft robots. The robots crawl on uneven terrains effectively through controlled magnetic field actuation. This research delves into the potential of magnetic actuation technology for developing advanced, flexible, multilegged robots. These robots are designed to navigate challenging environments, including complex and uneven surfaces that hinder traditional robotic systems. This capability is especially significant for biomedical applications, where the ability to maneuver precisely in confined or irregular spaces is crucial. With their compact design, these multilegged robots can operate effectively in situations requiring meticulous control and a small footprint, such as within the human body or in delicate surgical environments. The integration of magnetic actuation enhances their adaptability and ensures they can perform precisely, paving the way for innovative solutions in the healthcare field. Ze et al.^[^ ^37^ ^]^ developed a magnetically actuated origami crawler using a Kresling origami structure, as shown in Figure 13C. The robot is capable of in‐plane contraction, using a magnetic field to provide both propulsion and steering. Through magnetic actuation, the robot can navigate confined spaces by adjusting its stiffness. The robot's axial stiffness is low (8.2 kPa) for easy contraction under small magnetic fields, while its lateral stiffness is high (93–970 kPa) to prevent structural damage. Magnetic fields (−10 to −30 mT) can tune and reinforce axial stiffness up to 14.1 kPa, enabling adaptable mechanical behavior in different directions and allowing for precise and versatile movement patterns. Programing stiffness and maintaining geometric flexibility enables the robot to steer and crawl effectively. The crawling speed reached 13.2 mm s^−1^ when the magnetic field's magnitude and frequency were 40 mT and 5HZ, respectively. In addition, the magnetically actuated crawler enables multidirectional navigation without additional mechanisms or actuators. It can promptly align its magnetization direction with the magnetic field as its angle changes counterclockwise to 60°, 120°, 180°, 240°, and 300°. This design demonstrates the effectiveness of magnetic field action in compact and untethered soft robots.

Chen et al.^[^ ^131^ ^]^ developed a reprogrammable magnetic soft robot using low melting alloys (LMA) embedded with magnetic microparticles to dynamically adjust environmental changes with a simple mechanical design. The crawlers suit biomedical and industrial applications in reconfigurable devices requiring high adaptability. The LMA reorientates and fixes the robot's magnetization profile, switching between different crawling modes and adapting its shape for complex tasks. This reconfigurability highlights magnetic actuation's potential in multi‐functional adaptive robots. Manamanchaiyaporn et al.^[^ ^132^ ^]^ utilized a deformable magnetic elastomer structure to create a triangular head‐tail millimeter‐scaled soft robot that moves through lateral undulation. The robot (2mm width, 9.5 mm length, and 80µ m thickness) is driven by oscillating magnetic fields, which generate dynamic torque on the head, causing the tail to wave passively. The robot's swimming velocity reaches approximately 3.4 mm s^−1^ under a magnetic field of 12 mT with an oscillation frequency of 8 Hz. It can undulate in tubes with diameters of 3 mm, 8 mm, and 12 mm under magnetic fields of 6 mT, 9 mT, and 12 mT, respectively. This motion allows the entire body to propel itself through a series of lateral body waves. This motor‐less mechanism uses a magnetic field, which induces body deformation due to magnetic alignment. With sine‐based magnetization, the triangular head‐tail shape of the soft robot takes advantage of magnetic compliance for lateral undulation mobility. As a result, it produces controlled and fluid propulsion, reminiscent of the undulatory locomotion seen in natural crawlers. This design offers the robot a versatile locomotion system that requires minimal control efforts. Such adaptability is especially promising for biomedical applications, where self‐propelled miniature soft robots can enhance minimally invasive treatments.

Du et al.^[^ ^133^ ^]^ developed an untethered millirobot that features magnetic actuation in the head and functional capabilities in the tail. This is achieved by integrating control, actuation, and sensing directly into the materials, allowing the robot to exhibit multimodal locomotion and adapt to its environment. The millirobot demonstrates controllable and transformable crawling, swinging, and rolling with this soft and asymmetric structure. The iRobot (length 6 mm, width 4.0 mm, height 174 µm) exhibits exceptional obstacle‐crossing and task‐performing abilities, swimming over a 1.3 cm obstacle (over twice its body length) in 14 seconds, then crawling away in just 4 seconds. It can also switch between various locomotion modes under rapid magnetic field orientation changes. The iRobot crawls through a 2 mm tunnel at 0.83 mm s^−1^ and navigates a 450 µm channel at 0.18 mm s^−1^ underwater. It can also shrink to fit into a 3 mm cubic tube, crawl through it in 24 s, and then return to its original size. This robot outstands in obstacle crossing. Moreover, the robot can squeeze its body easily to crawl through a tube via near‐infrared irradiation, triggering the osmotic shrinking of its body. Possessed with extraordinary environmental adaptive functions, such designs seamlessly integrate magnetic actuation with hydrogel properties to create a versatile robot for changing environments where camouflage or dynamic responses are in demand. These smart systems demonstrate magnetic actuation's role in enabling environment‐sensitive multifunctional robots. Zheng et al.^[^ ^134^ ^]^ designed a soft robot inspired by ascidians, featuring magnetically actuated segments that can crawl, tumble, and transport objects. The robot achieves controlled and precise crawling and transportation functions by utilizing sinusoidal magnetic fields. The robot's crawling velocity increased with input frequency, peaking at 0.22 mm s^−1^ (0.022 body lengths s^−1^) at 9 Hz. It showcases its drug release capability, with a 10 mT magnetic field guiding the robot to the first target, where a 60 mT field releases the drug by damaging the capsule, followed by the robot tumbling to the second target for the second release. This design emphasizes the significance of magnetic field actuation in enhancing the robot's range of motion and functional diversity, allowing for various types of locomotion within a compact structure. Such capabilities are particularly beneficial for biomedical applications that require precision control and are critical for applications such as targeted drug delivery and micro‐manipulation.

A soft crawler (see Figure 13D) inspired by the fluid dynamics of a manta ray, employing ferrofluids that are controllable via magnetic fields, was developed by León‐Rodriguez et al.^[^ ^135^ ^]^ The body of the robot, which is filled with ferrofluid, produces smooth, wave‐like movements through magnetic actuation. This conceptual design illustrates the capabilities of magnetic actuation in facilitating continuous and flexible locomotion within compact robotic systems, effectively emulating both fluidic and biomimetic movements. This research examines magnetic actuation as a viable alternative for the control and minimization of soft robot dimensions, presenting a significant option when compared to traditional mechanical and electromechanical biomimetic systems and shape memory alloys.

To enable multimodal actuation in soft crawlers, Wu et al.^[^ ^136^ ^]^ designed an asymmetric magnetic‐responsive composite with active metamaterials. This magnetic‐responsive composite embedded in a soft matrix (see Figure 13E) uses programmable magnetic alignment to generate crawling, even swimming, and other locomotion modes, enhancing adaptability across diverse terrains and highlighting magnetic actuation's versatility in creating complex and dynamic movements. The robot demonstrates a stable crawling speed of up to 34 mm s^−1^ (0.56 BL s^−1^), significantly faster than reported magnetically driven robots, which crawl at less than 0.24 BL s^−1^. Kim et al.^[^ ^137^ ^]^ introduced an innovative approach to 3D printing materials with embedded ferromagnetic domains, enabling rapid and untethered shape transformations in response to external magnetic fields. By precisely aligning magnetic particles during the printing process, the researchers developed structures capable of crawling and performing other complex movements, such as rolling and jumping, without the need for tethered power or intricate control systems. The robot uses 3D‐printed soft materials with programmed ferromagnetic domains for rapid shape transformations via magnetic actuation, as shown in Figure 13F. Printed with elastomer composites and ferromagnetic microparticles, it enables auxetic behaviors and high actuation speed. The robot performs functions like crawling, rolling, jumping, and catching fast‐moving objects. Its auxetic structure achieves a leap speed of 250 mm s^−1^, moving 120 mm in 0.7 s by reversing the magnetic field direction. This sudden reversal of the field direction quickly increases the magnetic potential energy and triggers the drastic release of the stored elastic and magnetic potential energy. The team^[^ ^138^ ^]^ also developed a ferromagnetic soft continuum robot capable of maneuvering through highly constrained environments with magnetic actuation. A soft polymer matrix embedded with ferromagnetic microparticles allows robots to steer and move omnidirectionally through complex, narrow spaces, such as cerebrovascular paths. With self‐lubricating hydrogel skin, the robot reduces friction to a large extent, enhancing its ability to navigate tortuous paths with minimal resistance. The team further extended the capability of the ferromagnetic soft continuum robots to navigate complex and constrained environments. The continuum robot navigated vessels with diameters ranging from 2.5 to 7.5 mm, covering an overall distance of 250 mm. The prototype, with an outer diameter of 600 µm, successfully navigated through three aneurysms, making sharp turns using magnetic actuation at 20–80 mT. It reached the first aneurysm at 11 s, the second at 15 s, and the third at 25 s, continuing downstream by 36 s. Combining magnetic actuation and soft materials, this design demonstrates potential applications in minimally invasive medical procedures, especially in locations beyond where traditional instruments could reach.

Huang et al.^[^ ^139^ ^]^ introduced a magnetic cilia carpet robot, employing an array of cilia‐like structures embedded with magnetic particles in an elastomer matrix to achieve precise and adaptive locomotion, as shown in Figure 13G. The robot is able to crawl at about 12 cm s^−1^ at a frictional coefficient of 0.2 and a magnetic field frequency of 20 rad s^−1^. By employing discrete differential geometry (DDG) to model the deformation of each cilium, the robot achieves smooth, wave‐like crawling motions using an external magnetic field for actuation. This locomotion strategy coordinates the movement of the cilia sequentially, mimicking the undulating motion observed in organisms like centipedes. By adjusting the strength and direction of the magnetic field, the robot's cilia can bend, extend, and contract to create forward crawling movements. This approach allows the robot to adapt its gait, enabling efficient navigation across complex and uneven terrains while maintaining high movement precision. Additionally, the design minimizes the need for intensive computational power, leading to reduced energy consumption. The combination of high precision and versatility in controlled movement and the robot's adaptability highlights its potential for applications in constrained environments, such as search and rescue missions, exploration in unknown spaces, and biomedical navigation. Khan et al.^[^ ^39^ ^]^ developed a hybrid electromagnetic and soft‐toe foot structure. The combination of rigid components and compliant, soft toes provides strong adhesion on metallic surfaces and versatile adaptability on curved surfaces, enabling efficient crawling in industrial settings. Segmented foot movements provide precise, incremental steps that mimic legged crawling. Instead of the traditionally rigid leg approach, this design employs a foot structure, achieving a desired balance of rigid and compliant components combination, enabling controlled, discrete movement across metallic and uneven surfaces. **Table** **6** summarizes the soft robots that use magnetic field actuation.

**Table 6:** Summary of soft crawlers that use magnetic field actuation.

Reference	Material and Configuration	Design			Locomotion and Speed	Key Features
		Size	Weight	Load Capacity		
Ze et al.^[^ ^37^ ^]^	assembling Kresling units (polyethene film), magnetic plates (Ecoflex 00–30 silicone embedded with 40% hard magnetic particles), and feet (PDMS)	28 mm body length	0.95 g	/	crawling speed 13.2 mm s^−1^ at 40 mT and 5 Hz of magnetic field and frequency	Origami inspired Can overcome large resistances from severely confined spaces Can potentially serve as a minimally invasive device for biomedical applications
Joyee and Pan^[^ ^126^ ^]^	particle–polymer composite structure with magnetic particles embedded in the anterior and posterior leg	40 mm (L) x 2.5 mm (W) x2 mm (H)	0.2 g	0.38 g	linear crawling speed 1.67 mm s^−1^	Multimaterial additive manufacturing Lightweight, noise‐free, and highly deformable Anchor push–anchor pull locomotion strategy
Hua et al.^[^ ^127^ ^]^	soft silicone sheet and 3‐DP filled with MRF	24 mm (L) x 4.2 mm (W) x 4.4 mm (H)	0.77 g	/	maximum average speed 1.9 mm s^−1^ at 0.62 Hz, ground roughness of 0.6 µm	Magnetorheological fluid (MRF)‐filled soft crawling robot High feasibility Fast magnetized/demagnetized ability
Xu et al.^[^ ^128^ ^]^	the blending of magnetic particles with silicone polymer	/	/	/	maximum crawling speed 1.604 mm s^−1^	Custom‐made electromagnetic coils High‐speed locomotion Eight‐Legged
Niu et al.^[^ ^129^ ^]^	magnet patches embedded in silicone rubber EcoFlex™ 00–50	50 mm (L) x 7 mm (H)	/	/	maximum crawling speed 38.8 mm s^−1^ of five‐segment robots	Actuated by an array of permanent magnets Controlled by a distant magnetic field Minimalistic combination of magnetic and soft parts
Venkiteswaran et al.^[^ ^130^ ^]^	silicone rubber mixed with isotropic powder made from praseodymium‐iron‐boron (PrFeB)	40 mm (L) x 5 mm (W) x 5 mm (H)	/	/	maximum crawling speed 0.4 mm s^−1^	Controllable and repeatable straight‐line motion Controlled by an array of six electromagnetic coils
Chen et al.^[^ ^131^ ^]^	low melting alloys (LMA) embedded with magnetic microparticles	20 mm (L) x 5 mm (W) x 0.4 mm (H)	/	/	continuous overturning motion 8 mm/cycle rolling 10 mm/cycle	Strip‐like robot Can dynamically adjust to environmental changes with a simple mechanical design Can be selectively programmed in different temperature ranges
Manamanchaiyaporn et al.^[^ ^132^ ^]^	silicone rubber embedded with the anisotropic magnetization could	9.5 mm (L) x 2 mm (W) x 0.08 mm (H)	0.0041g	/	maximum speed of 4.5 mm s^−1^ at a frequency of 6.5 Hz	Driven by oscillating magnetic fields High degree of freedom Self‐propulsion
Du et al.^[^ ^133^ ^]^	NdFeB microparticles added into a NIPAM prepolymer solution and dispersed sufficiently with ultrasound	6 mm(L) x 0.174 mm (H)	/	/	crawl through the 2 mm height tunnel: average speed 0.83 mm s^−1^ underwater swing through a 450 µm wide channel: average speed 0.18 mm s^−1^	Helical propulsion in water Outstanding obstacle‐crossing abilities Extraordinary environmental adaptive functions
Zheng et al.^[^ ^134^ ^]^	silicone rubber (Ecoflex 0020) with neodymium‐iron‐boron particles (NdFeB)	12 mm (L) x 10 mm (D)	/	/	/	Inspired by the contraction motion of the ascidian EMA powered Create a different motion under the magnetic field actuation
León‐Rodriguez et al.^[^ ^135^ ^]^	ferro‐fluid with magnetic particles coated with a stabilizing dispersing agent (surfactant)	5 cm body length	/	/	/	Manta ray‐inspired Ferro‐fluid actuated Can move in both directions and make 360‐degree turns
Wu et al.^[^ ^136^ ^]^	magnetic‐responsive composite embedded in a soft matrix	60 mm body length			maximum crawling speed 34 mm s^−1^ maximum swimming speed 23 mm s^−1^	Frog inspired Fast‐transforming actuation Multifunctional joint design
Kim et al.^[^ ^137^ ^]^	3D printing ink with magnetizable microparticles of neodymium–iron–boron (NdFeB) alloy and fumed silica nanoparticles embedded in a silicone rubber matrix containing silicone catalyst and crosslinker	/	/	8 g	/	Hexahedral structure Achieve movement by rolling‐based locomotion Can carry an object with an arbitrary shape
Kim et al.^[^ ^138^ ^]^	main body: elastomer composite with magnetizable microparticles (5‐µm‐sized on average of a NdFeB alloy (28–30) soft polymer matrix: composed of either silicone [polydimethylsiloxane (PDMS)] or thermoplastic polyurethane (TPU) elastomers	3 mm (L) x 0.6 mm (D)	/	/	/	Submillimeter‐scale Self‐lubricating Achieve omnidirectional steering and navigating capabilities
Huang et al.^[^ ^139^ ^]^	Array of cilia‐like structures embedded with magnetic particles in an elastomer matrix	/	/	/	able to crawl and roll	Cilia‐inspired Discrete differential geometry (DDG) framework Reduce energy consumption

Magnetic field actuation has emerged as an advantageous method for enabling remote untethered motion in soft robotic crawlers. These robots can achieve fast, adaptable, and multi‐modal locomotion by integrating ferromagnetic particles, magnetorheological fluids, or patterned magnetic domains into soft materials. Magnetic actuation offers a unique balance of wireless control, rapid response time, and precise directional manipulation, which make magnetically driven crawlers highly effective for constrained environments, especially in biomedical applications. Magnetic soft robots are capable of operating continuously and have high spatial control, making them advantageous for real‐time adaptive applications. Several advancements we reviewed in magnetic soft robotics are exemplary in their versatility and effectiveness. Joyee and Pan^[^ ^126^ ^]^ developed an inchworm‐inspired soft robot capable of highly controlled linear crawling in confined spaces, showcasing how programmed magnetic domains enable fine‐tuned locomotion without complex onboard electronics. Xu et al.^[^ ^128^ ^]^ introduced a multi‐legged soft crawler, where magnetic field actuation effectively mimics centipedes for robust and high‐speed traversal of diverse terrains. Niu et al.’s MagWorm^[^ ^129^ ^]^ leveraged peristaltic crawling motion to achieve efficient directional crawling, outperforming pneumatic and SMA‐driven soft crawlers in speed and control. Chen et al.^[^ ^131^ ^]^ further expanded the capabilities of magnetic field actuation by introducing a programmable soft robot with low‐melting alloys, alloying dynamic reconfiguration of locomotion modes, and showcasing potential in multi‐functional, adaptable robotics.

Magnetic field‐actuated soft robots often utilize materials with embedded magnetic particles or magnetorheological fluids (MRF). These materials offer the necessary magnetic responsiveness for controlled actuation. The mechanical properties of these materials, such as elasticity, viscosity, and magnetic permeability, significantly influence the robot's performance. Materials like iron particles or ferrites are commonly used due to their high magnetic permeability and ability to interact with external magnetic fields. The magnetic particle size and distribution within the matrix material (e.g., silicone or elastomers) impact the actuation efficiency, allowing for high‐force output while maintaining flexibility. Magnetic materials with larger particle sizes typically provide higher actuation force but may sacrifice flexibility. The most common fabrication methods for magnetic actuators include 3D printing, molding, and casting. 3D printing enables the precise arrangement of magnetic domains within a soft matrix, optimizing actuation performance. For example, magnetorheological fluids (MRF) can be integrated into soft robots using a casting technique to ensure the fluid is uniformly distributed throughout the material, allowing for adaptive motion based on external magnetic fields. The high magnetic permeability and low viscosity of the material improve the actuation speed and control of magnetic field‐actuated soft robots, contributing to efficient crawling and rapid response times. Magnetic soft robots can rapidly adapt to external environments, such as navigating narrow spaces or climbing surfaces, by adjusting the strength of the applied magnetic field.

Despite its advantages, magnetic actuation still faces challenges. The external magnetic field source requirement limits scalability, and force output diminishes over large distances, which is less effective for applications requiring high‐force actuation. Additionally, material fatigue from repeated magnetization cycles can reduce long‐term durability. However, continued advancements in programmable magnetic alignment, closed‐loop sensor feedback, and hybrid actuation strategies are expected to further expand magnetic soft robots' performance and application scope.^[^ ^136^ ^]^ The combination of high‐speed, wireless control, and precision movement makes magnetic actuation a powerful strategy for biomedical robots, industrial automation, and minimally invasive procedures.^[^ ^137^ ^]^

### 3.4 UV and Visible Light Actuation

The use of ultraviolet (UV) and visible light as a means of actuation has emerged as an innovative approach for driving soft robotic crawlers, particularly those constructed from liquid‐crystalline elastomers. This actuation technology is advantageous because it allows for flexible configurations of the actuation power systems, enabling robotic designs that can adapt to various tasks and environments. The fundamental principle behind this technology lies in the diverse thermal expansion properties of the material layers. When exposed to UV or visible light, different layers of the elastomer experience varying degrees of expansion and contraction, resulting in controlled deformation. This deformation is crucial for achieving the crawling motion in these soft robots, allowing them to navigate a range of surfaces with agility. However, implementing this approach requires high precision in fabricating materials. The effectiveness of the actuation depends significantly on the accurate layering and composition of the materials to ensure the desired performance. Therefore, advancements in material science and manufacturing processes are essential to optimize these soft robotic systems for practical applications.

**Figure 14:**
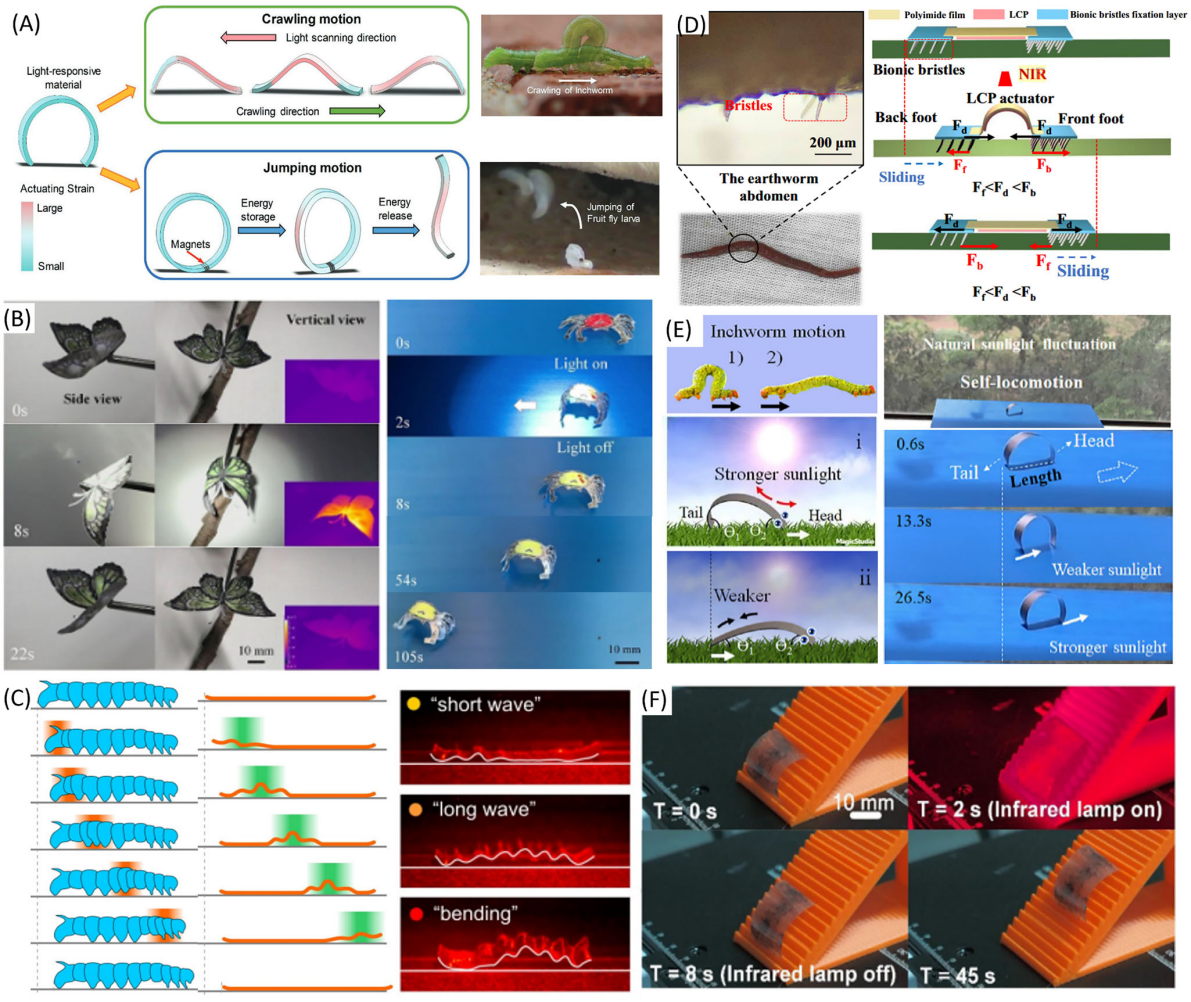
Soft robots actuated by UV and visible light: A) An arch shape soft robot that can crawl and jump, powered by light. Reproduced from Ahn et al.^[^ ^140^ ^]^ Copyright 2019, John Wiley & Sons. B) Artificial crab robot with sideways walking and artificial butterfly with flapping‐wing motion. Reproduced from Hu et al.^[^ ^142^ ^]^ Copyright 2021, John Wiley & Sons. C) Light‐driven soft robot mimics caterpillar locomotion. Reproduced from Rogoz et al.^[^ ^145^ ^]^ Copyright 2016, John Wiley & Sons. D)The motion principle of a soft robot with bionic bristles under photothermal driving. Reproduced from Lou et al.^[^ ^144^ ^]^ Copyright 2024, John Wiley & Sons. E) The design of the inchworm like soft robot with continuous directional crawling motion driven by natural sunlight fluctuation. Reproduced from Hu et al.^[^ ^143^ ^]^ Copyright 2021, ACS Publications. F) Infrared‐driven bio‐inspired soft robot climbing zigzag‐textured sloped structure with crawling gait. Reproduced from Yang et al.^[^ ^146^ ^]^ Copyright 2021, John Wiley & Sons.

A light‐power soft robot composed of liquid crystal elastomer‐carbon nanotube composite, capable of crawling, squeezing through tight spaces, and jumping, was developed by Ahn et al.^[^ ^140^ ^]^ This design utilizes light as a wireless actuation mechanism, where increased light scanning speeds allow adaptation to various terrains and challenges. The robot, as shown in **Figure** **14**A, can crawl at a speed of ≈0.7 mm s^−1^ and reduce its height by ≈25% to navigate narrow channels. Additionally, it can jump to a height of 80 mm and cover a distance of 130 mm in a single leap. This work underlines the potential of light‐driven actuation in achieving adaptive and versatile movements in soft robotic crawlers. Qian et al.^[^ ^141^ ^]^ developed a light‐driven soft robot with twist‐bend actuation for omnidirectional motion. A liquid crystal elastomer with photo‐thermal fillers allows the robot to move in multiple directions and avoid obstacles under various light wavelengths, a novel approach for adaptable locomotion in complex terrains. The robot's movement is achieved through a unique twist‐bend actuation mechanism, where each of its four legs can independently twist and bend under specific wavelengths of light. It can achieve multiple locomotion modes, including turning, straight movement, and rotation, each controlled by targeted light irradiation. For turning, illuminating a single leg with a laser enables directional changes toward northeast, northwest, southeast, or southwest. Straight movement is achieved by simultaneously illuminating two adjacent legs with their corresponding wavelengths, allowing the robot to move west (0.10 mm s^−1^), east (0.08 mm s^−1^), south (0.16 mm s^−1^), or north (0.08 mm s^−1^). Rotation is performed in place by illuminating two diagonal legs, enabling both clockwise (1.1° s^−1^) and anticlockwise (0.7° s^−1^) rotation. The turning speed increases with light exposure duration, with deflecting angle and crawling velocity reaching a saturation point after 8–10 s.

Hu et al.^[^ ^142^ ^]^ designed a self‐oscillating, light‐driven actuator that allows a biomimetic soft robot to crawl autonomously and change color when exposed to light, as shown in Figure 14B. This actuator is based on a covalently bridged black phosphorus‐carbon nanotube heterostructure, which enables the soft photo actuator to undergo light‐driven reversible deformation with a rapid and significant response. Utilizing thermal effects and using the actuator as a source of mechanical power, the robot is equipped with a self‐feedback loop to facilitate self‐oscillation. Inspired by inchworms, this soft robot can move autonomously toward a light source. The actuator demonstrates a rapid bending response, reaching an impressive angle of 230° within 3 s of light exposure, with a bending curvature of up to 1.9. The actuator's design flexibility and ability to incorporate thermochromic materials make it suitable for constructing biomimetic robots, such as crabs that walk sideways and change color and butterflies that flap their wings. They also created a light‐driven actuator (see Figure 14E) using MXene films with asymmetric microstructures, which respond to sunlight for autonomous directional motion without external control.^[^ ^143^ ^]^ Such microstructure can harness natural sunlight to achieve directional self‐locomotion in soft robots, intelligent solar‐energy devices, and wearable clothing. This design shows that light‐based actuation can drive adaptive movement in soft robots for sustainable energy applications. The inchworm‐like soft robot uses ambient natural sunlight fluctuation as its energy source. It achieves directional locomotion by harnessing the changes in sunlight intensity. As sunlight strengthens, the robot stretches upward; an asymmetrical design causes the head to move forward while the tail remains stationary. When sunlight weakens, the robot bends downward, and again, due to the design, the head stays put while the tail contracts forward. This continuous cycle allows the robot to crawl forward without any artificial control, covering a distance of 19 mm in 215 s under natural conditions. The robot can also be controlled manually by blocking and unblocking sunlight, and it is strong enough to carry objects weighing 1.7 times its weight.

Inspired by an earthworm bristle structure, Lou et al.^[^ ^144^ ^]^ created a photothermal‐driven soft robot, as shown in Figure 14D. This robot uses liquid crystal polymers to achieve unidirectional crawling under near‐infrared light, adjusting speed depending on the light irradiation distance. The robot's body consists of multiple segments, including a central photothermal‐driven liquid crystal polymer (LCP) actuator and two bionic bristle units. The angled bristles are designed to create differential resistance during movement; when the robot attempts to slide backward, the bristles dig into the surface, increasing resistance, while forward sliding encounters much less resistance. The LCP actuator contracts when exposed to near‐infrared (NIR) light and relaxes, returning to its original shape when the light is turned off. With an optimized setup of a 5 cm irradiation distance and a 3 cm long LCP actuator, the robot can achieve a maximum average crawling speed of 4.4 mm min^−1^. This design demonstrates how photothermal actuation produces steady crawling motion in soft robots for confined spaces and varied environments. Inspired by caterpillar locomotion, Rogóż et al.^[^ ^145^ ^]^ designed a soft robot that used a liquid crystalline elastomer to achieve sequential, wave‐like deformations under light, as shown in Figure 14C. The caterpillar‐inspired soft robot exhibits impressive performance capabilities. By utilizing a laser beam scanned along its body, the robot generates a traveling deformation wave that mimics the locomotion of real caterpillars. This mechanism allows the robot to achieve various gaits, including “short wave,” “long wave,” and “bending” modes, with speeds ranging from 0.1 to 0.5 mm s^−1^. The robot can successfully navigate challenging environments, demonstrated by its ability to climb an 11‐degree slope at 0.068 mm s^−1^ and squeeze through a 0.9 mm high slit. Furthermore, it can execute tasks such as pushing objects significantly heavier than itself, highlighting its potential for practical applications. Although its step length and speed are notably lower than those of its biological counterparts, the robot's ability to adapt to different terrains and manipulate objects underscores its effectiveness as a miniature soft robot. The robot's performance is limited by the LCE film's damage threshold, which restricts the maximum applicable laser power. By modulating the light field, the robot exhibits crawling and other locomotion modes, such as climbing and object‐pushing capabilities.

An untethered infrared‐driven actuator that enables reversible and reprogrammable behaviors in a soft robot was developed by Yang et al.^[^ ^146^ ^]^ The robot moves using thermomechanical materials sensitive to infrared light, as shown in Figure 14F. This actuator design enables flexible and reversible motion, which could benefit environmental responsiveness and adaptability in soft robotics. The soft robot features an infrared‐driven, untethered, reversible, reprogrammable bilayer actuator made of PLA‐CNT infrared‐responsive and PET elastic layers. The design exploits the layers' mismatched glass transition and melting temperatures for reversible actuation and reprogramming. The crawling speed of this robot is three times faster than that of a typical shape‐memory polymer (SMP)–based light‐driven actuator‐based crawling robot. The actuator can push or carry loads of 0 .455 g. A light‐driven soft robot designed with multidirectional crawling capabilities was developed by Han et al.^[^ ^52^ ^]^ Activated by near‐infrared (NIR) light, this robot harnesses the photothermal response of graphene oxide. It effectively adjusts its movement direction based on the duration of light exposure, achieving omnidirectional control and enabling environmental sensing. This advancement highlights the potential of light‐based actuation to facilitate versatile locomotion without the complexity typically associated with traditional drive systems. Zhu et al.^[^ ^147^ ^]^ have further advanced this area of research with the development of a light‐steered hydrogel‐based biomimetic soft robot. The crawler is capable of crawling, walking, and turning, emulating muscle‐like motion through an anisotropic structure that modifies shape and friction in response to light exposure. The findings demonstrate that integrating light‐driven actuation with friction control can produce robotic systems with high responsiveness and adaptability levels. Gu and Echtermeyer^[^ ^148^ ^]^ also designed photothermal actuators using a composite material of PDMS, graphene, and muscovite mica. These actuators efficiently convert light into motion, enabling small‐scale bio‐inspired robots to achieve reliable and energy‐efficient crawling movements under light stimulation. This innovative approach presents promising avenues for sustainable light‐powered robotics. The actuators can achieve a curvature change of ≈0.76 mm^−1^ in less than 15 s when exposed to a moderate light intensity of 150 mW cm^−2^. The actuators also demonstrate a rapid response time, bending to their maximum curvature in 11 seconds and returning to their original state in 10 seconds. They can carry payloads more than 10 times their weight. For example, the actuator, weighing only 0.003 g, can lift a PDMS cargo 18 times its weight. The actuator's ability to quickly recover from mechanical stress is showcased by its ability to return to its original curled shape in under a second after being flattened by a 90 g beaker.

Xiang et al.^[^ ^149^ ^]^ designed a robot inspired by the mudskipper capable of rapid cyclical movement when exposed to near‐infrared light. The robot can crawl and perform various modes of movement, highlighting its multifunctional capabilities. Such adaptability, governed by light modulation, offers exciting applications in environmental exploration and beyond. The robot's bending motion in the air is rapid, achieving 110° in 1.26 s and recovering in 2.88 s. At the liquid/air interface, it reaches a swimming speed of 28 mm s^−1^, powered by the Marangoni effect. The robot can jump from ethanol solution into the air, reaching a height of 143 mm with a speed of 2 m s^−1^. This jumping ability, driven by bubble formation and bursting, sets it apart from many existing robots. The results also highlight the robot's capacity for controlled movement along pre‐defined paths and its ability to carry loads 30 times its weight while swimming.

UV and visible light actuation provide significant advantages for bio‐inspired soft robotic crawlers due to their wireless, contactless control and adaptability in confined or sensitive environments. Light‐responsive actuators, such as photo‐thermal or liquid crystal elastomers, enable soft robotic crawlers to perform complex and adaptive movements without requiring physical connections to power sources or control units.^[^ ^140^, ^141^ ^]^ These actuators are particularly suitable for small‐scale robotics in biomedical applications or remote environmental monitoring, where precise wireless control is critical. Despite their versatility, light‐driven actuators have limitations, primarily due to the high energy required to induce substantial deformations, a limit to efficiency and endurance. Additionally, response times for light‐driven systems can vary with environmental conditions, for example, lighting intensity or wavelength, potentially reducing consistency in operation.^[^ ^142^, ^145^ ^]^ Light‐responsive materials also have limited penetration depth, where thicker or embedded structures may not respond uniformly to light stimuli. Ongoing challenges in UV and visible light actuation include optimizing light sensitivity and energy efficiency while ensuring durable and repeatable performance. Developing materials that can respond quickly and consistently to a range of light intensities is crucial, as is for enhancing penetration depth for actuation across different structural configurations.^[^ ^94^, ^150^ ^]^ Additionally, integrating light‐actuated systems with other stimuli‐responsive components could yield hybrid designs, improving adaptability and control precision in diverse environments. **Table** **7** summarizes the soft robots actuated by UV and visible light.

**Table 7:** Summary of soft robots actuated by UV and visible light.

Reference	Actuation mechanism	Robot design			Locomotion and speed	Key features
		Size	Weight	Load capacity		
Ahn et al.^[^ ^140^ ^]^	light‐powered bending through an LCE‐CNT composite film	/	/	/	Crawling: about 0.7 mm s^−1^ Squeezing: can reduce height by about 25% to pass through a narrow channel Jumping: maximum height 80 mm; maximum distance: 130 mm	Simple arch shape Can be easily controlled remotely with high spatial and temporal resolution Light sources are generally inexpensive and easy to transport
Qian et al.^[^ ^141^ ^]^	Powered by the photothermal response of a Liquid Crystal Elastomer (LCE) combined with carbon nanotubes (CNTs) with a twist‐bend actuation mechanism	the robot's legs: 1.0 cm long, 0.20 cm wide, and 590 µm thick	/	/	Directional crawling: West: 0.10 mm s^−1^; East: 0.08 mm s^−1^; South: 0.16 mm s^−1^; North: 0.08 mm s^−1^ Rotation: 1.1° s^−1^ clockwise and 0.7 ° s^−1^ anticlockwise Turning	Turning in the northeast, northwest, southeast, and southwest Directional crawl in west, east, south, and north Clockwise and anticlockwise rotation Untethered Control twist‐bend actuation allows for a greater degree of freedom and more versatile and complex locomotion suitable for narrow and delicate environments
Hu et al.^[^ ^142^ ^]^	Photothermal conversion through black phosphorus (BP)‐carbon nanotubes (CNTs)	/	/	/	Light‐guided self‐regulating locomotion Sideways movement Flapping‐wing motion	Rapid bending response, achieving a significant bending angle (230°) within 3 s of light exposure Bending curvature reaches 1.9 cm^−1^ Maintains consistent performance over 100 cycles of repeated light exposure
Hu et al.^[^ ^143^ ^]^	Actuated by natural sunlight fluctuations: I‐MXene film + Polyethylene (PE) film	/	/	Can carry objects weighing 1.7 times its weight	Crawl forward at about 0.088 mm s^−1^	Achieves directional locomotion by harnessing the natural fluctuations in sunlight intensity Manual control
Lou et al.^[^ ^144^ ^]^	Photothermal bending of the LCP actuator under near‐infrared light, combined with angled bristles	/	/	/	Crawling: a maximum speed of 4.4 mm min^−1^	Unidirectional Movement Modulated crawling speeds by manipulating NIR irradiation distance and LCP actuator length A central photothermal‐driven liquid crystal polymer (LCP) actuator unit and two bionic bristle units
Rogoz et al.^[^ ^145^ ^]^	Light‐driven	/	/	/	Walking uses a travelling wave of deformation (average speed of 0.1‐0.5 mm s^−1^) Climbing: can climb an 11‐degree slope Squeezing: can squeeze through a 0.9 mm high slit	Object manipulation: can push a metal cylinder (19.1 mg), which is more than six times the robot's weight The walking speed is much slower than real caterpillars, limited by the absence of a gripping mechanism and the LCE film damage threshold
Yang et al.^[^ ^146^ ^]^	Infrared light driven	/	/	Can carry loads that are ten times heavier than the actuator (typically weigh 0.0455 g)	Crawling Rolling	Can repeatedly flatten and bend its body in a controlled manner Switch between crawling and rolling gaits Multitask, such as gripping objects in confined spaces or navigating complex environments.
Gu and Echtermeyer^[^ ^148^ ^]^	Photo‐thermal bimorph actuators constructed from polydimethylsiloxane (PDMS), graphene (G), and muscovite mica	/	/	More than 10 times their own weight	/	Highly tolerant to mechanical stress: capable of rapidly recovering from extreme deformation, such as being flattened by a weight 22000 times its own. Repeated cycles of actuation and relaxation Fast response: achieving maximum deformation within 10 s of illumination and returning to their original state 5 s after the light is turned off A significant curvature change of 0.76 mm^−1^ at moderate light intensities of 150 mW cm^−2^
Xiang et al.^[^ ^149^ ^]^	Actuated by near‐infrared (NIR) light	/	/	Can carry a weight approximately 30 times heavier than the actuator's weight	Crawling Swimming: maximum swimming speed is 28 mm s^−1^ on the water surface Jumping: a jumping speed of 2 m s^−1^ from an ethanol solution into the air Bending: a bending angle of 110° in the air with a response time of 1.26 s and a recovery time of 2.88 s Rotating	Multifunctional locomotion Multi‐environment adaptability: air, on the liquid/air interface, and in liquids Endures 600 bending cycles without degradation

UV and visible light actuation offer wireless untethered control for soft robotic crawlers, and they are well‐suited for biomedical, environmental, and micro‐robotic applications. By integrating liquid‐crystal elastomers (LCEs), carbon‐based nanomaterials, and photothermal composites, these robots convert absorbed light into controlled deformation and therefore enable adaptive and multi‐modal locomotion. Instead of relying on external fields, light‐driven crawlers can self‐regulate and respond autonomously to natural or artificial light, such as self‐oscillating actuators^[^ ^142^ ^]^ or sunlight‐powered crawlers.^[^ ^143^ ^]^

Representative advancements in light‐driven soft robotics highlight its diverse capabilities. Ahn et al.^[^ ^140^ ^]^ demonstrated a multi‐functional soft crawler that crawls through tight spaces, demonstrating the potential for light‐powered robots in confined environments. Qian et al.^[^ ^141^ ^]^ introduced a twist‐bend mechanism for omnidirectional movement, which shows that light‐controlled soft robots can achieve fine‐tuned, programmable locomotion. Hu et al.^[^ ^142^ ^]^ developed a self‐oscillating light‐driven actuator, demonstrating how light‐responsive robots can achieve autonomous directional crawling. Xiang et al.^[^ ^149^ ^]^ extended light‐actuated robots into multi‐environment locomotion, a robot that crawls using near‐infrared (NIR) light, providing opportunities for robots operating in mixed terrains. However, light‐driven actuation faces key limitations. Response times vary significantly based on light intensity and exposure duration, ranging from 1.26 s bending response to slower 10–15 s deformations. Energy efficiency remains lower than magnetic or chemical actuation, as photothermal conversion involves heat dissipation. Another disadvantage is that light penetration depth limits scalability, making embedded or thick structures challenging for uniform actuation. Ongoing advancements in material design, hybrid actuators, and photonic engineering are expected to enhance response speed, energy efficiency, and scalability, expanding the use of light‐driven soft robots in minimally invasive surgery, environmental sensing, and autonomous bio‐inspired systems.

Liquid crystal elastomers (LCEs) are the primary materials used in light‐actuated soft robots due to their ability to undergo significant shape changes when exposed to specific wavelengths of light. These polymers, when crosslinked and treated with light, exhibit a photo‐responsive behavior that enables controlled bending or twisting. LCEs are known for their anisotropic properties, meaning their deformation behavior varies depending on the direction of light exposure. This property is crucial for programmable and multi‐modal locomotion. Their elasticity allows for large deformations without permanent damage, which is essential for effective crawling. The photo‐thermal conversion efficiency of these materials is enhanced when combined with graphene or carbon nanotubes (CNTs), improving actuation speed and efficiency.

The most common fabrication method for light‐driven actuators involves crosslinking the LCEs under UV light or using thermally induced polymerization processes. Additionally, incorporating graphene or CNTs into the LCEs using composite techniques has been shown to increase their thermal conductivity and actuation efficiency. The photo‐responsive nature of LCEs allows for rapid adaptation to environmental conditions, making them ideal for autonomous robots that operate without external power sources. They can achieve multi‐modal locomotion, such as crawling, turning, and climbing, in response to changes in light intensity or direction, which is particularly useful in environments with fluctuating light conditions or for bio‐inspired soft robots used in remote sensing and search‐and‐rescue missions.

### 3.5 Chemical Actuation

The direct utilization of chemical reactions to produce gas and/or heat, such as monopropellant decomposition, combustion, and hypergolic reactions, has emerged as a promising propulsion strategy for soft crawlers. This innovative approach presents a viable alternative to conventional power sources like built‐in batteries and tethered external power supplies, which often limit the mobility and operational range of these robotic systems. Monopropellant decomposition involves the breakdown of propellant into gaseous products, releasing energy that can be harnessed for movement. Combustion, on the other hand, entails the rapid chemical reaction of fuels with oxidizers, generating heat and pressure that can propel a robot forward. Hypergolic reactions, which occur spontaneously upon contact with the fuel and oxidizer, offer immediate thrust without an ignition source. Henke et al.^[^ ^81^ ^]^ developed a bioinspired soft robot that uses chemical‐based dielectric actuation to produce life‐like, continuous crawling through electromechanical oscillations. Without hard electronic components, the robot driven by dielectric elastomer oscillators generates autonomous crawling motion, showing potential for dielectric elastomer‐based chemical actuation in simplified, responsive soft robots. The electronics‐free soft robot “Trevor” mimics caterpillar crawling using a dielectric elastomer oscillator (DEO) network of DEAs and dielectric elastomer switches (DESs). This system generates a traveling wave of actuation, moving the robot along a rail. Crawling speed increases with voltage, which boosts oscillation frequency and actuation force. The DEO's frequency can be adjusted 20–36 times via resistor modifications and fine‐tuned by voltage changes. Though limited to rail‐guided motion without steering, “Trevor” significantly advances fully autonomous, soft robotics.

A bio‐hybrid robot that integrates skeletal muscle cells within a 3D‐printed soft structure, allowing for directional crawling, was developed by Guix et al.^[^ ^151^ ^]^ The crawler utilizes chemical actuation for muscle contraction, and the muscle cells facilitate autonomous movement through self‐stimulation. The skeletal muscle‐based bio‐hybrid swimmer features a 3D‐printed serpentine spring skeleton and achieves speeds comparable to cardiac‐based bio‐hybrid robots. It outperforms other muscle‐based robots, reaching a maximum velocity of 800 micrometers per second (three body lengths per second) at a frequency of 5 Hz. This design allows for self‐stimulation processes that lead to higher force outputs.

Using cardiomyocytes as the primary actuation source, Sun et al.^[^ ^152^ ^]^ designed a caterpillar‐inspired robot that can contract rhythmically and produce crawling movements. This biohybrid enables efficient crawling in narrow spaces guided by microfluidic channels. The cardiomyocyte‐driven robot uses asymmetric claws, inspired by snakeskin, to generate anisotropic friction for directional movement. The claws, combined with a rough substrate, allow the robot to crawl forward even on an incline of 10°. However, the robot is unable to move up a 20° incline due to its weight. The robot's speed is influenced by the concentration of isoproterenol, a drug that enhances myocardial contractile force. Higher concentrations of isoproterenol result in increased crawling speed. The structural color of the robot changes during movement and in response to drug stimulation, providing a visual indication of the robot's motion status and the cardiomyocytes’ activity.) The potential of cellular chemical actuation offers an effective screening platform for clinical diseases, facilitating autonomous and adaptable locomotion for biomedical applications.

Lv et al.^[^ ^153^ ^]^ designed a chemically actuated soft robot combining liquid metal and liquid crystal networks. This actuator is highly responsive to photothermal and electrochemical stimuli, allowing it to execute intricate shape transformations for versatile adaptive movement. Its design includes advanced programmable crawling capabilities, enabling it to navigate different surfaces seamlessly. Additionally, the actuator demonstrates shape‐morphing abilities, which can be further enhanced by chemical actuation. This capability makes it well‐suited for dynamic adaptability in a variety of environments, effectively responding to changing conditions and stimuli. The LM‐LCN soft robot uses colloidal LM ink with carboxylated gold nanorods (MiniGNR‐COOH) to achieve programmable shape‐morphing and actuation under NIR light. The ink enhances the photothermal properties, allowing selective deposition on LCN films to create different shapes and actuation patterns. The robot can perform different tasks, such as a self‐sustained oscillation at a frequency of 8.0 Hz, inchworm‐inspired crawling on a ratcheted substrate, and participating in robotic Shadow Play by combining with shape memory polymers. Li et al.^[^ ^154^ ^]^ developed a quiet and lightweight crawling robot driven by the liquid–vapor phase transition of ethanol. This chemical actuation mechanism provides silent movement with low energy consumption, an effective approach for chemical‐driven soft robotics in confined or noise‐sensitive environments. The ethanol‐actuated soft crawling robot uses a liquid–vapor phase change mechanism for locomotion, with a central part that expands and contracts head and tail sections utilizing variable friction. Actuators made from silicone and liquid metal enhance thermal conductivity and actuation speed, achieving optimal performance with a 20% liquid–metal ratio. The robot moves at 3.04 mm min^−1^ speeds on horizontal surfaces, 7.21 mm min^−1^ in pipes, and 3.68 mm min^−1^ up vertical sticks. Its variable friction mechanism adjusts based on the surface, enabling multi‐modal locomotion. However, the robot's actuators suffer from weak durability, stability, and repeatability due to ethanol evaporation and microcracks in the silicone, making it slower and less stable than pump‐actuated robots.

**Table** **8** summarises the soft robots actuated by chemical stimuli. Chemical actuation offers unique benefits for bio‐inspired soft robotic crawlers, enabling autonomous, flexible movements through direct chemical reactions without rigid electronic components. This mechanism is particularly advantageous for navigating complex and confined environments, as it allows robots to respond directly to chemical cues in the surroundings, making it suitable for biomedical and environmental applications.^[^ ^151^ ^]^ However, limitations such as slower response times and reduced durability due to continuous chemical reactions can degrade actuator materials and hinder precise control.^[^ ^152^ ^]^ Future developments in hybrid actuation systems and stimuli‐responsive materials are expected to address these challenges. By enhancing control precision, responsiveness, and material longevity, this actuation strategy holds promise for expanding the application range of chemical actuators, enabling autonomous, adaptable crawlers in settings where conventional actuation technologies are less effective.^[^ ^81^ ^]^

Chemical actuation has introduced a novel approach to soft robotic locomotion, enabling autonomous movement through direct chemical reactions such as monopropellant decomposition, combustion, and biohybrid cellular contractions. Unlike conventional actuation strategies that rely on external power sources or tethered control, chemical actuation provides self‐sustaining propulsion. This allows soft robots to operate in confined or remote environments with minimal external intervention. The unique advantage of this method lies in its ability to generate motion without complex electronics or continuous energy input, making it particularly suitable for biomedical, environmental, and exploratory applications. However, challenges such as slower response times, reaction control variability, and material degradation due to continuous chemical exposure remain key barriers to broader implementation. A range of groundbreaking studies have advanced chemical actuation strategies for soft crawlers, and they are especially important for distinct contributions to the field. Henke et al.^[^ ^81^ ^]^ demonstrated an electronics‐free chemical actuation mechanism using dielectric elastomer oscillators, achieving self‐sustained locomotion in soft robots without the need for rigid controllers. This work presents a significant step towards fully autonomous biomimetic soft systems. Guix et al.^[^ ^151^ ^]^ and Sun et al.^[^ ^152^ ^]^ pushed the boundaries of biohybrid actuation, integrating muscle cells and cardiomyocytes into soft robotic structures to generate self‐driven crawling motions. Their work enhanced robotic autonomy and established a platform for studying drug interactions, disease modeling, and regenerative medicine. Lv et al.^[^ ^153^ ^]^ developed a hybrid electrothermal and photothermal actuator, demonstrating programmable shape‐morphing capabilities. This approach enables adaptive, multi‐modal locomotion, bridging the gap between soft robotics and stimuli‐responsive materials. Li et al.^[^ ^154^ ^]^ designed a liquid–vapor phase transition crawler. This design offers a silent, lightweight, and energy‐efficient alternative for confined space operations. Despite the promising advancements, chemical actuation still faces challenges, including slower response time, variable energy efficiency, and material degradation from continuous chemical reactions. Future development in multi‐stimuli hybrid systems, enhanced control precision, and durable materials will help address those challenges, expanding the practical applications of chemical actuation in autonomous soft robots.

Chemical actuation involves using biohybrids, ionic polymers, and smart hydrogels that respond to chemical stimuli. Biohybrids, such as skeletal muscle cells and cardiomyocytes, are commonly used in chemical actuators to provide self‐sustained motion. For non‐biological actuators, materials such as ionic polymer‐metal composites (IPMCs) or conducting polymers are utilized. These materials often exhibit high contractile forces and large deformations in response to chemical stimuli. Ionic polymers operate through ion migration, which allows for slow but large deformation over a longer duration compared to electronic actuators. The biohybrids offer significant advantages in terms of sustainability and biocompatibility. Fabricating chemical actuators often involves integrating living cells into soft matrices such as PDMS, hydrogels, or silicone. For non‐biological chemical actuators, techniques like casting, extrusion, and electrospinning are used to form ionic conductive networks or hydrogels that can respond to chemical gradients. Chemical actuation, particularly through biohybrid systems, enables autonomous movement without the need for continuous external power. This self‐sustaining capability is crucial for applications in confined spaces or biomedical settings where traditional power sources are impractical. The ability of muscle‐based actuators to provide large contractile forces makes them ideal for tasks requiring high force generation in small or constrained environments.

**Table 8:** Summary of soft robots actuated by chemical stimuli.

Reference	Material and Configuration	Actuation Mechanism	Design			Locomotion and Speed	Key Features
			Size	Weight	Load Capacity		
Henke et al.^[^ ^81^ ^]^	composed of elastomers, poly(methyl methacrylate) (PMMA), and imprinted or brushed carbon electrodes	DEA	/	/	/	/	Electronics‐free Placed on a rail‐like track
Guix et al.^[^ ^151^ ^]^	assemble a cell‐laden circular hydrogel (fibrinogen, thrombin, and Matrigel and laden with skeletal muscle myoblasts) around the skeleton (PDMS)	skeletal muscle cells	12 mm body length	/	/	maximum crawling speed 0.8 mm s^−1^ at 5 Hz	Skeletal muscle–based swimming biobot 3D–printed serpentine spring skeleton Achieve mechanical integrity and self‐stimulation during the cell maturation process
Sun et al.^[^ ^152^ ^]^	constructed using a polyethylene substrate pierced by triangle needles as a template for tilted claws, infused with a magnetic nanoparticle‐doped hydrogel solution, coated with a parallel‐aligned CNT layer, and layered with a methacrylated gelatin (GelMA) pregel solution containing modified extracellular matrix components for biocompatibility	cardiomyocyte	/	/	/	/	Inspired by the snake skin and caterpillars Can run along the tracks and exhibit different running speed based on the stimulus concentrations in the tracks Verified preliminary application for the therapy of hyperkalemia
Lv et al.^[^ ^153^ ^]^	fabricated through drop‐casting of the colloidal LM ink onto the LCN film with the mesogenic molecules aligned in a splayed configuration	electro‐thermal photo‐thermal	/	/	/	maximum crawling speed 1.75 mm s^−1^ at a light intensity of 0.3 W cm^−1^	Inchworm‐inspired soft crawler High photothermal conversion efficiency Achieve shape morphing and sequential self‐folding
Li et al.^[^ ^154^ ^]^	silicone elastomer with Ni–Cr resistive wire inserted into the chamber and 10 vol% ethanol injected	liquid‐vapor changes of ethanol	22 mm (L) x 3 mm (W) x 19 mm (H)	/	/	3.04 mm min^−1^ on horizontal surfaces 7.21 mm min^−1^ in pipes 3.68 mm min^−1^ up vertical sticks.	Earthworms inspired Quiet and lightweight Low energy consumption

**Table** **9** provides a comparative analysis of various actuation strategies, highlighting their advantages, challenges, response time, energy efficiency, scalability, common manufacturing approaches, and applications. The six actuation methods reviewed include pneumatic fluid power, hydraulic fluid power, electric fields, magnetic fields, UV and visible light, and chemical actuation, each offering distinct benefits and limitations based on the application requirements. Pneumatic fluid power actuation is valued for its flexibility, lightweight, and simple design, making it suitable for large‐scale applications like environmental monitoring and confined space exploration. However, its lower power density and limited scalability due to the challenges in miniaturizing components restrict its use in high‐force applications. On the other hand, hydraulic fluid power actuation provides superior power density and precise control, making it ideal for heavy‐load applications such as underwater exploration and industrial automation. However, it is bulky, prone to fluid leaks, and requires regular maintenance. Electric field actuation is known for its rapid response time, low power consumption, and minimal mechanical wear, making it highly efficient for biomedical and microscale robotics. Despite these benefits, it requires high voltage and poses risks of dielectric breakdown. Similarly, magnetic field actuation enables wireless operation and biocompatibility, making it a compelling option for minimally invasive surgery and industrial applications. However, it relies on strong external magnetic fields, which can limit precise control and miniaturization. UV and visible light actuation provide untethered, multi‐modal motion while adapting to environmental conditions. Although this benefits applications like soft biomedical robotics and smart textiles, challenges arise from high energy consumption, slow actuation speeds, and limited light penetration depth. In contrast, chemical actuation facilitates autonomous, self‐sustaining motion with minimal external power requirements, making it particularly advantageous for biomedical and environmental monitoring applications. Nonetheless, it faces issues such as limited durability, slow response times, and decreased efficiency due to challenges in precisely controlling chemical reactions. Overall, each actuation strategy presents a unique set of trade‐offs. While hydraulic and pneumatic systems excel in high‐force applications, electrical and magnetic field actuation are better suited for precision‐driven, small‐scale applications. Light and chemical actuation methods introduce innovative, untethered movement but face challenges in efficiency and control. The choice of an actuation strategy depends on factors such as required force output, response time, scalability, and energy efficiency tailored to the specific needs of the intended applications.

**Table 9:** Comparison of different actuation strategies, manufacturing approaches, energy efficiency, and scalability.

Actuation Strategies	Advantages	Challenges	Response Time	Energy Efficiency	Scalability	Common Manufacturing Approaches	Applications
Pneumatic fluid power actuation	Flexibility and lightweight Simplicity in design and operation Comparable response time	Lower power density compared to hydraulic systems, limiting force and torque capabilities	Moderate response time, typically in the range of milliseconds to seconds	Generally low energy efficiency due to air compression losses and leakage	Well‐suited for large‐scale applications Limited scalability due to challenges in miniaturizing compressors, valves, and air channels	Lamination and bonding to create multi‐layered flexible pneumatic actuators Additive manufacturing Soft molding and casting	Environmental monitoring Confined‐space exploration Pipeline navigation
Hydraulic fluid power actuation	High power density allows significant force in compact designs, ideal for heavy‐load applications Precise control and positioning for accurate, repeatable movements Durable and reliable	Bulky and heavy Potential for fluid leaks Requires maintenance	Slow response time, depending on fluid viscosity and system size	Moderate efficiency, but energy losses occur due to fluid friction, heat dissipation, and leakage More efficient than pneumatics for high‐force applications	High and effective for large‐scale applications Challenging for or miniaturization due to bulky components and fluid management	Additive manufacturing Machining to fabricate high‐pressure components Welding	Underwater exploration Heavy duty crawlers
Electrical field actuation	Fast and precise response Low power consumption Remote and wireless control Minimal mechanical wear	Limited force output compared to hydraulics Requires high voltage Dielectric breakdown risk	Fast response time, typically in the millisecond range	High efficiency, especially for small‐scale applications	Scalable across micro‐ to macroscale applications Require high voltage for strong actuation	Printing and coating Thin‐film deposition Microfabrication	Biomedical robots Micro robot in surgery and medicine delivery
Magnetic field actuation	Wireless power and mobility Biocompatible energy source Suitable for in vitro, in vivo, and microfluidic applications.	Limited force output compared to hydraulics Requires strong magnetic fields Sensitive to external interference	Moderate to fast response time, typically in the millisecond range	High efficiency, especially in electromagnetic actuators with optimised coil design	Scalable from microscale (magnetic microrobots) to macroscale (industrial automation) Requires external magnetic fields, which may limit miniaturization and precise localised control	Laser processing to shape and structure magnetic micro‐actuators Sintering and casting Injection and molding to manufacture polymer‐based magnetically responsive materials	Biomedical robots Industrial inspection Minimally invasive surgery Pipeline navigation
UV & Visible Light Actuation	Untethered actuation Multi‐modal and self‐regulated motion Environmental adaptability	High energy consumption Slow actuation Limited penetration depth	Variable response time Fast 1.26s Slow 10∼15s	Low: heat dissipation limit efficiency	Limited by light penetration depth (effective for thin‐film actuators)	Liquid‐crystal elastomers (LCEs) Graphane or CNT‐based photothermal films Bi‐layer actuators with PDMS composites	Soft biomedical robots Environmental sensing Autonomous light‐driven crawlers Smart textiles
Chemical Actuation	Autonomous and self‐sustaining Minimal external power required Bio‐compatible	Short durability Slow response time Variable efficiency Lack precise reaction control	Ms ∼ s ∼ min	30–80% (varies by reaction type)	Mainly effective for small‐scale robots	Biohybrid integration Liquid‐phase actuation Dielectric elastomer oscillators	Biomedical applications Environmental monitoring Confined‐space exploration

## 4 Conclusions and Future Perspectives

We provide insights into future directions and opinions on the major challenges faced by researchers in the field of material science, actuators, and robotics. There is potential to develop soft crawlers that are fabricated using multifunctional materials and actuated by combining different crawling mechanisms. These challenges indicate new opportunities for future efforts in the modeling of motion of soft crawlers, smart materials development, and further understanding of the response of such systems using a range of new and bioinspired crawling mechanisms.

**Figure 15:**
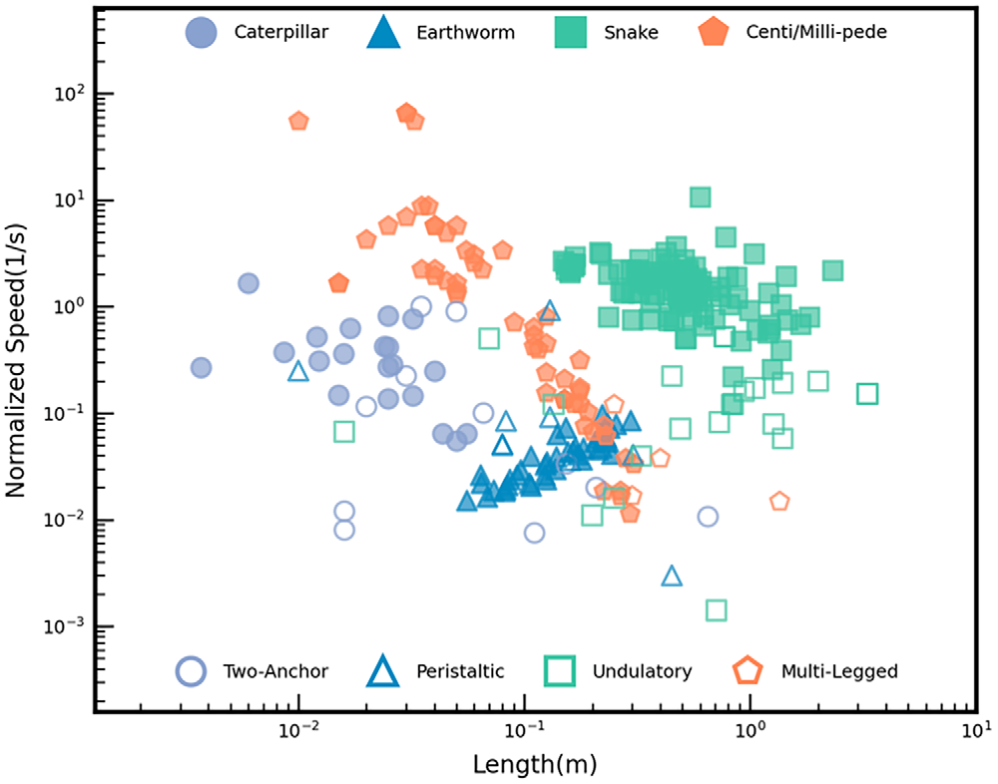
Comparisons between the natural crawling animals using the two‐anchor, peristaltic, and undulatory crawling mechanisms (caterpillars, earthworms, and snakes) and the soft robotic crawlers.

The future of bioinspired soft robotic crawlers lies in advancing materials and actuation systems through hybrid approaches, advanced modeling techniques, and the integration of biological components. These innovations will help address the current scalability, response time, energy efficiency, and material durability challenges, making soft robots more practical for biomedical applications, environmental monitoring, and autonomous exploration. Addressing energy efficiency, material degradation, and scalability challenges in actuator technologies requires a multifaceted approach, combining advancements in materials science, energy harvesting, and system integration. Future research could prioritize the development of high‐efficiency, self‐sustaining actuators that minimize energy loss while maintaining long‐term functionality. By leveraging innovations in nanomaterials, self‐healing mechanisms, and photonic or magnetic engineering, actuators can become more durable, efficient, and scalable. It is essential to focus on enhancing material durability through the application of advanced composites and self‐healing polymers. Additionally, optimizing energy efficiency through the implementation of hybrid systems and improving scalability by advancing light conduction methods and miniaturized magnetic actuators are critical areas that warrant attention in research efforts. These advancements will enhance the performance of soft robotics and automation systems and pave the way for more sustainable and versatile actuation technologies across various applications. Scientists and roboticists have explored and created soft materials and robots in this last decade to mimic the functionality and behavior of soft crawlers inspired by soft‐bodied crawling animals. Such breakthroughs have originated from the unique ability of soft structures to crawl effectively in complex natural environments. Despite such achievements, the increasing demand for portable, flexible, and self‐powering devices for the Internet of Things and for artificial intelligence applications requires additional research effort aimed at creating and discovering new materials and processing methods capable of enhancing a few key aspects:

- Accurately mimicking the crawling mechanisms for developing soft robotic crawlers. Natural crawling animals provide valuable templates for the creation of soft crawlers to achieve crawling without the need for rigid joints or complex control systems. We learned the templates and applied them to developing soft crawlers. However, our state‐of‐the‐art robotic crawlers are not very close to our natural crawling counterparts, as shown in **Figure** **15**. For all three major crawling mechanisms, the performance of human‐made soft robotic crawlers is not well‐matched with their natural counterparts. The average normalized speed of the natural caterpillar family using a two‐anchor crawling mechanism is about 0.3 s^−1^. In contrast, robotic crawlers move at an average normalized speed of 0.03 s^−1^, which is approximately one order of magnitude slower than the natural crawlers. On the other hand, the average length of the robotic crawlers is about 0.1 m, which is longer than the natural crawlers, with an average length of ≈0.025 m. This is due to our flexibility and capability to create and design longer robotic crawlers for various applications, ranging from 0.015 to 0.7 m. The longer crawlers might compromise their crawling speed. The robotic crawlers are able to achieve a very similar maximum normalized speed of about 1 s^−1^ to the natural crawlers, sharing a very similar body length of about 0 .03m. The average normalized speed of natural and robotic crawlers that use the peristaltic crawling mechanism (e.g., earthworm‐like) is ≈0.03 s^−1^. In nature, the earthworm‐like crawlers are approximately one order of magnitude slower than caterpillar‐like crawlers. This phenomenon occurs due to the wave‐like muscle contractions utilized by certain biological organisms to navigate through confined spaces. The locomotion involves sequential contractions and relaxations in a linear, rhythmic pattern, where different segments of a structure contract and expand, propelling the body forward or backward. The robotic crawlers that use the peristaltic crawling mechanism perform the same range of locomotion speed from 0.003 to 0.1 s^−1^ but with a possible longer length of 0.45 m, compared with the natural counterpart. Natural crawlers using the undulatory crawling mechanism move at the fastest speed of about 4 s^−1^, while robotic crawlers using the same mechanism move much slower, with an average speed of 0.04 s^−1^, which is two orders of magnitude slower than the natural crawlers.
- Modeling of motion of soft crawlers. Soft crawlers use body deformation to crawl; therefore, accurately modeling and understanding crawler motions are very important to both the insight of the natural crawlers and the development of soft robotic crawlers. Soft robotic crawlers are typically constructed from soft materials like silicone, elastomers, or hydrogels, which display highly nonlinear stress‐strain relationships. Accurately modeling these materials requires advanced constitutive models considering large deformations, viscoelasticity, and time‐dependent properties. Particularly challenging is ensuring that soft material deformation is accurately represented under various loading conditions. In addition, time‐dependent behaviors such as creep or hysteresis need to be accurately captured and modeled to ensure accurate modeling of the robotic crawlers. When the crawlers perform large rotations, displacement and geometric nonlinearities occur, which may cause complexities in modeling and high computational requirements. Currently, the finite element method (FEM) is widely used to model soft robots. They provide good modeling results, but they can be computationally intensive, especially for large and complex soft robot models that exhibit significant non‐linear deformations. The FEM simulations require a fine mesh to accurately represent small‐scale features, which often results in substantial processing time and computation requirements. Modeling real‐world and robot interactions, such as the interaction of crawlers or robots on various surfaces or within different mediums, presents significant challenges when employing FEM modeling. The FEM often faces challenges for soft crawlers that engage in continuous contact with their environment. FEM can have difficulty accurately modeling dynamic friction, which varies during the interaction, as well as adhesion, which involves the sticking and releasing of surfaces. Additionally, FEM may have difficulty effectively simulating multi‐body dynamics, where multiple interconnected components interact with each other in real‐time. FEM models require careful calibration to ensure they accurately reflect empirical data. Minor inaccuracies in measuring material properties can lead to significant deviations in predictive results. Soft crawlers usually require control systems for real‐time navigation and movement, while FEM modeling is not well‐suited for generating rapid feedback for real‐time control systems.
- Smart materials development and understanding of the material characteristics and responses of soft crawlers for enhancing the capabilities of soft crawlers. The materials used for fabricating the soft bodies of the crawlers define the crawlers’ adaptability, durability, and control precision we can achieve. Materials must exhibit high flexibility and resilience to endure the repetitive deformation of soft crawlers. Hyperelastic materials, such as specific silicones and urethanes, are often chosen for their capabilities to stretch without experiencing permanent deformation. Electroactive polymers change shape or size when they are exposed to electric fields. They are often selected to produce smooth, natural movements, and their responsiveness can be precisely adjusted by varying electrical inputs, allowing for accurate control of the crawlers. They may have high power demands, limiting their applications in soft crawlers. Developing low‐power crawlers or enhancing energy efficiency through energy‐harvesting mechanisms are research directions to explore to tackle this challenge. To improve long‐term durability, smart materials with self‐healing properties, like certain hydrogels or polymers infused with healing agents, can significantly enhance the resilience of soft crawlers. These advanced materials are especially effective at reducing wear and tear during extended use, particularly in challenging environments. Smart materials can deteriorate over time due to environmental factors such as UV exposure, moisture, or repeated deformation. This frequently occurs with soft crawlers during repeated crawling. Consistent and reliable material properties are essential for developing dependable soft crawlers.
- Fabrication and manufacturing of soft crawlers. To ensure the crawlers fulfill their performance in real‐world applications, the manufacturing process of soft crawlers requires precision, versatility, and scalability. Conventional molding and casting techniques are utilized for soft crawlers due to their possibility to create complex, seamless shapes from soft materials. These methods are effective and cost‐efficient for designing and iterating simple crawlers but could be less effective for complicated designs or multi‐material configurations. Additive manufacturing allows for creating complex geometries and facilitates the integration of multiple materials within a single structure. They could fabricate components with varying stiffness and elasticity to mimic natural crawlers accurately. This is a promising research direction to explore and could lead to the development of bio‐inspired and multifunctional crawlers. For micro‐scale soft crawlers, soft lithography enables precise small‐scale fabrication using elastomeric materials, which are not easy to achieve using molding, casting, or additive manufacturing techniques. The hybrid manufacturing technique, which integrates additive manufacturing, molding, and lithography, is promising and has become more compatible with fabricating soft crawlers. Different techniques can be used to fabricate different parts of the soft crawlers. This enables the fabrication of complicated geometries of the crawler bodies and varying stiffness and elasticity without losing body flexibility. On the other side, bio‐inspired fabrication is an alternative approach for developing high‐performance soft crawlers. Mimicking biological structures, such as muscle fibers or tendons, leads to innovative designs that utilize anisotropic materials and multilayered structures. The development and manufacturing of standardized components or modules, such as actuators, sensors, and control units, for soft crawlers presents an opportunity to streamline the manufacturing process across various applications. Implementing modular designs facilitates the customization and repair of individual soft components, thereby reducing the necessity for complete device replacements. This approach enhances operational efficiency and contributes positively to sustainable manufacturing in soft robotics.
- Accurate and robust control of soft crawlers. Controlling soft crawlers poses significant challenges due to their inherent flexibility, nonlinear dynamics, and highly deformable structures. The development of effective control systems for these crawlers necessitates the integration of precise material modeling, accurate sensory feedback, and real‐time control mechanisms. This approach enables the crawlers to navigate and adapt to diverse environments effectively and efficiently. Open‐loop control functions without real‐time feedback, relying on predetermined actuation sequences. This method is particularly applicable for tasks where precise positioning is not essential or when the crawlers are anticipated to operate within a predictable environment. Closed‐loop control utilizes real‐time feedback from sensors, such as strain, pressure, or visual inputs, to dynamically adjust actuation. This methodology permits enhanced precision in control and adaptation, and it is particularly effective for complex or unstructured environments where the crawler must promptly respond to variations in its surroundings. Proportional‐integral‐derivative (PID) controllers are extensively employed in the operation of soft crawlers, attributed to their simple design and effectiveness across a range of applications. These controllers function by continuously adjusting the output based on the proportional (P), integral (I), and derivative (D) errors between the desired demand and the actual measured responses. Turning the PID parameters, including proportional gain, integral time, and derivative time, can present significant challenges in soft crawlers. This challenge stems from the nonlinear and unpredictable deformation characteristics of soft materials and bodies. As a result, the crawlers' dynamic response may differ under various operational conditions, making it difficult to achieve their optimal performance. To address these challenges, a careful approach to parameter turning is essential to ensure that the controller can effectively respond to the unique dynamics of soft crawlers while maintaining stability and precision in their operation. Machine learning models show promise in predicting the behavior of soft crawlers by analyzing large datasets. This analysis enables more accurate and effective control over their complex and non‐linear dynamics. Machine learning models learn from experiences and environmental interactions, which makes them increasingly sophisticated in understanding the movement patterns of soft crawlers. Reinforcement learning enables crawlers to optimize their movements by receiving feedback from their interactions with the environment. For example, when a crawler encounters obstacles or varying terrain types, it adjusts its strategy based on whether those interactions lead to successful navigation or failure. This adaptive learning process becomes especially valuable and useful when the crawlers are navigating an unknown or unpredictable environment where conventional control methods may be ineffective. Advanced control systems are increasingly employing real‐time simulations that accurately represent both the physical structure of soft crawlers and their surrounding environment. The control system can dynamically adjust control strategies in response to changing conditions with the predicted results of the crawlers’ movement using simulations. Advanced control systems often include the unique properties of the soft crawlers’ bodies, such as flexibility, responsiveness, and deformation, with comprehensive environmental interaction models. These control systems significantly improve crawler navigation accuracy, enabling smoother and more precise movements across various environments. The implementation of these simulations requires substantial computational resources, which creates challenges for real‐time processing and control.
- Implementing soft crawlers in real‐world scenarios. Soft crawlers employ various actuation methods, including pneumatic, hydraulic, electric, light‐driven, and chemical mechanisms. These approaches can require substantial power sources, which limits robot operational autonomy. To enhance their performance, it is essential to develop lightweight and energy‐dense power systems, such as compact battery technologies, energy‐efficient actuators, or hybrid energy‐harvesting solutions like solar‐assisted systems. In addition, optimizing the properties of materials used can help reduce power consumption. Soft crawlers also pose challenges for achieving precise movement control. Traditional control strategies often struggle to address the nonlinear and highly deformable characteristics of these soft materials.

Recent advancements in artificial intelligence and machine learning present promising solutions. For example, integrating soft sensor technologies can significantly enhance feedback and control capabilities. To further enhance crawler functionality, it is advantageous to implement advanced control algorithms that continually learn from interactions with the environment, thereby improving navigation and operational accuracy. As soft crawlers navigate diverse terrains, they are subjected to continuous deformation and harsh environmental conditions, including uneven surfaces, extreme temperatures, and corrosive materials. Such factors can accelerate material degradation, ultimately restricting the operational lifespan of these systems. To address these challenges, employing innovative materials such as self‐healing polymers and carbon nanotube‐embedded composites can improve resilience and extend durability. Ongoing research into advanced materials holds significant potential for enhancing the longevity and robustness of soft crawlers, enabling them to thrive in demanding environments. Integrating multi‐modal locomotion systems, combining crawling with jumping, gripping, or climbing, provides a versatile approach that greatly enhances real‐world applicability. By harnessing these biomimetic principles, we can develop innovative robotic systems capable of navigating challenging landscapes, overcoming obstacles, and adapting to a variety of tasks with unprecedented efficiency.

Scaling designs of soft crawlers for various applications, from medical devices to industrial inspection, poses challenges in material fabrication, miniaturization, and actuation performance. Advances in additive manufacturing, such as 3D printing of multi‐material structures and bioinspired fabrication techniques, may also facilitate scalable production. Miniaturized soft actuators, powered by electrostatic or microfluidic mechanisms, could assist in achieving efficient small‐scale soft crawling robots. Addressing these challenges requires interdisciplinary efforts spanning materials science, biomechanics, robotics, and artificial intelligence. Soft crawlers can unlock new applications in medical robotics, search‐and‐rescue missions, and industrial automation by improving energy efficiency, control precision, material resilience, scalability, and adaptability. Future research could focus on integrating bioinspired principles with advanced materials and intelligent control systems to realize practical, high‐performance soft robotic crawlers.

In conclusion, the development of soft robotic crawlers, inspired by the adaptive and efficient movements of soft‐bodied animals represents a promising frontier in robotics. Through advancements in flexible materials, an understanding of bioinspired crawling mechanics, and the implementation of sophisticated actuation techniques, researchers are developing robots that can navigate complex environments with unprecedented agility. We reviewed the synergy between materials, design, and actuation, which together enable diverse locomotion styles, from peristaltic to serpentine and undulatory motion. Continued innovation in material science and actuation strategies is crucial in realizing soft crawlers that can perform specialized tasks in medical, environmental, and exploratory applications. Future research has the potential to enhance these capabilities, leading to soft robots that can interact with and adapt to their surroundings with a versatility and resilience similar to their natural counterparts.

## Conflict of Interest

The authors declare no conflict of interest.
